# Can mesenchymal stem cells and their conditioned medium assist inflammatory chondrocytes recovery?

**DOI:** 10.1371/journal.pone.0205563

**Published:** 2018-11-21

**Authors:** Yu-Chun Chen, Yu-Wei Chang, Kinn Poay Tan, Yi-Shan Shen, Yao-Horng Wang, Chih-Hung Chang

**Affiliations:** 1 Department of Orthopedic Surgery, Far Eastern Memorial Hospital, New Taipei City, Taiwan, R.O.C; 2 College of General Studies, Yuan Ze University, Taoyuan City, Taiwan, R.O.C; 3 Department of Surgery, Memorial Mackay Hospital, Taipei, Taiwan, R.O.C; 4 Department of Biomedical Engineering, National Taiwan University, Taipei, Taiwan, R.O.C; 5 Department of Nursing, Yuanpei University of Medical Technology, Hsinchu, Taiwan, R.O.C; 6 Graduate School of Biotechnology and Bioengineering, Yuan Ze University, Taoyuan City, Taiwan, R.O.C; Centro Cardiologico Monzino, ITALY

## Abstract

Osteoarthritis (OA), one of the most common joint disease, affects more than 80% of the population aged 70 or over. Mesenchymal stem cells (MSCs) show multi-potent differentiation and self-renewal capability, and, after exposure to an inflammatory environment, also exhibit immunosuppressive properties. In this study, we have used a model of lipopolysaccharide (LPS)-stimulated chondrocytes to evaluate MSC anti-inflammatory efficacy. The anti-inflammatory mechanism was tested in two cell-contained culture systems: (i) MSC-chondrocyte indirect contact system and (ii) MSC-chondrocyte direct contact system, and one cytokine-only culture system: MSC-conditioned medium (CM) system. Results showed that MSCs reduced chondrocyte inflammation through both paracrine secretion and cell-to-cell contact. The inflammation-associated, and free-radical-related genes were down-regulated significantly in the direct contact system on 24 h, however, the TNF-α. IL-6 were upregulated and aggrecan, COLII were downregulated on 72 h in direct contact system. Moreover, we found CM produced by MSC possess well therapeutic effect on inflammatory chondorcyte, and the 10-fold concentrated MSC-conditioned medium could down-regulated chondorcyte synthesis inflammation-associated, and free-radical-related genes, such as TNF-α, IL-1β, IL-6 and iNOS even treated for 72 h. In conclusion, MSC-CM showed great potential for MSC-based therapy for OA.

## Introduction

Osteoarthritis (OA), one of the most common degenerative joint diseases, affects more than 80% of the population aged 70 or over. The pathology of OA includes progressive hyaline articular cartilage loss, sclerotic changes in subchondral bone, and cyst or osteophyte formation. Pro-inflammatory cytokines, especially interleukin-1β (IL-1β), tumor necrosis factor (TNF)-α, and interleukin-6 (IL-6), which affect both the quantity and quality of the cartilage extracellular matrix (ECM), are important in the pathogenesis of OA. IL-1β and TNF-α stimulate the release of MMP-1, MMP-3, and MMP-13 and downregulate expression of type II collagen and aggrecan in chondrocytes [1–3]. In an animal rheumatoid joint model, IL-6, in combination with IL-1β or oncostatin, upregulated MMP-1 and MMP-13 expression [4]. These cytokines contribute to the pathogenesis of OA by downregulating anabolic events and upregulating catabolic and inflammatory responses, resulting in structural damage to the OA joint.

Recently, more and more research has focused on the anti-inflammatory ability of mesenchymal stem cells (MSCs). After exposure to an inflammatory environment, they could produce very high levels of immunosuppressive factors, such as TNF-α, IL-1β, and interferon (IFN)-γ [5]. Intra-articular injection of MSCs has been suggested as a promising option for OA therapy [5]. In mice, a single intra-articular injection of purified MSCs decreases synovitis and cartilage damage by suppressing synovial macrophage activation [6]. The potential therapeutic efficacy of MSC-based therapies for OA has also been observed in various animal models including sheep, rabbits, minipigs [7–11], and even clinical studies. However, some groups found the MSC injection could retard OA symptom, but some are not. In a randomized, double-blind, phase II clinical study (NCT01453738), Gupta’s group found there was a trend towards improvement was seen in the 25-million-cell dose group in VAS, ICOAP, and WOMAC scores, but the results were not statistically significant improved when compared to hyaluronic acid injection group [12]. In another multicenter randomized controlled knee OA phase I/II clinical trial (NCT02123368), low dose (10 × 10^6^) and high dose (100 × 10^6^) cultured autologous bone marrow MSCs injection was compared. Results showed that the high-dose group exhibited a significant improvement even at 12 months [13].

**Fig 1:**
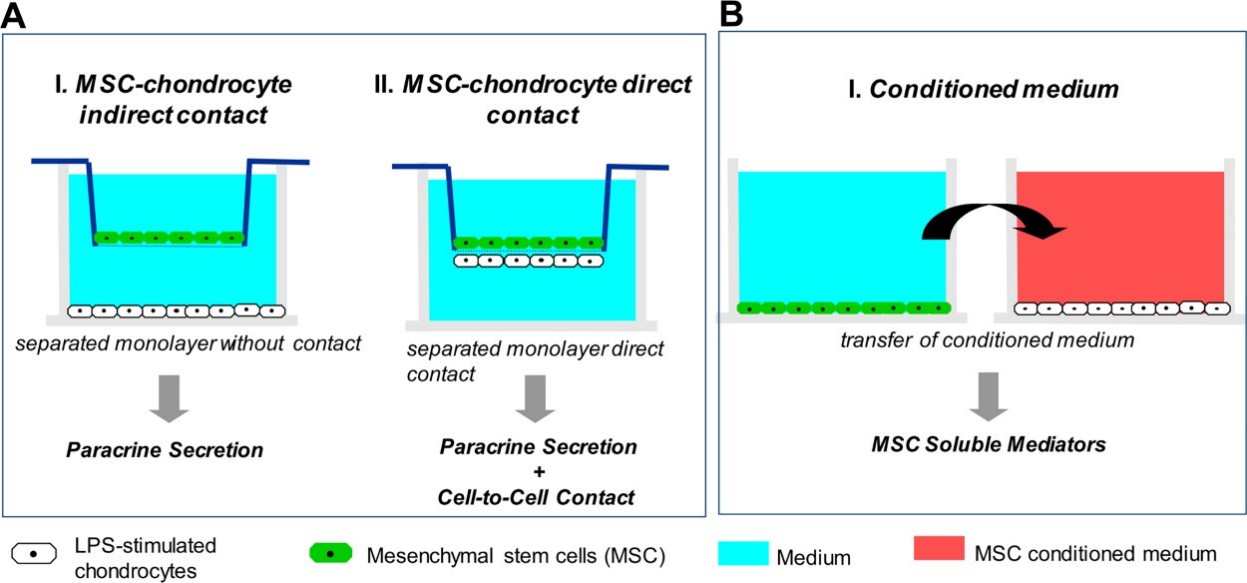
(A) Two cell-contained culture systems: (I) MSC-chondrocyte indirect system for evaluating the paracrine effect of MSCs. (II) MSC-chondrocyte direct contact system to evaluate the combined MSC paracrine and direct cell-to-cell contact effects, and (B) one cytokine-only culture system: conditioned medium system to examine the effect of MSC-produced soluble mediators on chondrocytes were evaluated in this study.

Even though there are various MSC clinical studies conduct, most of them focus on treatment effect evaluation. Thus, in this study, we would like to examine whether the cartilage repair induced by MSC therapy was due to MSC-chondrocyte direct contact, paracrine effects, or a combination of the two. Besides, we also wonder if the treatment effect of MSC would be influence by the MSC-chondrocyte co-culture time. To answer these questions, we used lipopolysaccharide (LPS) stimulation of chondrocytes. LPS, elicits strong immune responses in animals, and can be used in *in vitro* cell culture systems to mimic inflammation situation. We used two cell-contained culture systems: MSC-chondrocyte indirect contact system, and MSC-chondrocyte direct contact system for chondrocyte inflammation level and ECM synthesis evaluation (Fig 1A). Moreover, a cytokine-only culture system: MSC-conditioned medium (CM) system was also been set up to evaluate if the CM possess therapeutic effect on inflammatory chondorcyte and to determine the optimal concentrated fold of CM (Fig 1B).

## Materials and methods

### Materials

The Animal Research was approved by Far Eastern Memorial Hospital IACUC, and the approval number was 102-02-06-A. Unless otherwise stated, all materials used were purchased from Sigma-Aldrich Inc. (MO, USA). Phosphate-buffered saline (PBS), antibiotic-antimycotic solution, trypsin-EDTA, fetal bovine serum (FBS), Dulbecco's modified Eagle's medium (DMEM), low glucose minimum essential medium α (α-MEM), and the Superscript III first strand synthesis system were obtained from Thermo Fisher Scientific (MA, USA), Ficoll-Paque PREMIUM from GE Healthcare Life Sciences (MA, USA), the Transwell kits and the Amicon ultra filter tubes from Merck Millipore (Darmstadt, Germany), and the MirVana miRNA isolation kits from Ambion (Carlsbad, USA).

### Chondrocyte isolation

Fresh porcine knees were purchased from a traditional market and kept skin intact for 2–3 h on ice until harvesting cartilage under aseptic conditions. In brief, full thickness porcine articular cartilage was minced, then washed three times in sterile PBS containing 5% (100x) antibiotic-antimycotic solution and twice in PBS at room temperature, then chondrocytes were isolated by digestion for 18 h with 0.5% collagenase in DMEM supplemented with 10% FBS and 1% (100x) antibiotic-antimycotic solution at 37°C in a 5% CO_2_ incubator. The porcine chondrocytes were then resuspended, and washed twice, in PBS, then were cultured in DMEM supplemented with 10% FBS in 100 mm diameter culture dishes at a density of 5 x 10^5^ cells/dish. The culture medium was changed twice a week.

### Isolation of bone marrow-derived MSCs

General anesthesia was induced in adult pigs (age ~18 mo) using a combination of ketamine (10 mg/kg body weight i.m.) and xylazine (2 mg/kg body weight i.m.) with inhalation anesthesia (halothane), and bone marrow aspirates were obtained from the humeral head using an 11-gauge needle attached to a heparinized syringe using an Animal Care and Use Protocol approved by the Far Eastern Memorial Hospital IACUC (Permit Number: 102-02-06-A). In brief, bone marrow was collected in a heparin-containing tube and 3 ml of the blood-saline mixture layered onto 3 ml of Ficoll-Paque PREMIUM in a 15 ml conical centrifuge tube, which was then centrifuged at 490 g for 30 minutes at room temperature. After centrifugation, the upper layer was discarded, and the opaque interface carefully transferred to a clean centrifuge tube, then 10 ml of low glucose α-MEM was added, and the sample mixed by inversion. After centrifugation at 150 g for 5 min, the supernatant was discarded and the pelleted MSCs resuspended in low glucose α-MEM containing 10% FBS and 1% (100x) antibiotic-antimycotic solution and plated on 10 cm dishes. The medium was changed twice a week.

### Establishment of the chondrocyte inflammation model

Chondrocytes in DMEM containing 10% FBS were seeded in 96-well culture plates at a density of 5×10^3^ cells/well. After 24 h, the medium was replaced with medium containing 0, 2, 20, or 200 μg/ml of LPS for 4 h, when mRNA levels for inflammation-related genes were evaluated, or for 24 h or 72 h, when cell morphology was observed, and cell numbers quantified using the WST-1 assay. Gene expression was calculated from the mean of four samples and cell viability from the mean of five samples.

### Indirect and direct contact MSC-chondrocyte co-culture systems

In the indirect MSC-chondrocyte co-culture system [14], chondrocytes in DMEM containing 10% FBS were allowed to attach overnight at 37°C to the bottom of the outer chamber of a Transwell at a density of 1.5×10^5^ cells/well, then the medium was replaced for 4 h with 2 ml of medium alone or medium containing 2 μg/ml of LPS.MSCs were then added to the upper surface of the Transwellinsert at ratios of MSCs to chondrocytes of 1:1 (M1C1), 1:3 (M1C3), and 1:5 (M1C5) (see Table 1) to study the indirect co-culture effect (Model I in Fig 1A) and the cells cultured for a further 24 or 72 h in the continued presence or absence of LPS, then the chondrocytes were examined. The pore size of the Transwell is 0.4μm, and the insert material is polyester membrane.

**Table 1:** Cell number and MSC-chondrocyte ratios in the indirect co-culture system.

Group	MSC: Chondrocyte Ratio	Cell Number (MSC: Chondrocyte)
**M1C1**	1:1	1.5 x 10^5^ MSCs: 1.5 x 10^5^ chondrocytes
**M1C3**	1:3	5 x 10^4^ MSCs: 1.5 x 10^5^ chondrocytes
**M1C5**	1:5	3 x 10^4^ MSCs:1.5 x 10^5^ chondrocytes

In the direct chondrocyte-MSC co-culture system, which was adapted from those used in previous studies [15, 16], chondrocytes in DMEM containing 10% FBS were seeded for 3 h on the surface of an inverted Transwell insert at a density of 1.5×10^5^ cells/insert, which was then placed in its normal orientation in a Transwell and DMEM containing 10% FBS added and the cells allowed to attach overnight. The next day, the medium was replaced with medium alone or medium containing 2 μg/ml of LPS for 4 h, after which 5 × 10^4^ MSCs were added to the upper surface of the insert (M1C3 group, chondrocyte-MSC co-culture ratio 1:3), then, after incubation for a further 24 or 72 h, the chondrocytes were collected for gene expression analysis (Model II in Fig 1A).

In the “LPS group” in both systems, no MSCs were added and the chondrocytes were incubated with LPS for the whole period until examination.

### Preparation of MSC-CM and effect on LPS-stimulated chondrocytes

MSC-CM was prepared from second and third passage porcine BM MSCs. In brief, the cells were seeded overnight on 15 cm diameter culture plates at a density of 1.5x10^6^ cells/plate in low glucose α-MEM. After 3 washes with PBS, 30 ml of DMEM without FBS was added and the cells cultured for 24 h, then centrifuged at 430 g for 5 min at 4ºC to remove debris. The supernatant was then used as such (CM1X) or concentrated by centrifugation at 4°C at 2330 g in Amicon ultra filter tubes with a 3-kDa cutoff ultra-filtration membrane for about 30 min or 50 min when the sample was concentrated 5-fold (CM5X) or 10-fold (CM10X), respectively, and the pass-through material from the 5-fold concentration collected as PT5X.

Chondrocytes (1.5x10^5^) were plated overnight in the 24-wells, then were incubated with LPS for 4 h. The medium was then removed and replaced with 2 ml of MSC CM1X, CM5X, CM10X, or PT5X. The cells incubated for a further 24 or 72 h, for gene expression and cell viability test (Fig 1B). In LPS group, the medium was also removed after 4 h- LPS treatment and replaced with 2 ml of fresh medium.

### RNA isolation, cDNA synthesis, and real-time quantitative PCR amplification

Total RNA was isolated from the cells using mirVana miRNA isolation kits following the manufacturer’s protocol. First-strand cDNA synthesis was performed on each RNA sample using SuperScript III reverse transcriptase. Quantitative real-time PCR was performed using a LightCycle 480 (Roche, IN, USA). Levels of TNF-α, IL-1β, IL-6, caspase 3, TSG-6, IL-1ra, iNOS, AGG, COLI, and COLII mRNA were then normalized to GAPDH mRNA levels in the same sample and this value expressed as a log_10_ fold value compared to that for the control sample (cells in medium alone) using the Ct method. Each value reported is the mean for four samples.

### Statistical analysis

To ensure reproducibility, gene expression analysis studies were performed 4 times and cell viability studies 5 times. Statistical analysis was performed by one-way analysis of variance (ANOVA) with a p value of ≤ 0.05 being taken as a statistically significant difference.

## Results

### Determination of the optimal concentration of LPS for chondrocyte stimulation

**Fig 2:**
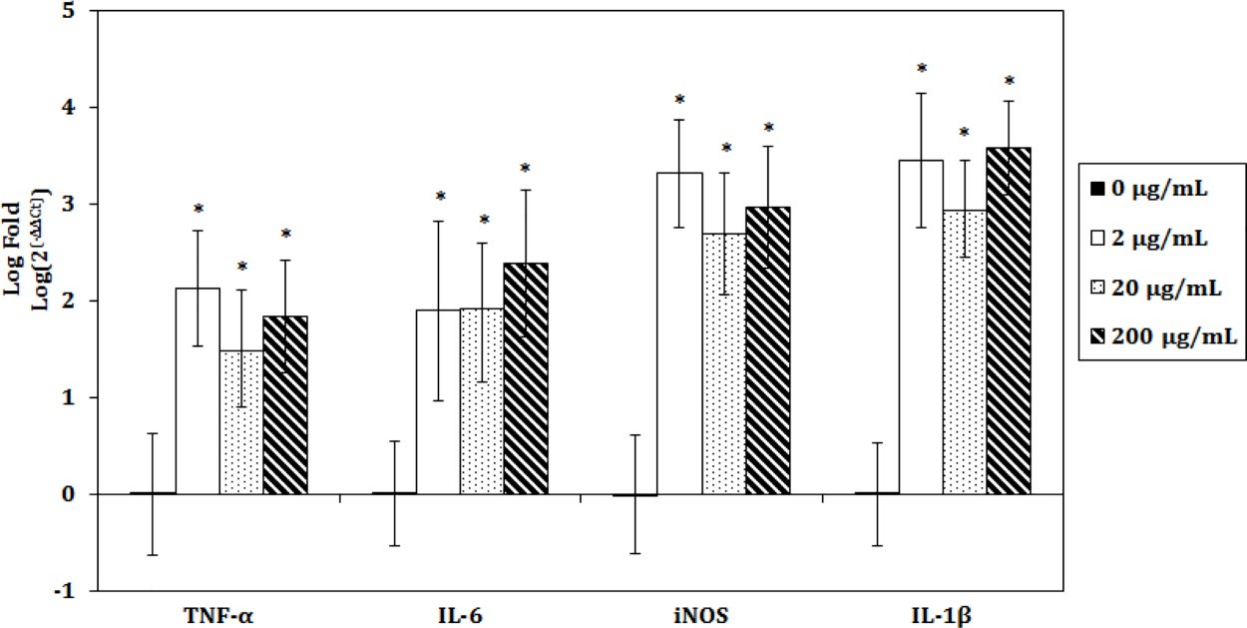
Effects of lipopolysaccharide (LPS) on inflammatory gene expressions on chondrocytes. Chondrocytes were incubated for 4 h in medium alone (control) or medium containing 2, 20, or 200 μg/mL of LPS, then levels of mRNAs for TNF-α, IL-6, iNOS, and IL-1β were determined. The data are expressed as the mean ± standard deviation (n = 4). * p < 0.05, significant difference compared to the control group.

After incubation of chondrocytes for 4 h with 0, 2, 29, or 200 μg/ml of LPS, mRNA was isolated and gene expression evaluated. As shown in Fig 2, in the presence of LPS, a significant increase was seen in mRNA levels, with log_10_ fold values compared to controls incubated in medium alone of 1.48–2.13 for TNF-α, 1.90–2.39 for IL-6, 2.70–3.31 for iNOS, and 2.94–3.59 for IL-1β, with no significant differences between the three groups.

**Fig 3:**
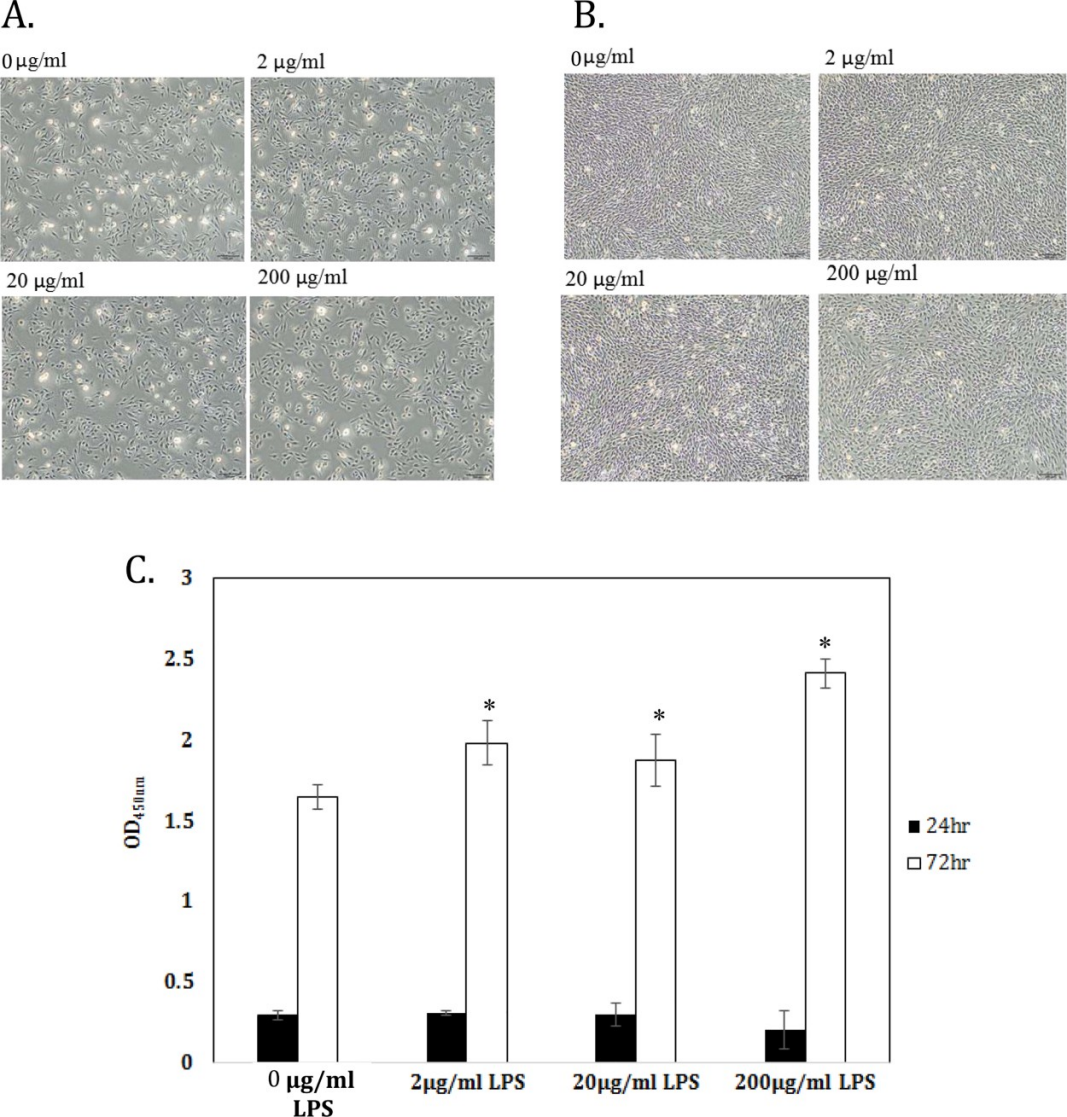
Effects of LPS on chondrocyte morphology and cell numbers. Chondrocytes were incubated with medium alone (control) or medium containing 2, 20, or 200 μg/mL of LPS for 24 h (A and C) or 72 h (B and C), when morphological changes were assessed (A and B) and cell numbers quantified (C). The experiments in A and B were repeated 5 times with similar results. In (C), the data are expressed as the mean ± standard deviation (n = 5). * p < 0.05, significant difference compared to the control group at the same time point.

After LPS treatment for 24 or 72 h, chondrocyte cell morphology was evaluated by microscopy and cell numbers quantified using the WST-1 assay. No difference was seen in cell morphology between untreated chondrocytes and those treated with 2–200 μg of LPS for 24 h (Fig 3A) or for 72 h (Fig 3B). In addition, no difference in cell numbers were seen between the groups at 24 h. However, when the chondrocytes were treated with LPS for 72 h, cell numbers in all LPS stimulation groups were significantly higher than in the control group (all p<0.05).

These results showed that the concentration of 2 μg/mL of LPS was sufficient to increase chondrocyte mRNA levels for inflammation-related genes without affecting cell morphology and this concentration was therefore used in the chondrocyte inflammation model.

### MSC-chondrocyte indirect contact co-culture system

**Fig 4:**
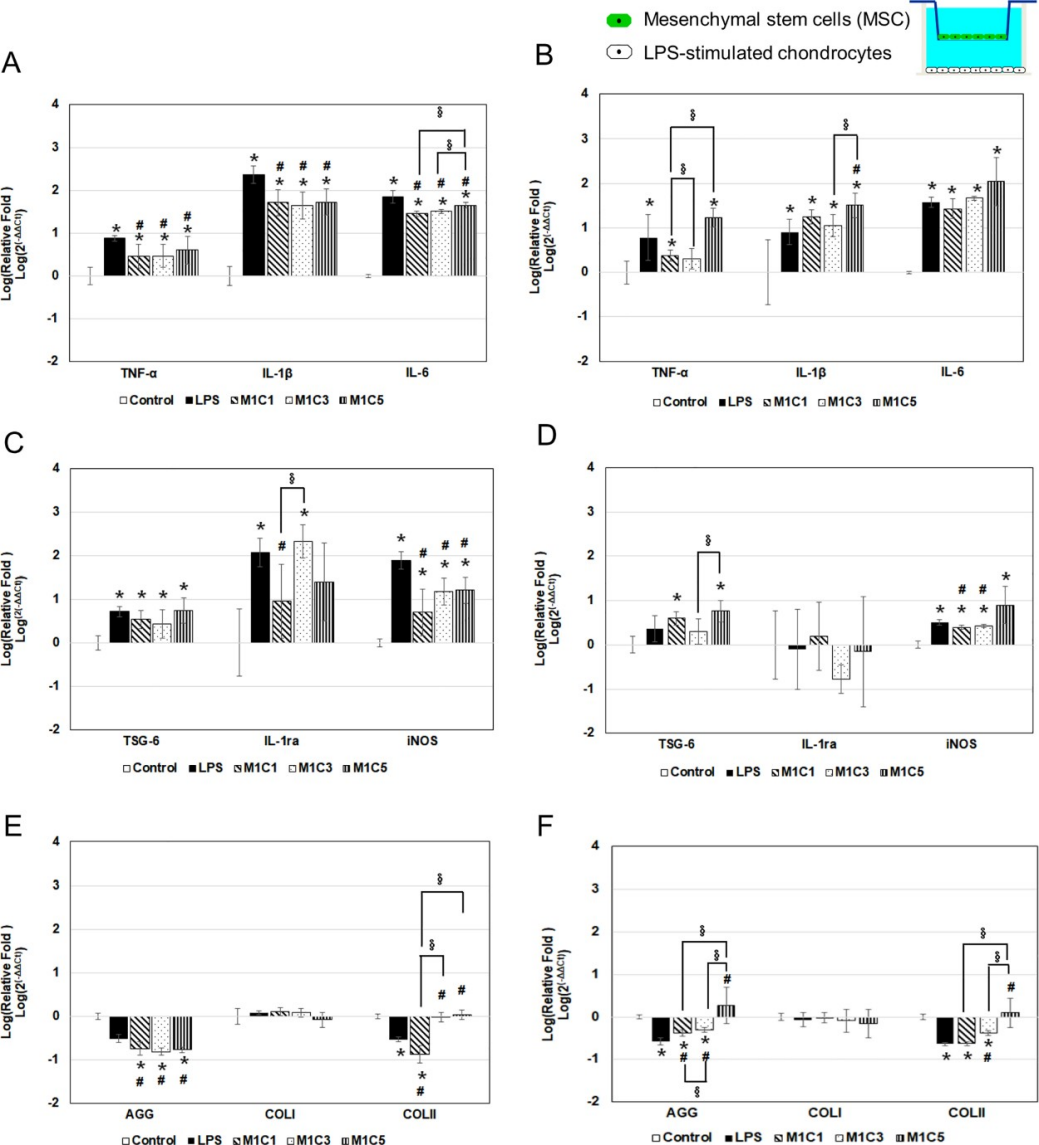
Gene expression in MSC-chondrocyte indirect system in chondrocytes with LPS-induced inflammation. In the control group, chondrocytes were incubated with medium alone. In all of the other groups, the chondrocytes were incubated with 2 μg/mL of LPS group for 4 h, then were incubated for a further 24 h (A, C, E) or 72 h (B, D, F) either in the presence of LPS alone (LPS group) or LPS plus MSCs at different MSC:chondrocyte ratios (M1C1, M1C3, and M1C5 groups). (A, B) are the inflammation-related genes results; (C, D) are anti-inflammation and free-radical-related genes results; (E, F) are ECM synthesis-related genes results. * *p* < 0.05, significant difference compared to the control group. ^#^ *p* < 0.05, significant difference compared to the LPS group. ^§^ *p* < 0.05, significant difference between the indicated groups.

Fig 4 shows the results for gene expression in the indirect contact system when 1.5x10^5^ chondrocytes attached to the bottom of the outer chamber of a Transwell were incubated with or without LPS for 4 h, then 15, 5, or 3x10^4^ MSCs [MSC/chondrocyte ratios of 1:1 (M1C1), 1:3 (M1C3), or 1:5 (M1C5)] and incubation carried out for a further 24 h (Fig 4A, 4C and 4E) or 72 h (Fig 4B, 4D and 4F) in the continued presence or absence of LPS. As a control, chondrocytes alone were incubated without LPS for the entire period.

Incubation of chondrocytes alone with LPS resulted in upregulated expression of the inflammation-related genes TNF-α, IL-1β, and IL-6 (Fig 4A), the free radical-related gene iNOS (Fig 4C), the anti-inflammation-related genes TSG-6 and IL-1ra (Fig 4C), and downregulated expression of AGG and COLII (Fig 4E).

In contrast, at 24 h, compared to the “LPS” group, expression of TNF-α, IL-1β, IL-6, iNOS, and AGG was significantly downregulated in all MSC contained groups (Fig 4A and 4C), while, at 72 h, TNF-α, iNOS, AGG and COLII was also significantly downregulated in M1C1 and M1C3 group compared to that in LPS group (Fig 4B and 4F). But TNF-α, IL-1β, TSG-6, AGG, COLII mRNA was significant upregulated in the M1C5 group (Fig 4B, 4D and 4F).

Since the number of MSCs in the bone marrow aspirate is quite low (about 10–83 MSCs per 10^6^ nucleated cells or 109–664 native CFU-F per ml of tissue [17], and also there are similar results between M1C1 and M1C3 groups (TNF-α, IL-1β, IL-6, and iNOS mRNA levels significantly decreased at 24 h and iNOS levels slightly decreased at 72 h), so in the next study, we use only the M1C3 group to see if less numbers of MSCs still can provide benefit effect to chondrocytes in both indirect and direct contact system.

### MSC-chondrocyte direct contact co-culture system

**Fig 5:**
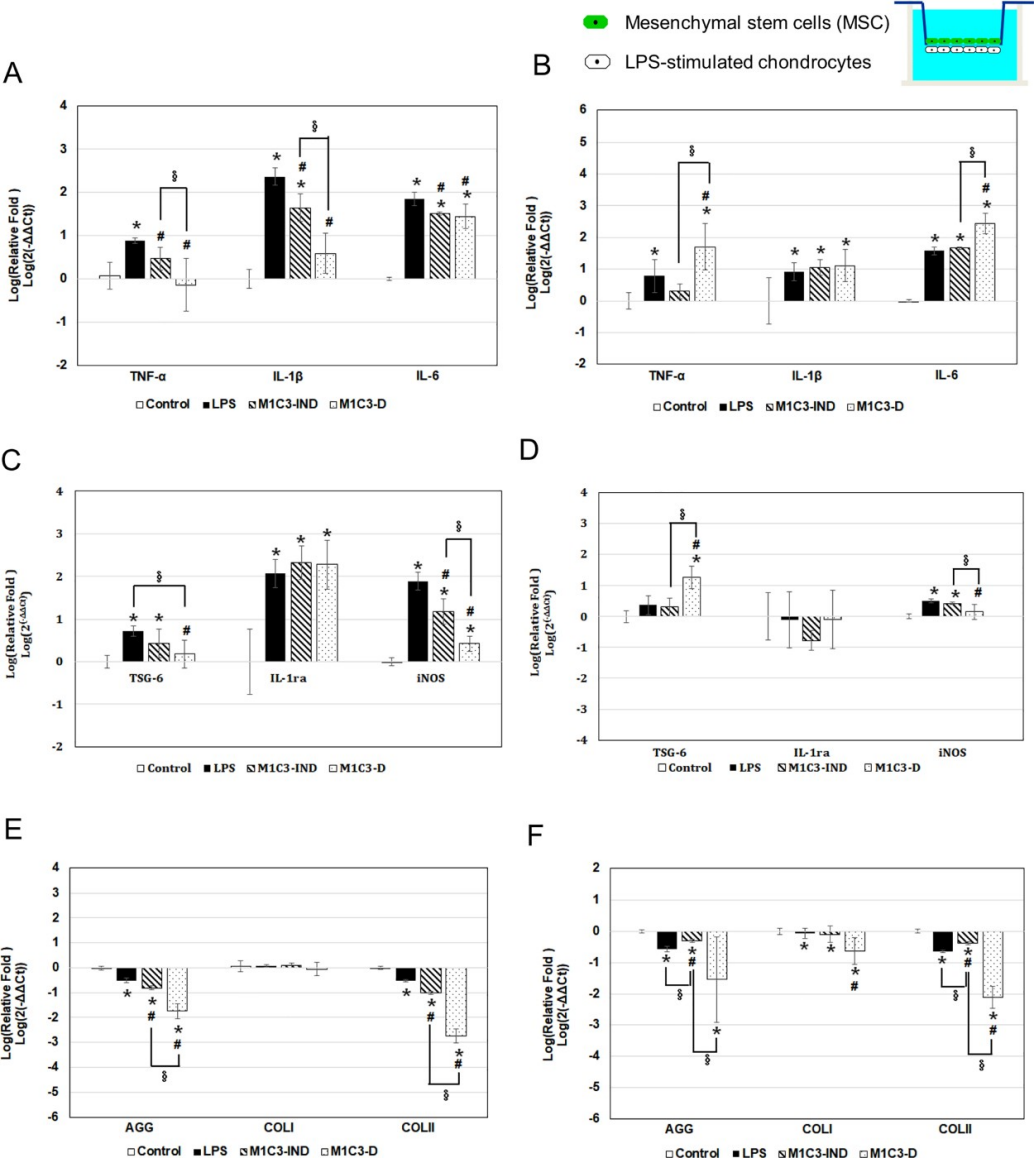
Gene expression in MSC-chondrocyte indirect and direct system in chondrocytes with LPS-induced inflammation. The setup was essentially as in Fig 4 and compared the results between indirect (M1C3-IND) and direct (M1C3-D) in a single MSCs-chondrocyte ratio of 1:3, with mRNA levels measured at 24 h (A, C, E) and 72 h (B, D, F). (A, B) are the inflammation-related genes results; (C, D) are anti-inflammation and free-radical-related genes results; (E, F) are ECM synthesis-related genes results. * *p* < 0.05, significant difference compared to the control group. ^#^ *p* < 0.05, significant difference compared to the LPS group. ^§^ *p* < 0.05, significant difference between the indicated groups.

Fig 5 shows the direct and indirect contact results comparison of chondrocyte gene expression. For MSC-chondrocyte direct contact co-culture system, chondrocytes were plated on the bottom surface of a Transwell insert, then were incubated with LPS for 4 h, then MSCs were plated on the upper surface of the insert and the cells cultured in the continued presence of LPS in a Transwell. Gene expression results of chondorcyte in indirect contact system named M1C3-IND, and the results in direct contact system named M1C3-D.

It showed that in both M1C3-IND and M1C3-D groups, the expression of inflammatory-related genes (TNF-α, IL-1β, IL-6) and free radical-related gene (iNOS) were all decreased compared with LPS group. This suggest that MSCs protect against chondrocyte inflammation through both paracrine secretion and cell-to-cell contact pathways. And the chondorcyte synthesized less TNF-α, IL-1β, TSG-6 and iNOS mRNA in the direct co-culture systems (M1C3-D) compared with those in M1C3-IND group at 24 h (Fig 5A, 5C and 5E). But this effect was decreased at 72 h, the expression of TNF-α and IL-6 mRNA of chondrocyte were increase in M1C3-D group compared with those in LPS and M1C3-IND groups. Besides, we also found the matrix synthesis-related genes AGG, COLI, and COLII were all decreased in M1C3-D group compared with those in LPS or M1C3-IND groups at 72 h (Fig 5B, 5D and 5F).

### MSC-CM system

**Fig 6:**
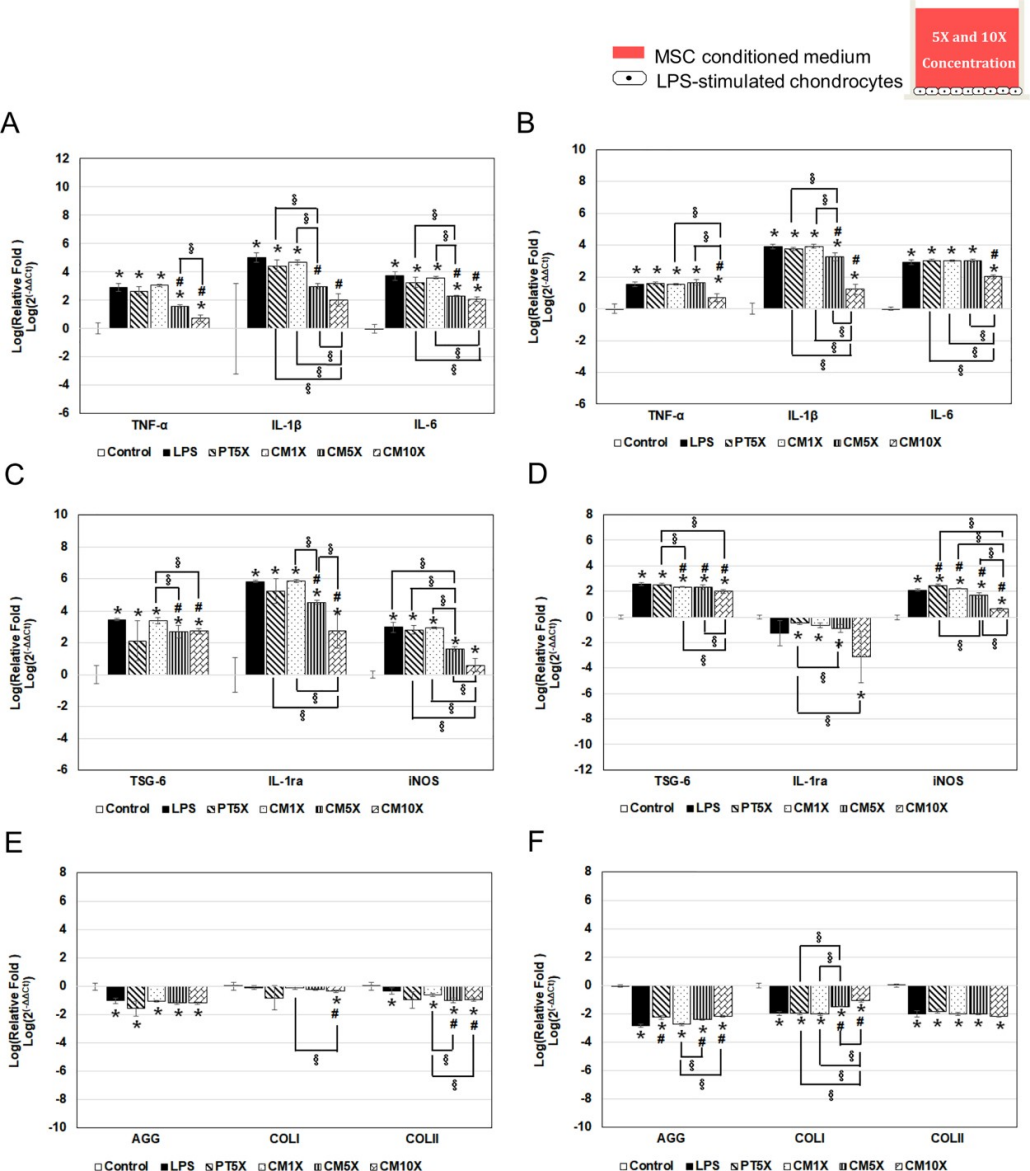
Gene expression MSC-conditioned medium in chondrocytes with LPS-induced inflammation. In the control group, chondrocytes were incubated with medium alone. In all other groups, the chondrocytes were treated with medium containing LPS (2 μg/mL) for 4 h, then the LPS-containing medium was removed and replaced with fresh medium (LPS group) or PT5X (pass-through from the 5-fold concentration of MSC-CM), CM1X (MSC-CM without concentration), CM5X (5-fold MSC-concentrated CM), or CM10X (10-fold concentrated MSC-CM) for a further 24 h (A, C, E) or 72 h (B, D, F). * p≤ 0.*05*, *significant difference compared to the Control group*. ^*#*^ *p≤ 0*.*05*, *significant difference compared to the LPS group*. ^*§*^ *p≤ 0*.*05*, *significant difference between the indicated groups*.

Fig 6 shows chondrocyte gene expression in the cytokine-only MSC-CM system. To evaluation the cytokine effect produced by MSC, chondrocytes were incubated with LPS for 4 h, then the medium was transferred to control medium (LPS group) or MSC-CM at different concentrations. Both control medium and MSC-CM do not contain FBS.

The medium of LPS group was also transferred to fresh medium after 4 h- LPS treatment, and compared with control group, the inflammation-related genes TNF-α, IL-1β, and IL-6, free radical-related gene iNOS, and anti-inflammation-related genes TSG-6, IL-1ra were upregulated in LPS group in 24 h and 72 h.

At 24 h, mRNA levels for the inflammation-related genes TNF-α, IL-1β, and IL-6, the free radical-related gene iNOS, and the anti-inflammation-related genes IL-ra in LPS-treated chondrocytes were significantly decreased by MSC-CM in a dose-response manner, with a significant decrease being seen with CM5X and CM10X MSC-CM. For ECM-related genes, chondrocyte treated with CM10X showed a downregulated expression in COLI and COLII compared with those in LPS group (Fig 6E).

After 72 h, mRNA levels for IL-1β, iNOS, and TSG-6 in chondrocytes were still downregulated in LPS-treated chondrocytes by MSC-CM in a dose-response manner, and TNF-α, IL-6, IL-1ra, iNOS expression was significantly downregulated in the CM10X group (Fig 6B and 6D). Besides, chondrocyte treated with CM10X showed an increased synthesis in AGG and COLI compared with those in LPS group (Fig 6F). We found that 10-fold concentrated CM possess the best anti-inflammation ability compared with 1X and 5X CM.

**Fig 7:**
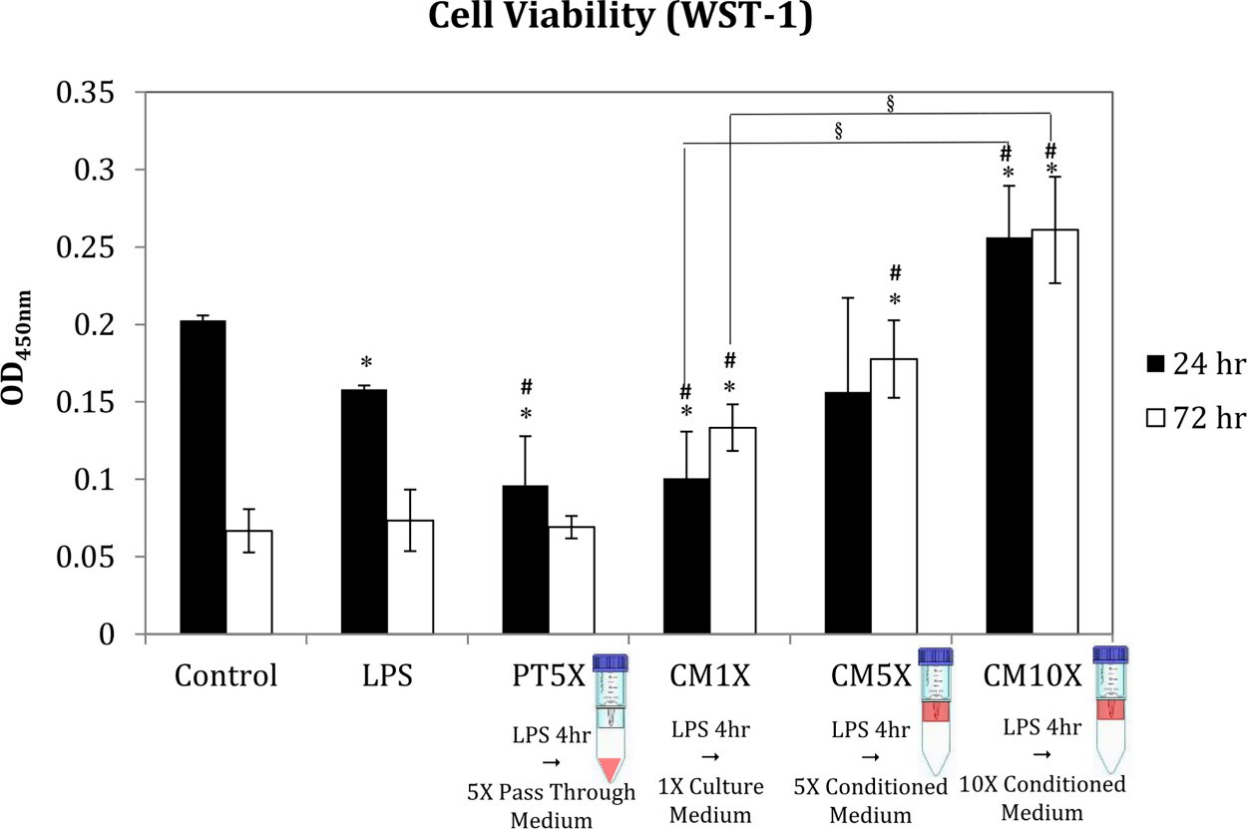
Cell viability for chondrocytes incubated with medium or LPS with or without subsequent treatment with different MSC-CM. Cells were treated as in Fig 6, then cell viability was estimated after 24 h or 72 h. * p ≤ 0.*05*, *significant difference compared to the Control group*. ^*#*^ *p ≤ 0*.*05*, *significant difference compared to the LPS group*. ^*§*^ *p ≤ 0*.*05*, *significant difference between the indicated groups*.

The effect of MSC-CM on cell viability was investigated at 24 and 72 h using the WST-1 assay. As shown in Fig 7, the cell viability of chondorcyte in control group was decreased in 72 h, this might due to the lack of FBS in the medium. LPS alone group also resulted in a significant decrease in cell viability at 24 h, but not 72 h, and this effect was greater in the PT5X-treated group. However, the cell viability in CM groups were all increase in 72 h. CM1X-treated group showed a significant decrease in cell viability compared to the control or the LPS-treated group at 24 h and a significant increase in cell numbers at 72 h. In contrast, in the CM5X-treated and CM10X-treated groups compared to the control group or LPS-treated group, there was either no significant difference (CM5X) or a significant increase (CM10X) in cell viability at 24 h and a significant increase in cell viability in both groups at 72 h, these effects being dose-dependent.

## Discussion

OA is a degenerative disease involved in many inflammatory processes [18]. In this study, we cultured chondrocytes in LPS-contained medium to mimic inflammation environment in arthritis. Ideally, using synovial fluid from OA patients to stimulate chondrocyte inflammation would be a better choice, but this method is limited by lack of repeatability [19]. LPS-stimulated inflammation can mimic the early phases of OA and offers a good alternative for studying chondrocyte changes in OA [7, 8]. LPS treatment was found to significantly increase TNF-α, IL-1β, IL-6, and iNOS mRNA levels in chondrocytes without inducing cell death (Figs 2 and 3).

Clinically, hyaluronic acid (HA) injection is one of the major methods for early OA treatment, but it can only temporary relief the pain through the lubrication function. Several anti-cytokine drugs, such as anakinra and infliximab, have been examined for treatment of OA in animal studies or in clinical trials [20, 21]. These anti-inflammatory drugs may have some limitations, such as an increased risk of infection (e.g., tuberculosis) [22] and the possibility of developing autoimmune disorders [23]. Thus, to find a safe and effective method for OA therapy is needed.

MSCs injection is one of the promising cell therapy methods since it possesses self-renewal and multipotent differentiation ability. Besides, it can be isolated from patients themselves, and they do not have rejection problems. Importantly, they play an important role in cartilage tissue repair [24–26] and anti-inflammation therapy for OA [27–30].

To examine the anti-inflammation ability of MSC and to discuss the anti-inflammation mechanism of it, first, we used an indirect contact system to evaluate the paracrine effect between MSC and chondrocyte. We cultured chondrocytes on the bottom of the outer chamber of a Transwell and MSCs on top of the insert (Fig 1A, model I) to study the release of inflammation-related factors from LPS-stimulated chondrocytes. Cristina Manferdini’s research group have ever done similar research by using 19 total knee replacement patient’s cartilage tissue and co-cultured them with adipose-derived MSC (AD-MSC) in 2013 [28]. They found all three different kinds of AD-MSC (from infrapatellar Hoffa fat, subcutaneous hip fat and subcutaneous abdominal fat) could reduce the expression of IL-1β, IL-6, and CXCL8/IL-8 level on chondrocyte. Rilong Jin’s group also found human AD-MSC could down-regulate the amount of NO, PGE-2 and MMP-3, and decrease IL-6 gene expression of chondrocyte pre-treated with IL-1β [29]. Li et al. found AD-MSCs could down-upregulation of MMP-3, MMP-13, TNF-α and IL-6 in chondrocytes [30]. In the present study, we focus on bone marrow MSC, and evaluated the inflammation and ECM synthesis related gene expression of LPS-treated chondrocyte. Results showed that in the MSC-chondrocyte indirect contact system, the expression of inflammation-related genes (TNF-α, IL-1β, and IL-6), one free radical-related gene (iNOS), and one ECM synthesis-related gene (AGG) was downregulated in LPS-stimulated chondrocytes (Fig 4) which is similar to that in Rilong Jin’s, Li’s and Cristina Manferdini’s research. And the optimal MSC/chondrocyte ratios for inflammatory inhibition is 1:1 (M1C1) or 1:3 (M1C3).

In addition, we used a direct contact co-culture system to study the effect of both cell-cell contact and exchange of soluble mediators; in this system, inflammatory chondrocytes and MSCs were cultured on opposite sides of the Transwell insert (Fig 1A II). The pore size of the Transwell is 0.4μm. The cells can cross-talk by filaments passing through the small holes of the membrane [31]. This system has been used in studies to investigate cell-cell signaling and potential clinical application [15, 16]. Watanabe et al. [16] used the direct contact co-culture system to study human nucleus pulposus (NP) cells and MSCs, and found that MSCs enhance the biological properties and increase numbers of human NP cells. In the present MSC-chondrocyte indirect (M1C3-IND) and direct (M1C3-D) contact system comparison, we found that the anti-inflammation ability of MSC was better in M1C3-D at 24 h (TNF-α, IL-1β, iNOS ↓), but this effect was decreased at 72 h. The expression of TNF-α, IL-6, and iNOS mRNA of chondrocyte were increase in M1C3-D group compared with those in LPS and M1C3-IND groups. Besides, an decreased in AGG and COLII mRNA were also found in M1C3-D group at 72 h. This means the direct cell-cell contact would influence the inflammation status of chondrocyte. It can inhibit the inflammation of chondrocyte in short period of time (24 h), but the direct contact between chondrocyte and MSC for about 72 h may decrease the anti-inflammation ability of MSC, and lead to the increase in inflammation-related genes of chondrocyte. Moreover, we also found there is less ECM related gene mRNA (AGG, COLII) have been synthesized in M1C3-D group compared with those in M1C3-IND group.

Then, we studied the effect of MSCs soluble mediators by adding MSC-CM to LPS-stimulated chondrocyte. In this system, chondrocytes plated on the bottom of the outer chamber were incubated with LPS for 4 h, then the medium was removed and replaced with 2 ml of CM from MSCs for 24 or 72 h (Fig 1B) to evaluate the anti-inflammatory activity of soluble mediators produced by MSCs [32]. In LPS group, the medium removal was also conducted on chondrocyte after 4 h- LPS treatment and replaced with 2 ml of fresh medium. G.M. van Buul et al found the expression of IL-1ra was upregulated and collagen type II were downregulated in cartilage explants under MSC-conditioned medium treatment [32]. In Julia Platas’s research, they proved CM from AD-MSC could enhanced IL-10 levels in OA chondrocytes, and decreasing the levels of MMP-3 and MMP-13 mRNA in OA chondrocytes stimulated with IL-1𝛽 [33]. However, in C. Manferdini et al, they found an opposite result, they showed that CM from AD-MSC cannot decrease a series of inflammation related cytokine including IL-6, CCL2/MCP-1, CCL3/MIP1-a and CCL5/RANTES [28]. Our findings are similar to the results of G.M. van Buul’s and Julia Platas’s research. MSC-CM could significantly downregulate the expression of inflammation-related genes (TNF-α, IL-1β, and IL-6), one free radical-related gene (iNOS), and upregulated ECM-synthesis-related gene (AGG) in LPS-stimulated chondrocytes in a dose-dependent manner. Besides, in contrast to the results in the previous models, treatment with MSC-CM for a longer period (72 h) did not result in upregulation of the expression of inflammation-related genes, especially in the group of CM10X (Fig 6).

Even though there are several studies discuss the anti-inflammation ability of MSC through MSC-chondrocyte coculture system and CM system [32–34], to the best of our knowledge, this is the first study comparing the results between indirect and direct cell contact systems under the same treatment condition including cell seeding number and LPS concentration. Based on our results, we found the anti-inflammation ability of MSC in indirect contact system is better than that in direct contact system, though in the direct contact system, the MSC and chondrocyte can conduct signal modulation through both paracrine secretion and cell-cell contact methods. On the other hand, we also evaluated the cytokine effect by MSC conditioned medium system, all the medium of LPS and CM groups were replaced with fresh medium (for LPS group) or conditioned medium (for CM groups) after 4 h-LPS treatment. Results showed that MSC-CM downregulate chondrocytes inflammation even for 72 h cultivation, especially in high CM concentration group (eg. CM10X). Thus, we spectated this might due to the high concentration of cytokines in MSC-CM assist chondrocyte recovery from LPS-stimulation. Various major proliferative factors, including TGF-β, vascular endothelial growth factor, hepatocyte growth factor, placental growth factor, and insulin-like growth factor, which may enhance cell growth and proliferation and attenuate cell death, are present in MSC-CM [35]. MSC-CM also enhances the survival and proliferation of, and decreases apoptosis of, chemically-injured corneal epithelial progenitor cells [36]. The MSC-CM might enhance chondrocyte recovery by affecting chondrocyte viability in our experimental model. In the present study, MSC-CM increased LPS-treated chondrocyte numbers at 72 h to levels even significantly higher than those in the control group not incubated with LPS (Fig 7). It is likely that MSC-CM enhances chondrocyte cell viability and attenuates cell death, although more studies are needed to confirm this hypothesis, and identified the key component in the CM.

Some considerations suggest that MSC-CM may have great clinical potential for OA therapy. First, it can be manufactured under optimized conditions *in vitro* for mass production. Second, it can be freeze-dried, packaged, and transported more easily than MSCs. Thirdly, a single preparation of CM can be used to perform several injections of OA joints and there is no need to culture MSCs before each injection. Last, but not least, there might be less problem of rejection, as a donor’s MSC-CM can be used in many recipients [37]. In summary, MSC-CM has multiple beneficial effects on inflammatory chondrocytes, including an anti-inflammatory effect and an increase in chondrocyte numbers. It is therefore important to pursue this avenue for OA treatment.

One limitation of this study is the use of an ex vivo cell model, rather than in vivo animal studies. Future studies will help in designing clinical trials of MSC-based OA therapy by evaluating the therapeutic effect of autologous or even allogeneic MSCs in an OA animal model. To sum up, our data showed the anti-inflammation ability of MSC may influence by chondrocyte through both paracrine secretion and cell-to-cell contact pathways, a decreased anti-inflammation of MSC was found in the MSC-chondrocyte direct contact system after incubation for 72 h.

On the other side, the CM prepared by DMEM without FBS incubation with MSC may serve as another choice for OA injection, current results also showed that only 10-fold concentrated CM could downregulate chondrocytes inflammation for 72 h cultivation.

## Conclusions

This study demonstrates that MSCs can exert an anti-inflammatory effect on chondrocytes through both a paracrine effect and cell-cell contact signaling. But the anti-inflammation ability of MSC would decrease with incubation time increase, especially in direct cell contact co-culture system. Considering using cytokine for OA treatment, MSC-CM could be another choice for OA therapy, it had a dose dependent anti-inflammatory capability, and the 10-fold concentrated CM possess the best treatment effect compared with that in 1X and 5X CM.

## Supporting information

S1 Data Data of Fig 2 effects of lipopolysaccharide (LPS) on inflammatory gene expressions on chondrocytes. (PDF)

S2 Data Data of Fig 3 effects of LPS on chondrocyte morphology and cell numbers. (PDF)

S3 Data Data of Fig 4 gene expression in MSC-chondrocyte indirect system in chondrocytes with LPS-induced inflammation at 24 hr. (PDF)

S4 Data Data of Fig 4 gene expression in MSC-chondrocyte indirect system in chondrocytes with LPS-induced inflammation at 72 hr. (PDF)

S5 Data Data of Fig 5 gene expression in MSC-chondrocyte indirect and direct system in chondrocytes with LPS-induced inflammation at 24 hr. (PDF)

S6 Data Data of Fig 5 gene expression in MSC-chondrocyte indirect and direct system in chondrocytes with LPS-induced inflammation at 72 hr. (PDF)

S7 Data Data of Fig 6 gene expression MSC-conditioned medium in chondrocytes with LPS-induced inflammation at 24 hr. (PDF)

S8 Data Data of Fig 6 gene expression MSC-conditioned medium in chondrocytes with LPS-induced inflammation at 72 hr. (PDF)

S9 Data Data of Fig 7 cell viability for chondrocytes incubated with medium or LPS with or without subsequent treatment with different MSC-CM. (PDF)
