# Supplementary material for: Can mesenchymal stem cells and their conditioned medium assist inflammatory chondrocytes recovery?
Source: PLoS One. 2018 Nov 21;13(11):e0205563. doi: 10.1371/journal.pone.0205563 (PMC6248915; doi:10.1371/journal.pone.0205563)

Figure 2. Effects of lipopolysaccharide (LPS) on inflammatory gene expressions on chondrocytes.
Evaluation time: 4 hours

Ct number

LPS concentration (μg/mL)	TNF-α	IL-1β	IL-6	iNOS	GAPDH
0	30.99	42.43	29.84	36.30	38.82
0	30.13	40.37	27.97	35.39	33.85
0	31.17	40.72	28.36	36.48	37.38
2	24.89	30.64	23.79	26.50	38.50
2	24.45	30.87	24.26	25.59	35.23
2	25.19	31.08	22.72	26.55	39.80
20	25.84	31.87	22.23	27.13	39.11
20	25.06	30.44	21.99	26.45	34.68
20	25.52	30.81	21.69	26.64	35.13
200	24.91	29.97	21.06	26.26	38.98
200	25.09	29.53	22.24	26.72	35.33
200	25.78	30.03	20.82	27.36	37.46

Step.1
ΔCt number (=Target gene Ct- GAPDH Ct)

LPS concentration (μg/mL)	TNF-α	IL-1β	IL-6	iNOS
0	-7.8	3.6	-9.0	-2.5
0	-3.7	6.5	-5.9	1.5
0	-6.2	3.3	-9.0	-0.9
2	-13.6	-7.9	-14.7	-12.0
2	-10.8	-4.4	-11.0	-9.6
2	-14.6	-8.7	-17.1	-13.3
20	-13.3	-7.2	-16.9	-12.0
20	-9.6	-4.2	-12.7	-8.2
20	-9.6	-4.3	-13.4	-8.5
200	-14.1	-9.0	-17.9	-12.7
200	-10.2	-5.8	-13.1	-8.6
200	-11.7	-7.4	-16.6	-10.1

Step. 2
ΔΔCt (=Experimal ΔCt- Control ΔCt)

LPS concentration (μg/mL)	TNF-α	IL-1β	IL-6	iNOS
0	-1.91	-0.88	-1.02722	-1.89792
0	2.20	2.03	2.07392	2.16477
0	-0.29	-1.15	-1.05461	-0.2656
2	-7.69	-12.35	-6.75614	-11.3732
2	-4.87	-8.85	-3.01235	-9.01075
2	-8.70	-13.21	-9.12248	-12.6234
20	-7.35	-11.73	-8.91473	-11.3502
20	-3.70	-8.73	-4.72771	-7.59729
20	-3.69	-8.81	-5.47756	-7.86119
200	-8.15	-13.50	-9.96456	-12.091
200	-4.33	-10.29	-5.13011	-7.9792
200	-5.75	-11.92	-8.67578	-9.46916

Control ΔCt

LPS concentration (μg/mL)	TNF-α	IL-1β	IL-6	iNOS
0	-7.83	3.61	-8.98722	-2.52792
0	-3.72	6.52	-5.88608	1.53477
0	-6.21	3.34	-9.01461	-0.8956
Avg.	-5.92	4.49	-7.96	-0.63
std.	2.073561	1.762141	1.798403	2.044366

Step. 3
Relative Fold (= 2^(-ΔΔCt))

LPS concentration (μg/mL)	TNF-α	IL-1β	IL-6	iNOS
0	3.770704	1.846756	2.038091	3.726748
0	0.217302	0.245283	0.237513	0.223018
0	1.220898	2.21505	2.077161	1.20214
2	206.0001	5225.254	108.0939	2652.561
2	29.22593	462.9458	8.0688	515.8281
2	414.6438	9490.217	557.3637	6309.903
20	163.372	3395.535	482.6162	2610.658

Step. 4
Log(Relative Fold (= 2^(-ΔΔCt)))

LPS concentration (μg/mL)	TNF-α	IL-1β	IL-6	iNOS
0	0.576422	0.26641	0.309224	0.57133
0	-0.66294	-0.61033	-0.62431	-0.65166
0	0.086679	0.345384	0.31747	0.079955
2	2.313868	3.718107	2.033801	3.423665
2	1.465768	2.66553	0.906809	2.712505
2	2.617675	3.977276	2.746139	3.800023
20	2.213178	3.530908	2.683602	3.41675
20	1.112942	2.6266	1.423183	2.287012
20	1.110288	2.652731	1.648909	2.366455
200	2.454678	4.064	2.99963	3.639767
200	1.302024	3.098025	1.544317	2.401978
200	1.732347	3.586888	2.61167	2.850502

Step. 5
Ave.

20	12.97007	423.2527	26.49619	193.6474
20	12.89105	449.5018	44.55629	232.5173
200	284.8908	11587.76	999.1489	4362.82
200	20.04581	1253.213	35.02006	252.3354
200	53.99422	3862.675	408.9497	708.7643

	TNF-α	IL-1β	IL-6	iNOS
Control	5.5E-05	0.00049	0.00079	-0.0001
2 μg/mL	2.13244	3.45364	1.89558	3.31206
20 μg/mL	1.4788	2.93675	1.91856	2.69007
200 μg/mL	1.82968	3.58297	2.38521	2.96408

std.

	TNF-α	IL-1β	IL-6	iNOS
Control	0.6242	0.53046	0.54137	0.61542
2 μg/mL	0.597	0.69471	0.92742	0.55228
20 μg/mL	0.63599	0.51472	0.67209	0.63057
200 μg/mL	0.58246	0.483	0.75362	0.62666

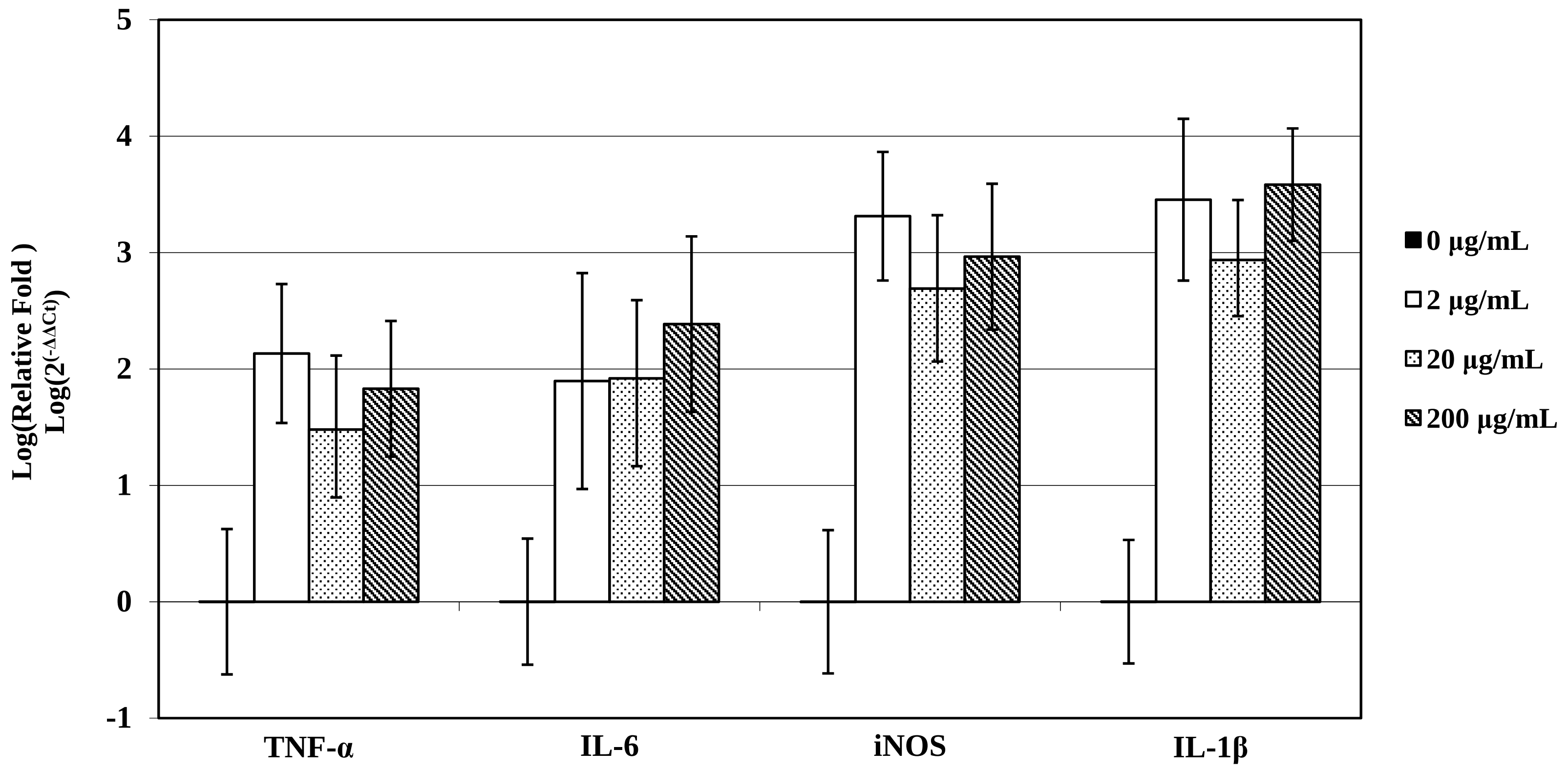

Supplement: S1 Data — (PDF) [file pone.0205563.s001.pdf]
