# Supplementary material for: Can mesenchymal stem cells and their conditioned medium assist inflammatory chondrocytes recovery?
Source: PLoS One. 2018 Nov 21;13(11):e0205563. doi: 10.1371/journal.pone.0205563 (PMC6248915; doi:10.1371/journal.pone.0205563)

Figure 3. Effects of LPS on chondrocyte morphology and cell numbers.
Evaluation Time Point: 24hr., 72 hr.

24 hr LPS treatment	OD450nm					Ave.	Ave.-blan	Std.
Control	0.654	0.732	0.711	0.73	0.656	0.6966	0.2991	0.02834
2μg/ml LPS	0.722	0.696	0.726	0.703	0.696	0.7086	0.3111	0.0141
20μg/ml LPS	0.719	0.7	0.696	0.681	0.709	0.701	0.3035	0.07139
200μg/ml LPS	0.509	0.643	0.666	0.541	0.653	0.6024	0.2049	0.11668
Negative Control	0.396	0.406	0.409	0.399	0.394	0.4008	0.0033	0.00495
blank	0.396	0.398	0.399	0.397		0.3975		

p.s Negative Control = 1% triton

72 hr LPS treatment	OD450nm					Ave.	Ave.-blan	Std.
Control	2.215	2.006	2.095	2.121	2.077	2.1028	1.6478	0.07586
2μg/ml LPS	2.677	2.353	2.432	2.325	2.407	2.4388	1.9838	0.13974
20μg/ml LPS	2.599	2.21	2.27	2.223	2.345	2.3294	1.8744	0.15971
200μg/ml LPS	3.015	2.877	2.861	2.821	2.777	2.8702	2.4152	0.08973
Negative Control	0.386	0.398	0.651	0.416	0.397	0.4496	-0.0054	0.1131
blank	0.439		0.465	0.461	0.456	0.45525		

p.s Negative Control = 1% triton

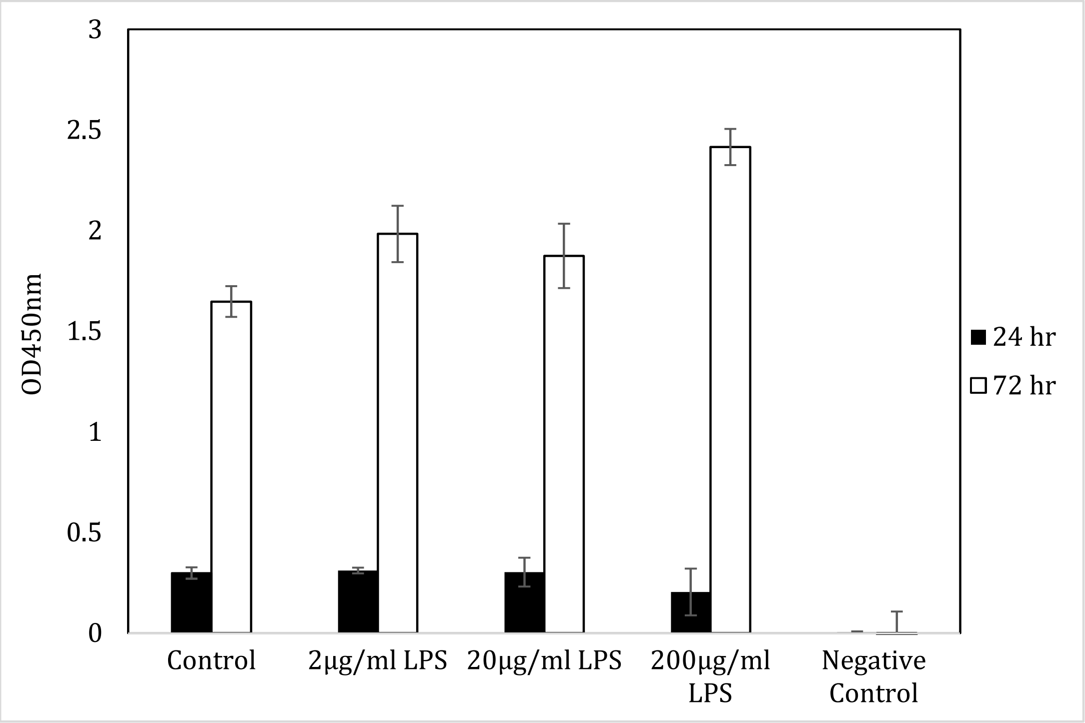

Supplement: S2 Data — (PDF) [file pone.0205563.s002.pdf]
