# Supplementary material for: Can mesenchymal stem cells and their conditioned medium assist inflammatory chondrocytes recovery?
Source: PLoS One. 2018 Nov 21;13(11):e0205563. doi: 10.1371/journal.pone.0205563 (PMC6248915; doi:10.1371/journal.pone.0205563)
Supplement: S3 Data — (PDF) [file pone.0205563.s003.pdf]

Figure 4. Gene expression in MSC-chondrocyte indirect system in chondrocytes with LPS-induced inflammation.
Evaluation Time Point: 24 hr.
Indirect System

Ct number

	TNF-α	IL-1β	IL-6	iNOS	AGG	COLII	GAPDH
Control	27.83	32.72	26.83	30.95	19.56	21.77	33.48
Control	26.88	32.97	26.73	30.8	19.69	21.72	33.25
Control	28.75	33.71	26.95	31.41	19.8	21.89	33.4
Control	27.46	33.13	27.09	30.81	19.78	21.85	33.64
Control	27.49	34.19	26.31	30.52	19.84	21.74	33.13
LPS	25.33	26.25	20.66	25.55	21.58	23.64	33.64
LPS	24.57	25.35	19.94	24.58	21.69	23.67	33.26
LPS	24.52	24.48	21.25	23.78	21.6	23.67	33.35
LPS	24.69	26.35	20.58	25.34	21.28	23.53	33.51
LPS	25.12	25.45	21.25	24.33	21.45	23.69	33.62
M1C1	25.86	26.46	21.99	30.62	21.63	23.75	33.6
M1C1	24.11	27.46	20.87	26.17	20.84	23.47	32.12
M1C1	24.23	26.52	20.64	25.82	20.88	23.84	31.98
M1C1	26.31	26.98	20.77	25.88	21.05	23.66	32.06
M1C1	25.12	25.74	20.47	29.35	21.54	23.68	32.15
M1C3	25.23	27.26	20.45	26.65	20.97	23.65	32.12
M1C3	25.53	25.66	20.33	24.76	20.87	23.6	32.09
M1C3	25.52	26.03	20.49	25.04	20.84	23.64	31.91
M1C3	23.85	27.84	20.26	26.66	21.07	23.75	31.84
M1C3	23.55	25.54	20.32	24.75	21.37	23.94	31.94
M1C5	23.44	27.88	19.63	26.8	20.93	23.61	31.73
M1C5	23.69	26.26	20.14	25.01	20.76	23.49	31.95
M1C5	24.99	25.31	19.92	24.5	20.96	23.7	31.97
M1C5	23.58	26.91	19.98	26.59	21.06	23.78	32.37
M1C5	26.85	27.8	20.97	27.25	22.24	24.94	33.08

	TSG-6	IL-1ra	Col I	GAPDH
Control	33.7	39.18	12.34	32.29
Control	33.18	43.5	11.81	32.76
Control	34.11		11.49	32.79
Control	35.04	41.96	11.74	33.26
Control	33.62	45.54	11.59	32.71
LPS	31.11	34.6	11.34	32.83
LPS	31.3	36.55	11.44	32.8
LPS	31.93	34.81	11.53	32.71
LPS	31.65	35.36	11.58	32.7
LPS	31.8	37.19	11.67	32.84
M1C1	32.7	41.78	11.67	32.91
M1C1	33.39	41.94	11.54	33.09
M1C1	32.29	38.45	11.49	32.85
M1C1	31.75	35.68	11.2	32.87
M1C1	31.39	36	12.05	32.89
M1C3	32.43		11.28	32.91
M1C3	31.56	33.95	11.6	33.23
M1C3	31.83	33.94	11.7	32.74
M1C3	33.75	36.13	11.98	32.98
M1C3	33.69	35.97	11.74	32.77
M1C5	31.38	39.2	12.2	33.22
M1C5	30.77	35.79	11.68	31.48
M1C5	30.32	35.32	12.08	33.26
M1C5	32.18		12.07	33.23
M1C5	33.9	43.53	13.72	34.45

Step.1

ΔCt number (=Target gene Ct- GAPDH Ct)

	TNF-α	IL-1β	IL-6	TSG-6	IL-1ra	iNOS	AGG	COLI	COLII
Control	-5.65	-0.76	-6.65	1.41	6.89	-2.53	-13.92	-19.95	-11.71
Control	-6.37	-0.28	-6.52	0.42	10.74	-2.45	-13.56	-20.95	-11.53
Control	-4.65	0.31	-6.45	1.32		-1.99	-13.6	-21.3	-11.51
Control	-6.18	-0.51	-6.55	1.78	8.7	-2.83	-13.86	-21.52	-11.79
Control	-5.64	1.06	-6.82	0.91	12.83	-2.61	-13.29	-21.12	-11.39
LPS	-8.31	-7.39	-12.98	-1.72	1.77	-8.09	-12.06	-21.49	-10
LPS	-8.69	-7.91	-13.32	-1.5	3.75	-8.68	-11.57	-21.36	-9.59
LPS	-8.83	-8.87	-12.1	-0.78	2.1	-9.57	-11.75	-21.18	-9.68
LPS	-8.82	-7.16	-12.93	-1.05	2.66	-8.17	-12.23	-21.12	-9.98
LPS	-8.5	-8.17	-12.37	-1.04	4.35	-9.29	-12.17	-21.17	-9.93
M1C1	-7.74	-7.14	-11.61	-0.21	8.87	-2.98	-11.97	-21.24	-9.85
M1C1	-8.01	-4.66	-11.25	0.3	8.85	-5.95	-11.28	-21.55	-8.65
M1C1	-7.75	-5.46	-11.34	-0.56	5.6	-6.16	-11.1	-21.36	-8.14
M1C1	-5.75	-5.08	-11.29	-1.12	2.81	-6.18	-11.01	-21.67	-8.4

Control ΔCt

	TNF-α	IL-1β	IL-6	TSG-6	IL-1ra	iNOS	AGG	COLI	COLII
	-5.65	-0.76	-6.65	1.41	6.89	-2.53	-13.92	-19.95	-11.71
	-6.37	-0.28	-6.52	0.42	10.74	-2.45	-13.56	-20.95	-11.53
	-4.65	0.31	-6.45	1.32		-1.99	-13.6	-21.3	-11.51
	-6.18	-0.51	-6.55	1.78	8.7	-2.83	-13.86	-21.52	-11.79
	-5.64	1.06	-6.82	0.91	12.83	-2.61	-13.29	-21.12	-11.39
Ave.	-5.698	-0.036	-6.598	1.168	9.79	-2.482	-13.646	-20.968	-11.586
std.	0.59798	0.65258	0.12828	0.46525	2.22161	0.27672	0.22677	0.54308	0.14444

Step. 4

Log(Relative Fold (= 2^(-ΔΔCt)))

	TNF-α	IL-1β	IL-6	TSG-6	IL-1ra	iNOS	AGG	COLI	COLII
Control	-0.0144	0.21795	0.01565	-0.0728	0.87299	0.01445	0.08248	-0.3064	0.03733
Control	0.20229	0.07345	-0.0235	0.22517	-0.286	-0.0096	-0.0259	-0.0054	-0.0169
Control	-0.3155	-0.1042	-0.0446	-0.0458		-0.1481	-0.0138	0.09994	-0.0229

M1C1	-7.03	-6.41	-11.68	-1.5	3.11	-2.8	-10.61	-20.84	-8.47
M1C3	-6.89	-4.86	-11.67	-0.48		-5.47	-11.15	-21.63	-8.47
M1C3	-6.56	-6.43	-11.79	-1.67	0.72	-7.33	-11.22	-21.63	-8.49
M1C3	-6.39	-5.88	-11.42	-0.91	1.2	-6.87	-11.07	-21.04	-8.27
M1C3	-7.99	-4	-11.58	0.77	3.15	-5.18	-10.77	-21	-8.09
M1C3	-8.39	-6.4	-11.62	0.92	3.2	-7.19	-10.57	-21.03	-8
M1C5	-8.29	-3.85	-12.1	-1.84	5.98	-4.93	-10.8	-21.02	-8.12
M1C5	-8.26	-5.69	-11.81	-0.71	4.31	-6.94	-11.19	-19.8	-8.46
M1C5	-6.98	-6.66	-12.05	-2.94	2.06	-7.47	-11.01	-21.18	-8.27
M1C5	-8.79	-5.46	-12.39	-1.05		-5.78	-11.31	-21.16	-8.59
M1C5	-6.23	-5.28	-12.11	-0.55	9.08	-5.83	-10.84	-20.73	-8.14

Step. 2

ΔΔCt (=Experimal ΔCt- Control ΔCt)

	TNF-α	IL-1β	IL-6	TSG-6	IL-1ra	iNOS	AGG	COLI	COLII
Control	0.048	-0.724	-0.052	0.242	-2.9	-0.048	-0.274	1.018	-0.124
Control	-0.672	-0.244	0.078	-0.748	0.95	0.032	0.086	0.018	0.056
Control	1.048	0.346	0.148	0.152		0.492	0.046	-0.332	0.076
Control	-0.482	-0.474	0.048	0.612	-1.09	-0.348	-0.214	-0.552	-0.204
Control	0.058	1.096	-0.222	-0.258	3.04	-0.128	0.356	-0.152	0.196
LPS	-2.612	-7.354	-6.382	-2.888	-8.02	-5.608	1.586	-0.522	1.586
LPS	-2.992	-7.874	-6.722	-2.668	-6.04	-6.198	2.076	-0.392	1.996
LPS	-3.132	-8.834	-5.502	-1.948	-7.69	-7.088	1.896	-0.212	1.906
LPS	-3.122	-7.124	-6.332	-2.218	-7.13	-5.688	1.416	-0.152	1.606
LPS	-2.802	-8.134	-5.772	-2.208	-5.44	-6.808	1.476	-0.202	1.656
M1C1	-2.042	-7.104	-5.012	-1.378	-0.92	-0.498	1.676	-0.272	1.736
M1C1	-2.312	-4.624	-4.652	-0.868	-0.94	-3.468	2.366	-0.582	2.936
M1C1	-2.052	-5.424	-4.742	-1.728	-4.19	-3.678	2.546	-0.392	3.446
M1C1	-0.052	-5.044	-4.692	-2.288	-6.98	-3.698	2.636	-0.702	3.186
M1C1	-1.332	-6.374	-5.082	-2.668	-6.68	-0.318	3.036	0.128	3.116
M1C3	-1.192	-4.824	-5.072	-1.648		-2.988	2.496	-0.662	3.116
M1C3	-0.862	-6.394	-5.192	-2.838	-9.07	-4.848	2.426	-0.662	3.096
M1C3	-0.692	-5.844	-4.822	-2.078	-8.59	-4.388	2.576	-0.072	3.316
M1C3	-2.292	-3.964	-4.982	-0.398	-6.64	-2.698	2.876	-0.032	3.496
M1C3	-2.692	-6.364	-5.022	-0.248	-6.59	-4.708	3.076	-0.062	3.586
M1C5	-2.592	-3.814	-5.502	-3.008	-3.81	-2.448	2.846	-0.052	3.466
M1C5	-2.562	-5.654	-5.212	-1.878	-5.48	-4.458	2.456	1.168	3.126
M1C5	-1.282	-6.624	-5.452	-4.108	-7.73	-4.988	2.636	-0.212	3.316
M1C5	-3.092	-5.424	-5.792	-2.218		-3.298	2.336	-0.192	2.996
M1C5	-0.532	-5.244	-5.512	-1.718	-0.71	-3.348	2.806	0.238	3.446

Step. 3

Relative Fold (= 2^(-ΔΔCt))

	TNF-α	IL-1β	IL-6	TSG-6	IL-1ra	iNOS	AGG	COLI	COLII
Control	0.96728	1.65176	1.0367	0.84557	7.46426	1.03383	1.20916	0.4938	1.08975
Control	1.59328	1.18427	0.94737	1.67946	0.51763	0.97806	0.94213	0.9876	0.96193
Control	0.48364	0.78676	0.9025	0.9		0.71104	0.96862	1.25876	0.94868
Control	1.39668	1.38896	0.96728	0.65429	2.12874	1.27279	1.1599	1.46612	1.15189
Control	0.96059	0.46781	1.16635	1.19582	0.12158	1.09278	0.78133	1.11111	0.87297
LPS	6.11351	163.597	83.4014	7.40244	259.574	48.7726	0.33309	1.43594	0.33309

Control	0.1451	0.14269	-0.0144	-0.1842	0.32812	0.10476	0.06442	0.16617	0.06141
Control	-0.0175	-0.3299	0.06683	0.07767	-0.9151	0.03853	-0.1072	0.04576	-0.059
LPS	0.78629	2.21377	1.92117	0.86937	2.41426	1.68818	-0.4774	0.15714	-0.4774
LPS	0.90068	2.37031	2.02352	0.80315	1.81822	1.86578	-0.6249	0.118	-0.6009
LPS	0.94283	2.6593	1.65627	0.58641	2.31492	2.1337	-0.5708	0.06382	-0.5738
LPS	0.93982	2.14454	1.90612	0.66768	2.14634	1.71226	-0.4263	0.04576	-0.4835
LPS	0.84349	2.44858	1.73755	0.66467	1.6376	2.04941	-0.4443	0.06081	-0.4985
M1C1	0.6147	2.13852	1.50876	0.41482	0.27695	0.14991	-0.5045	0.08188	-0.5226
M1C1	0.69598	1.39196	1.40039	0.26129	0.28297	1.04397	-0.7122	0.1752	-0.8838
M1C1	0.61771	1.63279	1.42748	0.52018	1.26132	1.10719	-0.7664	0.118	-1.0373
M1C1	0.01565	1.5184	1.41243	0.68876	2.10119	1.11321	-0.7935	0.21132	-0.9591
M1C1	0.40097	1.91877	1.52983	0.80315	2.01088	0.09573	-0.9139	-0.0385	-0.938
M1C3	0.35883	1.45217	1.52682	0.4961		0.89948	-0.7514	0.19928	-0.938
M1C3	0.25949	1.92479	1.56295	0.85432	2.73034	1.45939	-0.7303	0.19928	-0.932
M1C3	0.20831	1.75922	1.45157	0.62554	2.58585	1.32092	-0.7755	0.02167	-0.9982
M1C3	0.68996	1.19328	1.49973	0.11981	1.99884	0.81218	-0.8658	0.00963	-1.0524
M1C3	0.81037	1.91575	1.51177	0.07466	1.98379	1.41725	-0.926	0.01866	-1.0795
M1C5	0.78027	1.14813	1.65627	0.9055	1.14692	0.73692	-0.8567	0.01565	-1.0434
M1C5	0.77124	1.70202	1.56897	0.56533	1.64964	1.34199	-0.7393	-0.3516	-0.941
M1C5	0.38592	1.99402	1.64122	1.23663	2.32696	1.50154	-0.7935	0.06382	-0.9982
M1C5	0.93078	1.63279	1.74357	0.66768		0.9928	-0.7032	0.0578	-0.9019
M1C5	0.16015	1.5786	1.65928	0.51717	0.21373	1.00785	-0.8447	-0.0716	-1.0373

Step. 5

Ave.

	TNF-α	IL-1β	IL-6	TSG-6	IL-1ra	iNOS	AGG	COLI	COLII
Control	3.5E-17	0	-1E-16	0	0	0	-4E-16	4.5E-16	-7E-16
LPS	0.88262	2.3673	1.84893	0.71826	2.06627	1.88987	-0.5087	0.0891	-0.5268
M1C1	0.469	1.72009	1.45578	0.53764	1.18666	0.702	-0.7381	0.10957	-0.8682
M1C3	0.46539	1.64904	1.51057	0.43409	2.3247	1.18184	-0.8098	0.08971	-1
M1C5	0.60567	1.61111	1.65386	0.77846	1.33432	1.11622	-0.7875	-0.0572	-0.9844

std.

	TNF-α	IL-1β	IL-6	TSG-6	IL-1ra	iNOS	AGG	COLI	COLII
No LPS	0.20126	0.21963	0.04317	0.15658	0.77223	0.09313	0.07632	0.18278	0.04861
LPS	0.06714	0.20315	0.14874	0.11495	0.32963	0.19857	0.08556	0.04686	0.05658
M1C1	0.27614	0.30435	0.05924	0.21511	0.88959	0.52976	0.15001	0.09674	0.2009
M1C3	0.26893	0.31851	0.04064	0.33356	0.38951	0.30339	0.08298	0.10013	0.06621
M1C5	0.3203	0.30445	0.06215	0.29666	0.88987	0.30426	0.06619	0.17325	0.06157

LPS	7.95576	234.59	105.566	6.35548	65.7993	73.4148	0.23717	1.31221	0.25069
LPS	8.76649	456.351	45.3176	3.85839	206.5	136.051	0.26869	1.15829	0.26683
LPS	8.70594	139.488	80.5605	4.65248	140.07	51.5536	0.37475	1.11111	0.32851
LPS	6.97407	280.917	54.6443	4.62034	43.4113	112.05	0.35948	1.15029	0.31732
M1C1	4.11816	137.568	32.2673	2.59908	1.89212	1.41225	0.31295	1.20748	0.3002
M1C1	4.96571	24.6583	25.1415	1.82513	1.91853	11.0655	0.19398	1.49692	0.13067
M1C1	4.1468	42.9326	26.7599	3.31268	18.2522	12.7994	0.17123	1.31221	0.09176
M1C1	1.0367	32.991	25.8483	4.88379	126.238	12.978	0.16087	1.62676	0.10988
M1C1	2.51751	82.9402	33.8715	6.35548	102.537	1.2466	0.12192	0.9151	0.11534
M1C3	2.28469	28.3249	33.6375	3.13399		7.93373	0.17727	1.58227	0.11534
M1C3	1.81756	84.098	36.5551	7.15028	537.455	28.8001	0.18608	1.58227	0.11695
M1C3	1.61552	57.4406	28.2857	4.22221	385.343	20.9372	0.16771	1.05117	0.10041
M1C3	4.89735	15.6057	31.6032	1.31768	99.7331	6.48902	0.13622	1.02243	0.08863
M1C3	6.46209	82.3673	32.4917	1.18756	96.3358	26.1366	0.11859	1.04391	0.08327
M1C5	6.02934	14.0646	45.3176	8.04448	14.0257	5.45659	0.13908	1.0367	0.0905
M1C5	5.90526	50.3528	37.0654	3.67565	44.6318	21.9782	0.18225	0.44504	0.11455
M1C5	2.43176	98.6331	43.7739	17.2437	212.306	31.7349	0.16087	1.15829	0.10041
M1C5	8.52677	42.9326	55.4071	4.65248		9.83551	0.19806	1.14235	0.12535
M1C5	1.44593	37.8967	45.6328	3.2898	1.6358	10.1824	0.14299	0.84792	0.09176

Inflammation Related Genes_24 hr

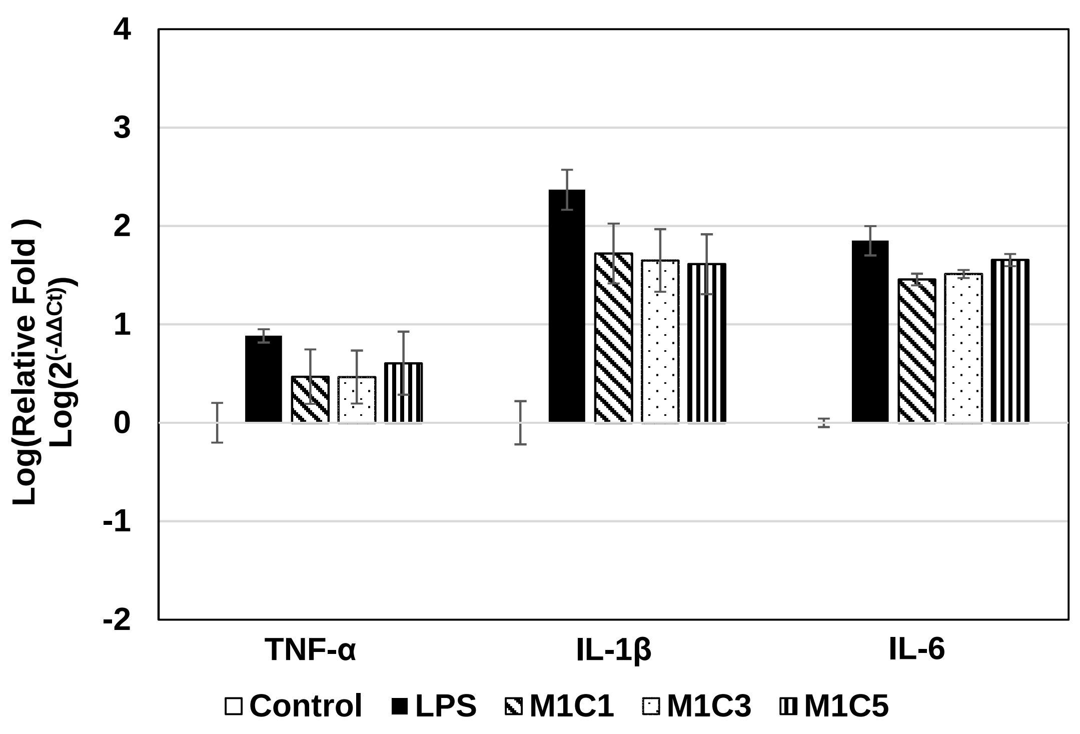

Anti-Inflammation Related Genes_ 24 hr

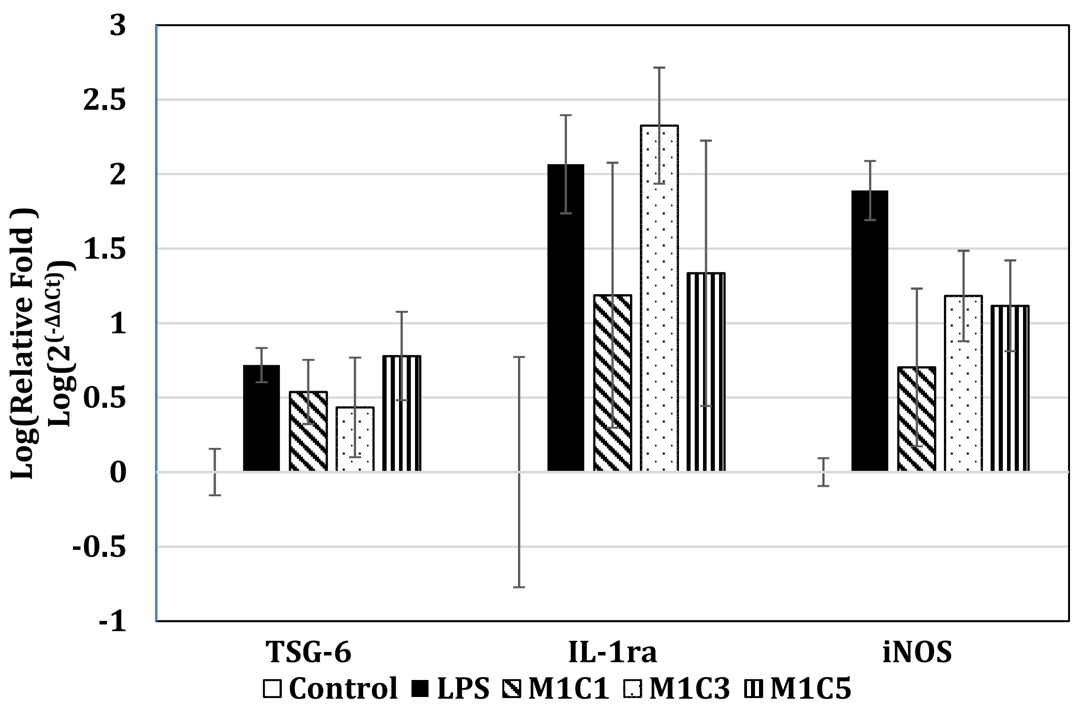

ECM Related Genes_24 hr

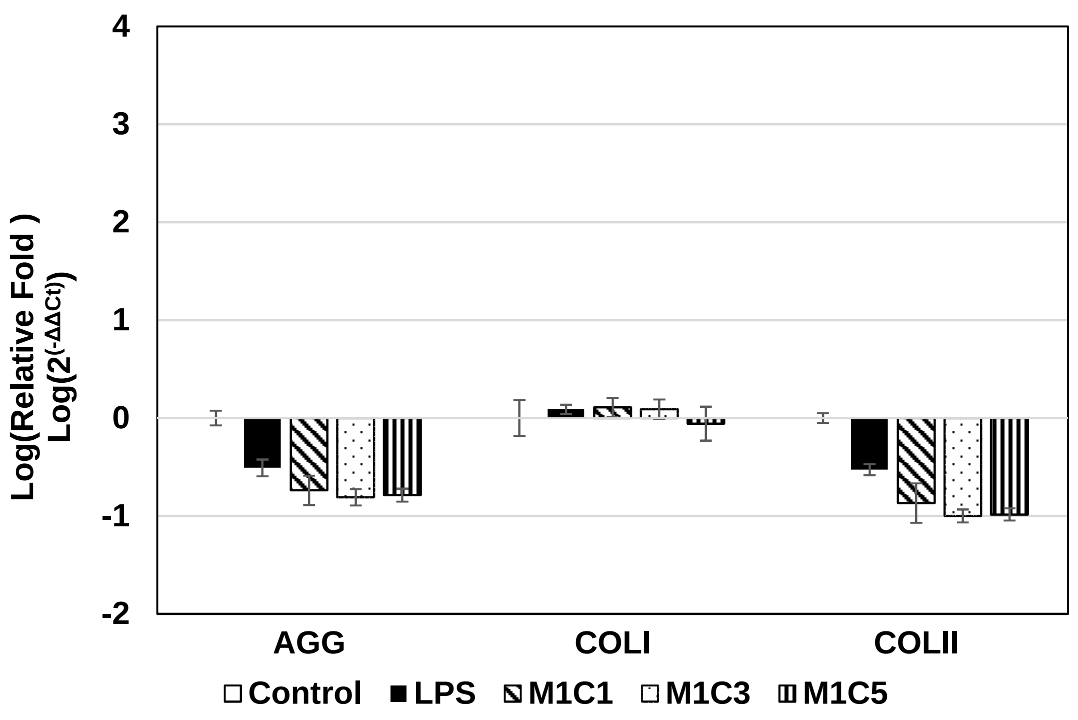

ECM Related Genes_Day 1

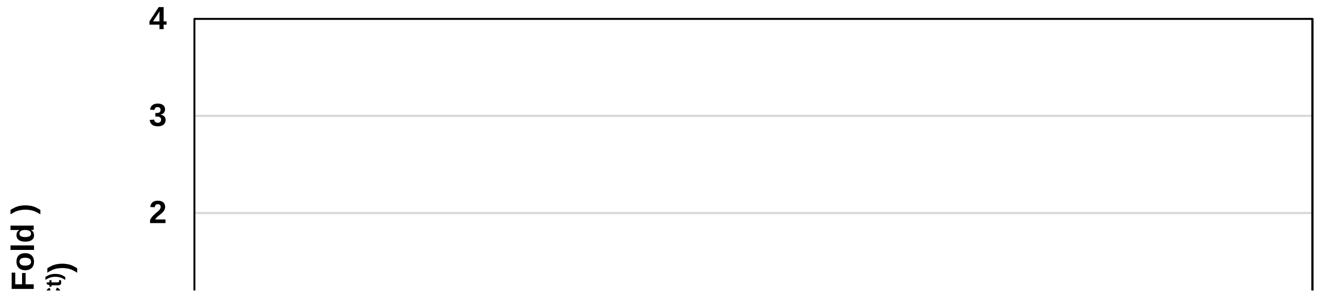

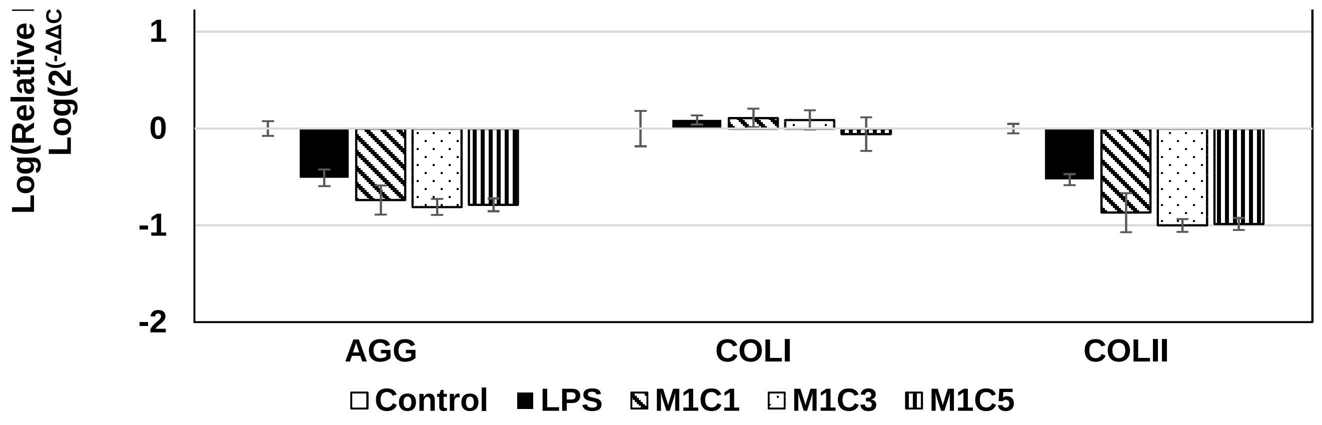
Log(Relative
Log(2^(-ΔΔC
1
0
-1
-2
AGG
COLI
COLII
Control
LPS
M1C1
M1C3
M1C5
