# Supplementary material for: Can mesenchymal stem cells and their conditioned medium assist inflammatory chondrocytes recovery?
Source: PLoS One. 2018 Nov 21;13(11):e0205563. doi: 10.1371/journal.pone.0205563 (PMC6248915; doi:10.1371/journal.pone.0205563)

Figure 4. Gene expression in MSC-chondrocyte indirect system in chondrocytes with LPS-induced inflammation.
Evaluation Time Point: 72 hr.
Indirect System

Ct value

	TNF-α	IL-1β	IL-6	iNOS	AGG	COLII	GAPDH
Control	28.35	37.25	25.58	30.57	19.23	24.59	32.53
Control	27.95	31.6	25.62	30.17	18.82	24.08	32.57
Control	29.03	35.26	25.56	30.37	18.94	24.14	32.53
Control	29.65	32.72	25.35	29.88	18.85	23.96	32.23
Control	28.11	36.76	25.51	30.9	19.05	24.34	32.65
LPS	28.12	33.41	20.32	28.75	21.03	26.37	32.57
LPS	24.15	31.56	20.82	29.06	20.86	26.46	32.54
LPS	24.39	30.65	19.53	28.55	20.77	26.13	32.25
LPS	25.5	31.09	20.27	28.35	20.93	26.24	32.27
LPS	28	31.86	20.56	28.91	20.87	26.64	32.97
M1C1	27.73	30.92	21.71	29.79	20.71	26.8	33.14
M1C1	28.22	30.35	21.05	29.53	20.88	26.87	32.84
M1C1	28.09	30.92	20.8	29.69	20.82	26.83	33.05
M1C1	27.78	31.71	22.23	29.21	20.8	26.72	32.86
M1C1	27.6	31.64	20.7	29.7	20.7	26.92	33.22
M1C3	28.14	31.6	21.03	29.94	20.88	26.54	33.35
M1C3	28.31	32.05	21	30.14	20.82	26.3	33.59
M1C3	30.02		21.07	29.86	21.3	26.71	33.56
M1C3	28.12	31.75	21.03	29.98	20.88	26.46	33.54
M1C3	28.2	33.09	20.46	29.61	20.73	26.05	33.15
M1C5	26.31	30.17	20.53	29.08	20.99	26.27	33.03
M1C5	28.03	33.9	24.44	33.44	21.35	27.11	36.4
M1C5	28.11	33.3	23.95	29.79	21.46	27.23	36.17
M1C5	28.6	35.26	21.02	31.67	21.17	27.34	36.93
M1C5	28.29	32.38	21.57	30.76	22.12	28.17	36.57

	TSG-6	IL-1ra	Col I	GAPDH
Control	33.7	39.18	12.34	32.29
Control	33.18	43.5	11.81	32.76
Control	34.11	47.84	11.49	32.79
Control			11.74	33.26
Control	33.62	45.54	11.59	32.71
LPS				32.83
LPS	31.3	36.55	11.44	32.8
LPS	31.93	34.81	11.53	32.71
LPS	31.65	35.36	11.58	32.7
LPS	31.8	37.19	11.67	32.84
M1C1	32.7	41.78	11.67	32.91
M1C1	33.39		11.54	33.09
M1C1	32.29	38.45	11.49	32.85
M1C1	31.75	35.68	11.2	32.87
M1C1	31.39	36	12.05	32.89
M1C3	32.43	47.03	11.28	32.91
M1C3	31.56	33.95	11.6	33.23
M1C3		33.94		32.74
M1C3	33.75		11.98	32.98
M1C3	33.69	35.97	11.74	32.77
M1C5	31.38	39.2	12.2	33.22
M1C5	30.77	35.79	11.68	31.48
M1C5	30.32	35.32	12.08	33.26
M1C5		44.94	12.07	33.23
M1C5	33.9		13.72	34.45

Step.1

ΔCt number (=Target gene Ct- GAPDH Ct)

	TNF-α	IL-1β	IL-6	TSG-6	IL-1ra	iNOS	AGG	COLI	COLII
Control	-4.18	4.72	-6.95	1.27	5.09	-1.96	-13.3	-21.91	-7.94
Control	-4.62	-0.97	-6.95	1	4.5	-2.4	-13.75	-21.72	-8.49
Control	-3.5	2.73	-6.97	2.44	8.81	-2.16	-13.59	-21.33	-8.39
Control	-2.58	0.49	-6.88			-2.35	-13.38	-21.76	-8.27
Control	-4.54	4.11	-7.14	1.58	2.72	-1.75	-13.6	-22.17	-8.31
LPS	-4.45	0.84	-12.25			-3.82	-11.54		-6.2
LPS	-8.39	-0.98	-11.72	0.7	4.03	-3.48	-11.68	-20.86	-6.08
LPS	-7.86	-1.6	-12.72	1.62	9.9	-3.7	-11.48	-21.57	-6.12
LPS	-6.77	-1.18	-12	-0.25	3.11	-3.92	-11.34	-21.64	-6.03
LPS	-4.97	-1.11	-12.41	-0.6	5.51	-4.06	-12.1	-22.16	-6.33
M1C1	-5.41	-2.22	-11.43	-0.25	6.47	-3.35	-12.43	-22.14	-6.34
M1C1	-4.62	-2.49	-11.79	-0.11		-3.31	-11.96	-22.06	-5.97
M1C1	-4.96	-2.13	-12.25	-0.02	2.5	-3.36	-12.23	-21.55	-6.22
M1C1	-5.08	-1.15	-10.63	-0.47	2.32	-3.65	-12.06	-21.16	-6.14

Control ΔCt

	TNF-α	IL-1β	IL-6	TSG-6	IL-1ra	iNOS	AGG	COLI	COLII
	-4.18	4.72	-6.95	1.27	5.09	-1.96	-13.3	-21.91	-7.94
	-4.62	-0.97	-6.95	1	4.5	-2.4	-13.75	-21.72	-8.49
	-3.5	2.73	-6.97	2.44	8.81	-2.16	-13.59	-21.33	-8.39
	-2.58	0.49	-6.88			-2.35	-13.38	-21.76	-8.27
	-4.54	4.11	-7.14	1.58	2.72	-1.75	-13.6	-22.17	-8.31
Ave.	-3.884	2.216	-6.978	1.5725	5.28	-2.124	-13.524	-21.778	-8.28
std.	0.76256	2.15644	0.08658	0.54127	2.21692	0.24303	0.16255	0.27404	0.1859

Step. 4

Log(Relative Fold (= 2^(-ΔΔCt)))

	TNF-α	IL-1β	IL-6	TSG-6	IL-1ra	iNOS	AGG	COLI	COLII
Control	0.0891	-0.7538	-0.0084	0.09106	0.0572	-0.0494	-0.0674	0.03974	-0.1024
Control	0.22156	0.95908	-0.0084	0.17234	0.2348	0.08308	0.06803	-0.0175	0.06322
Control	-0.1156	-0.1547	-0.0024	-0.2611	-1.0626	0.01084	0.01987	-0.1349	0.03311

M1C1	-5.62	-1.58	-12.52	-1.28	7.23	-3.52	-12.52	-21.79	-6.3
M1C3	-5.21	-1.75	-12.32	-0.62	7.9	-3.41	-12.47	-21.75	-6.81
M1C3	-5.28	-1.54	-12.59	1.37	7.07	-3.45	-12.77	-20.21	-7.29
M1C3	-3.54		-12.49		9.39	-3.7	-12.26		-6.85
M1C3	-5.42	-1.79	-12.51	0.26		-3.56	-12.66	-22.27	-7.08
M1C3	-4.95	-0.06	-12.69	1.3	7.16	-3.54	-12.42	-21.67	-7.1
M1C5	-6.72	-2.86	-12.5	-1.32	9.07	-3.95	-12.04	-22.42	-6.76
M1C5	-8.37	-2.5	-11.96	-1.81	2.95	-2.96	-15.05	-22.06	-9.29
M1C5	-8.06	-2.87	-12.22	0.04	3.28	-6.38	-14.71	-20.75	-8.94
M1C5	-8.33	-1.67	-15.91		11.14	-5.26	-15.76	-19.63	-9.59
M1C5	-8.28	-4.19	-15	-1.09		-5.81	-14.45	-21.42	-8.4

Step. 2

ΔΔCt (=Experimal ΔCt- Control ΔCt)

	TNF-α	IL-1β	IL-6	TSG-6	IL-1ra	iNOS	AGG	COLI	COLII
Control	-0.296	2.504	0.028	-0.3025	-0.19	0.164	0.224	-0.132	0.34
Control	-0.736	-3.186	0.028	-0.5725	-0.78	-0.276	-0.226	0.058	-0.21
Control	0.384	0.514	0.008	0.8675	3.53	-0.036	-0.066	0.448	-0.11
Control	1.304	-1.726	0.098			-0.226	0.144	0.018	0.01
Control	-0.656	1.894	-0.162	0.0075	-2.56	0.374	-0.076	-0.392	-0.03
LPS	-0.566	-1.376	-5.272			-1.696	1.984		2.08
LPS	-4.506	-3.196	-4.742	-0.8725	-1.25	-1.356	1.844	0.918	2.2
LPS	-3.976	-3.816	-5.742	0.0475	4.62	-1.576	2.044	0.208	2.16
LPS	-2.886	-3.396	-5.022	-1.8225	-2.17	-1.796	2.184	0.138	2.25
LPS	-1.086	-3.326	-5.432	-2.1725	0.23	-1.936	1.424	-0.382	1.95
M1C1	-1.526	-4.436	-4.452	-1.8225	1.19	-1.226	1.094	-0.362	1.94
M1C1	-0.736	-4.706	-4.812	-1.6825		-1.186	1.564	-0.282	2.31
M1C1	-1.076	-4.346	-5.272	-1.5925	-2.78	-1.236	1.294	0.228	2.06
M1C1	-1.196	-3.366	-3.652	-2.0425	-2.96	-1.526	1.464	0.618	2.14
M1C1	-1.736	-3.796	-5.542	-2.8525	1.95	-1.396	1.004	-0.012	1.98
M1C3	-1.326	-3.966	-5.342	-2.1925	2.62	-1.286	1.054	0.028	1.47
M1C3	-1.396	-3.756	-5.612	-0.2025	1.79	-1.326	0.754	1.568	0.99
M1C3	0.344		-5.512		4.11	-1.576	1.264		1.43
M1C3	-1.536	-4.006	-5.532	-1.3125		-1.436	0.864	-0.492	1.2
M1C3	-1.066	-2.276	-5.712	-0.2725	1.88	-1.416	1.104	0.108	1.18
M1C5	-2.836	-5.076	-5.522	-2.8925	3.79	-1.826	1.484	-0.642	1.52
M1C5	-4.486	-4.716	-4.982	-3.3825	-2.33	-0.836	-1.526	-0.282	-1.01
M1C5	-4.176	-5.086	-5.242	-1.5325	-2	-4.256	-1.186	1.028	-0.66
M1C5	-4.446	-3.886	-8.932		5.86	-3.136	-2.236	2.148	-1.31
M1C5	-4.396	-6.406	-8.022	-2.6625		-3.686	-0.926	0.358	-0.12

Step. 3

Relative Fold (= 2^(-ΔΔCt))

	TNF-α	IL-1β	IL-6	TSG-6	IL-1ra	iNOS	AGG	COLI	COLII
Control	1.22774	0.17629	0.98078	1.23328	1.14076	0.89255	0.85619	1.09581	0.79004
Control	1.66555	9.10084	0.98078	1.4871	1.71713	1.21083	1.16959	0.96059	1.15669
Control	0.76631	0.70028	0.99447	0.5481	0.08657	1.02527	1.04681	0.73306	1.07923
Control	0.405	3.30809	0.93433			1.16959	0.90501	0.9876	0.99309
Control	1.57571	0.26906	1.11884	0.99481	5.89708	0.77164	1.05409	1.31221	1.02101

Control	-0.3925	0.51958	-0.0295			0.06803	-0.0433	-0.0054	-0.003
Control	0.19748	-0.5702	0.04877	-0.0023	0.77064	-0.1126	0.02288	0.118	0.00903
LPS	0.17038	0.41422	1.58703			0.51055	-0.5972		-0.6261
LPS	1.35644	0.96209	1.42748	0.26265	0.37629	0.4082	-0.5551	-0.2763	-0.6623
LPS	1.1969	1.14873	1.72851	-0.0143	-1.3908	0.47442	-0.6153	-0.0626	-0.6502
LPS	0.86877	1.0223	1.51177	0.54863	0.65324	0.54065	-0.6574	-0.0415	-0.6773
LPS	0.32692	1.00123	1.63519	0.65399	-0.0692	0.58279	-0.4287	0.11499	-0.587
M1C1	0.45937	1.33537	1.34019	0.54863	-0.3582	0.36906	-0.3293	0.10897	-0.584
M1C1	0.22156	1.41665	1.44856	0.50648		0.35702	-0.4708	0.08489	-0.6954
M1C1	0.32391	1.30828	1.58703	0.47939	0.83686	0.37207	-0.3895	-0.0686	-0.6201
M1C1	0.36003	1.01327	1.09936	0.61485	0.89105	0.45937	-0.4407	-0.186	-0.6442
M1C1	0.52259	1.14271	1.66831	0.85869	-0.587	0.42024	-0.3022	0.00361	-0.596
M1C3	0.39917	1.19388	1.6081	0.66001	-0.7887	0.38712	-0.3173	-0.0084	-0.4425
M1C3	0.42024	1.13067	1.68938	0.06096	-0.5388	0.39917	-0.227	-0.472	-0.298
M1C3	-0.1036		1.65928		-1.2372	0.47442	-0.3805		-0.4305
M1C3	0.46238	1.20593	1.6653	0.3951		0.43228	-0.2601	0.14811	-0.3612
M1C3	0.3209	0.68514	1.71948	0.08203	-0.5659	0.42626	-0.3323	-0.0325	-0.3552
M1C5	0.85372	1.52803	1.66229	0.87073	-1.1409	0.54968	-0.4467	0.19326	-0.4576
M1C5	1.35042	1.41966	1.49973	1.01823	0.7014	0.25166	0.45937	0.08489	0.30404
M1C5	1.2571	1.53104	1.578	0.46133	0.60206	1.28118	0.35702	-0.3095	0.19868
M1C5	1.33838	1.1698	2.6888		-1.764	0.94403	0.6731	-0.6466	0.39435
M1C5	1.32333	1.9284	2.41486	0.80149		1.1096	0.27875	-0.1078	0.03612

Step. 5

Ave.

	TNF-α	IL-1β	IL-6	TSG-6	IL-1ra	iNOS	AGG	COLI	COLII
Control	8.9E-17	0	-1E-16	0	0	-4E-17	4.5E-16	4.3E-16	-4E-16
LPS	0.78388	0.90971	1.578	0.36274	-0.1076	0.50332	-0.5708	-0.0664	-0.6406
M1C1	0.37749	1.24325	1.42869	0.60161	0.19567	0.39555	-0.3865	-0.0114	-0.6279
M1C3	0.29983	1.05391	1.66831	0.29952	-0.7827	0.42385	-0.3034	-0.0912	-0.3775
M1C5	1.22459	1.51538	1.96874	0.78795	-0.4004	0.82723	0.2643	-0.1571	0.09513

std.

	TNF-α	IL-1β	IL-6	TSG-6	IL-1ra	iNOS	AGG	COLI	COLII
Control	0.25665	0.72577	0.02914	0.18814	0.7706	0.08179	0.05471	0.09223	0.06257
LPS	0.52221	0.28567	0.11516	0.30086	0.90572	0.06642	0.08752	0.16084	0.03532
M1C1	0.11753	0.16262	0.22314	0.15253	0.77762	0.04307	0.07146	0.12017	0.04422
M1C3	0.23127	0.24805	0.04117	0.28478	0.32305	0.03388	0.0606	0.2662	0.0594
M1C5	0.21043	0.27367	0.54408	0.23576	1.24187	0.42064	0.42413	0.33413	0.33647

LPS	1.48041	2.59548	38.6394			3.24001	0.25279		0.23651
LPS	22.7217	9.16414	26.7599	1.83083	2.37841	2.55974	0.27855	0.52924	0.21764
LPS	15.736	14.0841	53.5198	0.96761	0.04067	2.98142	0.24249	0.86574	0.22376
LPS	7.39218	10.5268	32.4917	3.53694	4.50023	3.47256	0.22006	0.90878	0.21022
LPS	2.12285	10.0283	43.1713	4.50804	0.85263	3.82643	0.37268	1.30315	0.25882
M1C1	2.87986	21.6456	21.887	3.53694	0.4383	2.33918	0.46846	1.28521	0.26062
M1C1	1.66555	26.1004	28.0903	3.20984		2.27521	0.33821	1.21588	0.20166
M1C1	2.10818	20.3365	38.6394	3.01571	6.86852	2.35545	0.40782	0.85382	0.23982
M1C1	2.29104	10.3102	12.5708	4.11959	7.78124	2.87986	0.36249	0.65157	0.22688
M1C1	3.3311	13.8902	46.5917	7.22251	0.25882	2.63171	0.49862	1.00835	0.25349
M1C3	2.50707	15.6273	40.5604	4.57097	0.16267	2.43851	0.48163	0.98078	0.36098
M1C3	2.63171	13.5104	48.908	1.15069	0.28917	2.50707	0.59296	0.33728	0.50348
M1C3	0.78785		45.6328		0.05791	2.98142	0.41639		0.37113
M1C3	2.89989	16.0667	46.2698	2.48372		2.7057	0.54943	1.40639	0.43528
M1C3	2.09362	4.84333	52.4183	1.2079	0.27168	2.66845	0.46522	0.92787	0.44135
M1C5	7.14038	33.7309	45.9502	7.42556	0.07229	3.54553	0.3575	1.56049	0.34869
M1C5	22.4089	26.2819	31.6032	10.4288	5.02805	1.78509	2.87986	1.21588	2.01391
M1C5	18.076	33.9655	37.8442	2.89287	4	19.1066	2.27521	0.49039	1.58008
M1C5	21.7961	14.7844	488.427		0.01722	8.79083	4.71089	0.22563	2.47942
M1C5	21.0537	84.8004	259.934	6.33129		12.8705	1.9	0.78025	1.08673

Anti-Inflammation Related Genes_72 hr

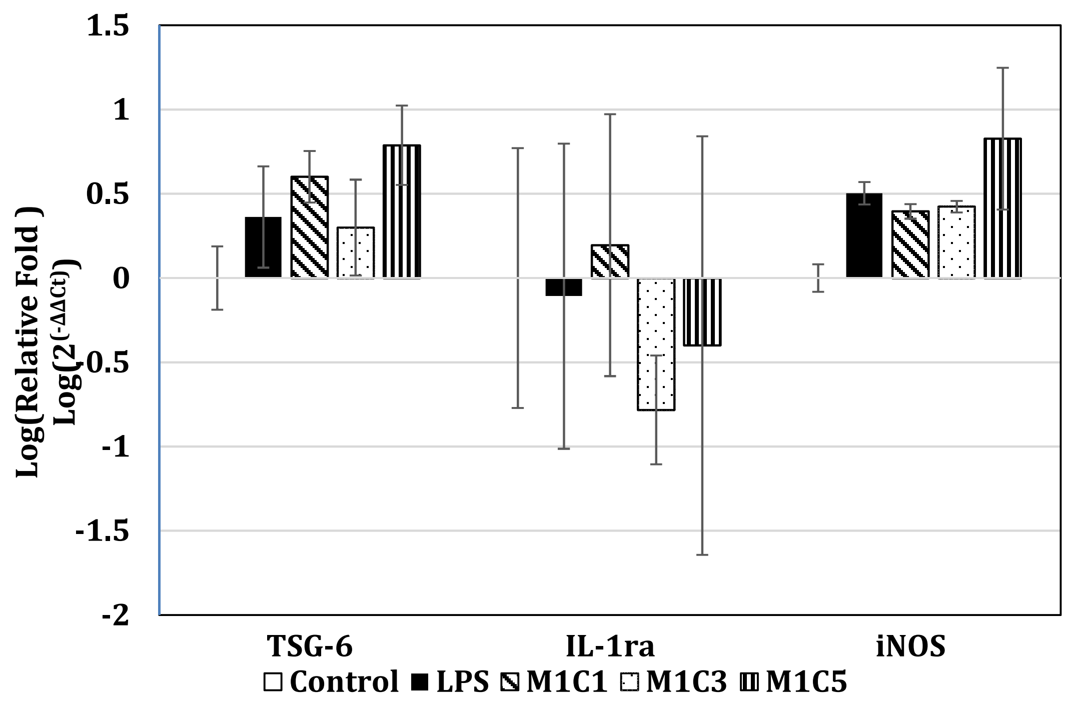

Inflammation Related Genes_72 hr

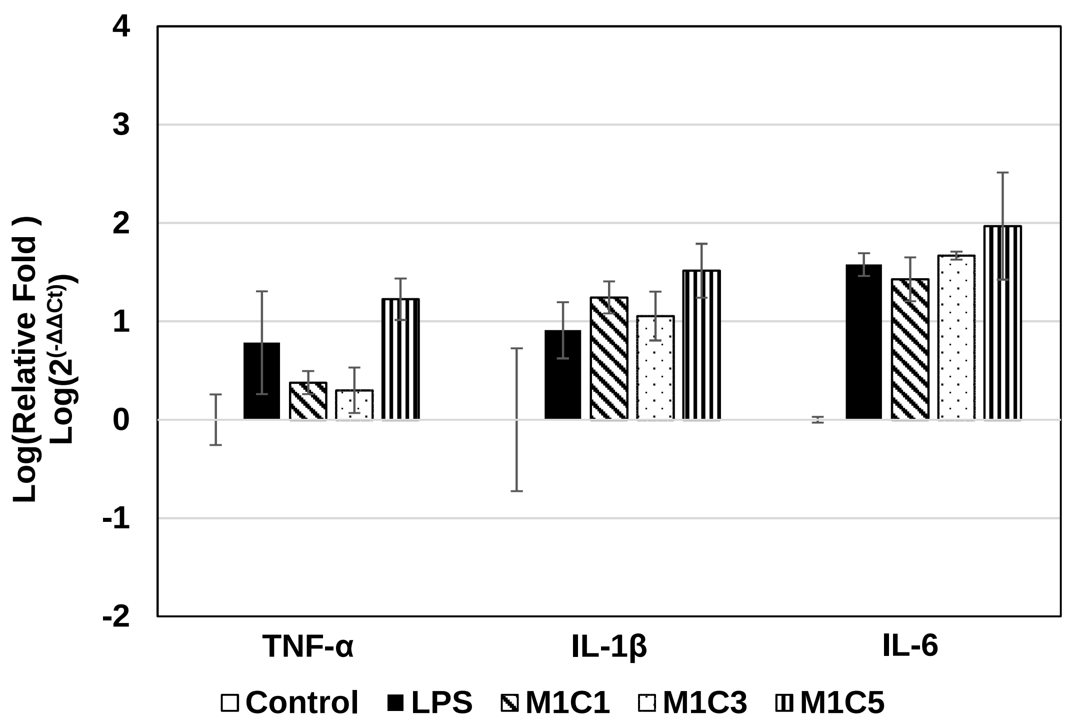

ECM Related Genes_72 hr

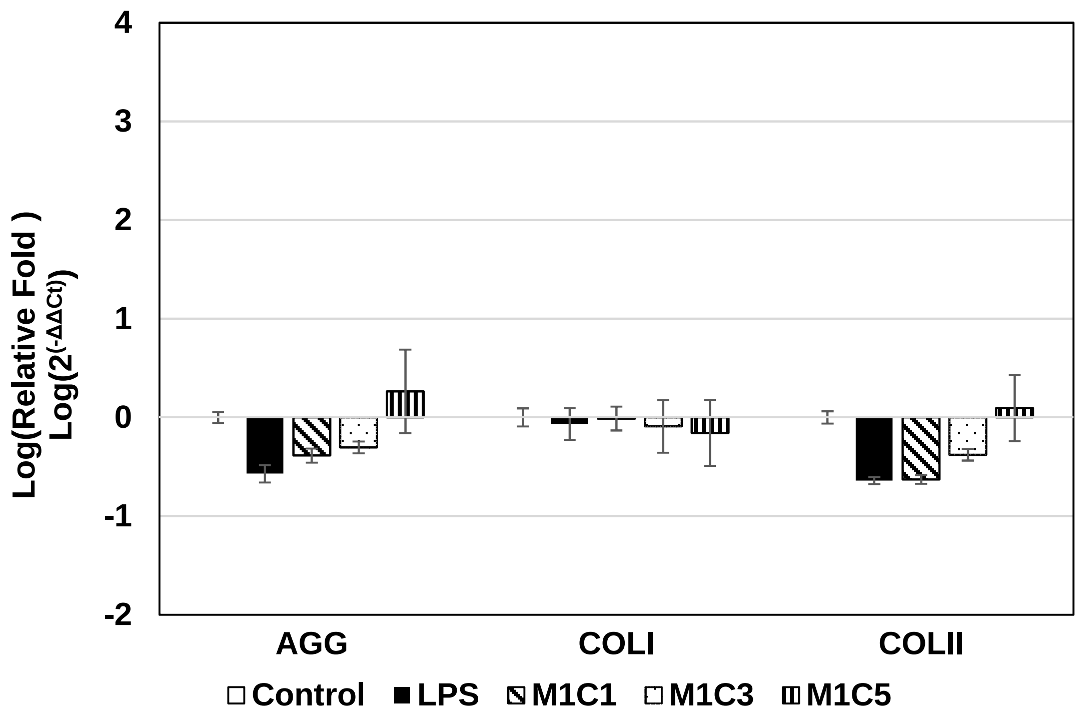

Supplement: S4 Data — (PDF) [file pone.0205563.s004.pdf]
