# Supplementary material for: Can mesenchymal stem cells and their conditioned medium assist inflammatory chondrocytes recovery?
Source: PLoS One. 2018 Nov 21;13(11):e0205563. doi: 10.1371/journal.pone.0205563 (PMC6248915; doi:10.1371/journal.pone.0205563)

Figure 5. Gene expression in MSC-chondrocyte indirect and direct system in chondrocytes with LPS-induced inflammation.
Evaluation Time Point: 24 hr.
Direct System (compared with Indirect System)

Ct value

	TNF-α	IL-1β	IL-6	iNOS	AGG	COLII	GAPDH
Control	27.83	32.72	26.83	30.95	19.56	21.77	33.48
Control	26.88	32.97	26.73	30.8	19.69	21.72	33.25
Control	28.75	33.71	26.95	31.41	19.8	21.89	33.4
Control	27.46	33.13	27.09	30.81	19.78	21.85	33.64
Control	27.49	34.19	26.31	30.52	19.84	21.74	33.13
LPS	25.33	26.25	20.66	25.55	21.58	23.64	33.64
LPS	24.57	25.35	19.94	24.58	21.69	23.67	33.26
LPS	24.52	24.48	21.25	23.78	21.6	23.67	33.35
LPS	24.69	26.35	20.58	25.34	21.28	23.53	33.51
LPS	25.12	25.45	21.25	24.33	21.45	23.69	33.62
M1C3-INI	25.23	27.26	20.45	26.65	20.97	23.65	32.12
M1C3-INI	25.53	25.66	20.33	24.76	20.87	23.6	32.09
M1C3-INI	25.52	26.03	20.49	25.04	20.84	23.64	31.91
M1C3-INI	23.85	27.84	20.26	26.66	21.07	23.75	31.84
M1C3-INI	23.55	25.54	20.32	24.75	21.37	23.94	31.94
M1C3-D	25.16	30.15	20.98	28.64	25.09	30.23	32.81
M1C3-D	26.28	29.89		29.23	24.8	29.95	33.5
M1C3-D	28.2	30.98	21.12	28.73	25.18	30.34	31.93
M1C3-D	27.85	29.15	20.25	28.67	24.9	30.44	32.01
M1C3-D			20.88	28.87	24.38	30.2	33.36
M1C3-D	27.72	31.27	20.99				31.11

	TSG-6	IL-1ra	Col I	GAPDH
Control	33.7	39.18	12.34	32.29
Control	33.18	43.5	11.81	32.76
Control	34.11		11.49	32.79
Control	35.04	41.96	11.74	33.26
Control	33.62	45.54	11.59	32.71
LPS	31.11	34.6	11.34	32.83
LPS	31.3	36.55	11.44	32.8
LPS	31.93	34.81	11.53	32.71
LPS	31.65	35.36	11.58	32.7
LPS	31.8	37.19	11.67	32.84
M1C3-INI	32.43		11.28	32.91
M1C3-INI	31.56	33.95	11.6	33.23
M1C3-INI	31.83	33.94	11.7	32.74
M1C3-INI	33.75	36.13	11.98	32.98
M1C3-INI	33.69	35.97	11.74	32.77
M1C3-D	32.83	34.52	11.91	32.81
M1C3-D		35	11.72	33.5
M1C3-D	33.73		12.22	31.93
M1C3-D	32.36	35.08	11.97	32.01
M1C3-D	32.55	33.21	11.87	33.36
M1C3-D	32.62	36.03		31.11

Step.1
ΔCt number (=Target gene Ct- GAPDH Ct)

	TNF-α	IL-1β	IL-6	TSG-6	IL-1ra	iNOS	AGG	COLI	COLII
Control	-5.65	-0.76	-6.65	1.41	6.89	-2.53	-13.92	-19.95	-11.71
Control	-6.37	-0.28	-6.52	0.42	10.74	-2.45	-13.56	-20.95	-11.53
Control	-4.65	0.31	-6.45	1.32		-1.99	-13.6	-21.3	-11.51
Control	-6.18	-0.51	-6.55	1.78	8.7	-2.83	-13.86	-21.52	-11.79
Control	-5.64	1.06	-6.82	0.91	12.83	-2.61	-13.29	-21.12	-11.39
LPS	-8.31	-7.39	-12.98	-1.72	1.77	-8.09	-12.06	-21.49	-10
LPS	-8.69	-7.91	-13.32	-1.5	3.75	-8.68	-11.57	-21.36	-9.59
LPS	-8.83	-8.87	-12.1	-0.78	2.1	-9.57	-11.75	-21.18	-9.68
LPS	-8.82	-7.16	-12.93	-1.05	2.66	-8.17	-12.23	-21.12	-9.98
LPS	-8.5	-8.17	-12.37	-1.04	4.35	-9.29	-12.17	-21.17	-9.93
M1C3-INI	-6.89	-4.86	-11.67	-0.48		-5.47	-11.15	-21.63	-8.47
M1C3-INI	-6.56	-6.43	-11.79	-1.67	0.72	-7.33	-11.22	-21.63	-8.49
M1C3-INI	-6.39	-5.88	-11.42	-0.91	1.2	-6.87	-11.07	-21.04	-8.27
M1C3-INI	-7.99	-4	-11.58	0.77	3.15	-5.18	-10.77	-21	-8.09
M1C3-INI	-8.39	-6.4	-11.62	0.92	3.2	-7.19	-10.57	-21.03	-8
M1C3-D	-7.65	-2.66	-11.83	0.02	1.71	-4.17	-7.72	-20.9	-2.58
M1C3-D	-7.22	-3.61			1.5	-4.27	-8.7	-21.78	-3.55
M1C3-D	-3.73	-0.95	-10.81	1.8		-3.2	-6.75	-19.71	-1.59
M1C3-D	-4.16	-2.86	-11.76	0.35	3.07	-3.34	-7.11	-20.04	-1.57

Control ΔCt

	TNF-α	IL-1β	IL-6	TSG-6	IL-1ra	iNOS	AGG	COLI	COLII
	-5.65	-0.76	-6.65	1.41	6.89	-2.53	-13.92	-19.95	-11.71
	-6.37	-0.28	-6.52	0.42	10.74	-2.45	-13.56	-20.95	-11.53
	-4.65	0.31	-6.45	1.32		-1.99	-13.6	-21.3	-11.51
	-6.18	-0.51	-6.55	1.78	8.7	-2.83	-13.86	-21.52	-11.79
	-5.64	1.06	-6.82	0.91	12.83	-2.61	-13.29	-21.12	-11.39
Ave.	-5.698	-0.036	-6.598	1.168	9.79	-2.482	-13.646	-20.968	-11.586
std.	0.59798	0.65258	0.12828	0.46525	2.22161	0.27672	0.22677	0.54308	0.14444

Step. 4
Log(Relative Fold (= 2^(-ΔΔCt)))

	TNF-α	IL-1β	IL-6	TSG-6	IL-1ra	iNOS	AGG	COLI	COLII
Control	-0.0144	0.21795	0.01565	-0.0728	0.87299	0.01445	0.08248	-0.3064	0.03733
Control	0.20229	0.07345	-0.0235	0.22517	-1.159	-0.0241	-0.1084	0.30103	-0.0542
Control	-0.3155	-0.1042	-0.0446	-0.0458		-0.1481	-0.0138	0.09994	-0.0229
Control	0.1451	0.14269	-0.0144	-0.1842	0.32812	0.10476	0.06442	0.16617	0.06141
Control	-0.0175	-0.3299	0.06683	0.07767	-0.9151	0.03853	-0.1072	0.04576	-0.059
LPS	0.78629	2.21377	1.92117	0.86937	2.41426	1.68818	-0.4774	0.15714	-0.4774
LPS	0.90068	2.37031	2.02352	0.80315	1.81822	1.86578	-0.6249	0.118	-0.6009

M1C3-D			-12.48	-0.81	-0.15	-4.49	-8.98	-21.49	-3.16
M1C3-D	-3.39	0.16	-10.12	1.51	4.92				

Step. 2

ΔΔCt (=Experimal ΔCt- Control ΔCt)

	TNF-α	IL-1β	IL-6	TSG-6	IL-1ra	iNOS	AGG	COLI	COLII
Control	0.048	-0.724	-0.052	0.242	-2.9	-0.048	-0.274	1.018	-0.124
Control	-0.672	-0.244	0.078	-0.748	3.85	0.08	0.36	-1	0.18
Control	1.048	0.346	0.148	0.152		0.492	0.046	-0.332	0.076
Control	-0.482	-0.474	0.048	0.612	-1.09	-0.348	-0.214	-0.552	-0.204
Control	0.058	1.096	-0.222	-0.258	3.04	-0.128	0.356	-0.152	0.196
LPS	-2.612	-7.354	-6.382	-2.888	-8.02	-5.608	1.586	-0.522	1.586
LPS	-2.992	-7.874	-6.722	-2.668	-6.04	-6.198	2.076	-0.392	1.996
LPS	-3.132	-8.834	-5.502	-1.948	-7.69	-7.088	1.896	-0.212	1.906
LPS	-3.122	-7.124	-6.332	-2.218	-7.13	-5.688	1.416	-0.152	1.606
LPS	-2.802	-8.134	-5.772	-2.208	-5.44	-6.808	1.476	-0.202	1.656
M1C3-INI	-1.192	-4.824	-5.072	-1.648		-2.988	2.496	-0.662	3.116
M1C3-INI	-0.862	-6.394	-5.192	-2.838	-9.07	-4.848	2.426	-0.662	3.096
M1C3-INI	-0.692	-5.844	-4.822	-2.078	-8.59	-4.388	2.576	-0.072	3.316
M1C3-INI	-2.292	-3.964	-4.982	-0.398	-6.64	-2.698	2.876	-0.032	3.496
M1C3-INI	-2.692	-6.364	-5.022	-0.248	-6.59	-4.708	3.076	-0.062	3.586
M1C3-D	-1.952	-2.624	-5.232	-1.148	-8.08	-1.688	5.926	0.068	9.006
M1C3-D	-1.522	-3.574			-8.29	-1.788	4.946	-0.812	8.036
M1C3-D	1.968	-0.914	-4.212	0.632		-0.718	6.896	1.258	9.996
M1C3-D	1.538	-2.824	-5.162	-0.818	-6.72	-0.858	6.536	0.928	10.016
M1C3-D			-5.882	-1.978	-9.94	-2.008	4.666	-0.522	8.426
M1C3-D	2.308	0.196	-3.522	0.342	-4.87				

Step. 3

Relative Fold (= 2^(-ΔΔCt))

	TNF-α	IL-1β	IL-6	TSG-6	IL-1ra	iNOS	AGG	COLI	COLII
Control	0.96728	1.65176	1.0367	0.84557	7.46426	1.03383	1.20916	0.4938	1.08975
Control	1.59328	1.18427	0.94737	1.67946	0.06935	0.94606	0.77916	2	0.8827
Control	0.48364	0.78676	0.9025	0.9		0.71104	0.96862	1.25876	0.94868
Control	1.39668	1.38896	0.96728	0.65429	2.12874	1.27279	1.1599	1.46612	1.15189
Control	0.96059	0.46781	1.16635	1.19582	0.12158	1.09278	0.78133	1.11111	0.87297
LPS	6.11351	163.597	83.4014	7.40244	259.574	48.7726	0.33309	1.43594	0.33309
LPS	7.95576	234.59	105.566	6.35548	65.7993	73.4148	0.23717	1.31221	0.25069
LPS	8.76649	456.351	45.3176	3.85839	206.5	136.051	0.26869	1.15829	0.26683
LPS	8.70594	139.488	80.5605	4.65248	140.07	51.5536	0.37475	1.11111	0.32851
LPS	6.97407	280.917	54.6443	4.62034	43.4113	112.05	0.35948	1.15029	0.31732
M1C3-INI	2.28469	28.3249	33.6375	3.13399		7.93373	0.17727	1.58227	0.11534
M1C3-INI	1.81756	84.098	36.5551	7.15028	537.455	28.8001	0.18608	1.58227	0.11695
M1C3-INI	1.61552	57.4406	28.2857	4.22221	385.343	20.9372	0.16771	1.05117	0.10041
M1C3-INI	4.89735	15.6057	31.6032	1.31768	99.7331	6.48902	0.13622	1.02243	0.08863
M1C3-INI	6.46209	82.3673	32.4917	1.18756	96.3358	26.1366	0.11859	1.04391	0.08327
M1C3-D	3.86911	6.16457	37.5828	2.21606	270.597	3.2221	0.01645	0.95396	0.00195
M1C3-D	2.87189	11.9092			312.996	3.45336	0.03244	1.75564	0.00381
M1C3-D	0.25561	1.88426	18.5327	0.64528		1.6449	0.0084	0.41812	0.00098
M1C3-D	0.34436	7.08123	35.8028	1.76296	105.42	1.81252	0.01078	0.52559	0.00097

LPS	0.94283	2.6593	1.65627	0.58641	2.31492	2.1337	-0.5708	0.06382	-0.5738
LPS	0.93982	2.14454	1.90612	0.66768	2.14634	1.71226	-0.4263	0.04576	-0.4835
LPS	0.84349	2.44858	1.73755	0.66467	1.6376	2.04941	-0.4443	0.06081	-0.4985
M1C3-INI	0.35883	1.45217	1.52682	0.4961		0.89948	-0.7514	0.19928	-0.938
M1C3-INI	0.25949	1.92479	1.56295	0.85432	2.73034	1.45939	-0.7303	0.19928	-0.932
M1C3-INI	0.20831	1.75922	1.45157	0.62554	2.58585	1.32092	-0.7755	0.02167	-0.9982
M1C3-INI	0.68996	1.19328	1.49973	0.11981	1.99884	0.81218	-0.8658	0.00963	-1.0524
M1C3-INI	0.81037	1.91575	1.51177	0.07466	1.98379	1.41725	-0.926	0.01866	-1.0795
M1C3-D	0.58761	0.7899	1.57499	0.34558	2.43232	0.50814	-1.7839	-0.0205	-2.7111
M1C3-D	0.45817	1.07588			2.49554	0.53824	-1.4889	0.24444	-2.4191
M1C3-D	-0.5924	0.27514	1.26794	-0.1903		0.21614	-2.0759	-0.3787	-3.0091
M1C3-D	-0.463	0.85011	1.55392	0.24624	2.02292	0.25828	-1.9675	-0.2794	-3.0151
M1C3-D			1.77066	0.59544	2.99224	0.60447	-1.4046	0.15714	-2.5365
M1C3-D	-0.6948	-0.059	1.06023	-0.103	1.46602				

Step. 5

Ave.

	TNF-α	IL-1β	IL-6	TSG-6	IL-1ra	iNOS	AGG	COLI	COLII
Control	3.5E-17	0	-1E-16	0	-0.2182	-0.0029	-0.0165	0.06129	-0.0075
LPS	0.88262	2.3673	1.84893	0.71826	2.06627	1.88987	-0.5087	0.0891	-0.5268
M1C3-INI	0.46539	1.64904	1.51057	0.43409	2.3247	1.18184	-0.8098	0.08971	-1
M1C3-D	-0.1409	0.58641	1.44555	0.17881	2.28181	0.42505	-1.7442	-0.0554	-2.7382

std.

	TNF-α	IL-1β	IL-6	TSG-6	IL-1ra	iNOS	AGG	COLI	COLII
Control	0.20126	0.21963	0.04317	0.15658	0.97637	0.09373	0.09085	0.22663	0.05438
LPS	0.06714	0.20315	0.14874	0.11495	0.32963	0.19857	0.08556	0.04686	0.05658
M1C1	0.26893	0.31851	0.04064	0.33356	0.38951	0.30339	0.08298	0.10013	0.06621
M1C3	0.61319	0.46475	0.28027	0.32463	0.57131	0.17561	0.2924	0.26971	0.2708

M1C3-D			58.9737	3.93947	982.286	4.02224	0.03939	1.43594	0.00291
M1C3-D	0.20194	0.87297	11.4876	0.78895	29.2426				

Inflammation Related Genes_24 hr

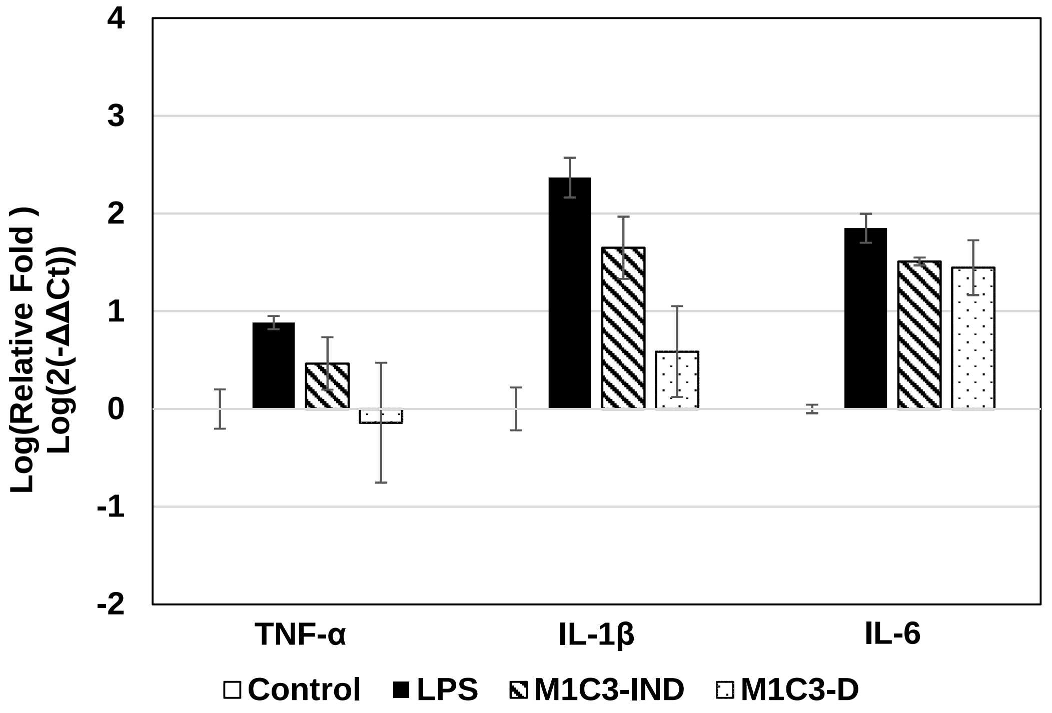

Anti-Inflammation Related Genes_24 hr

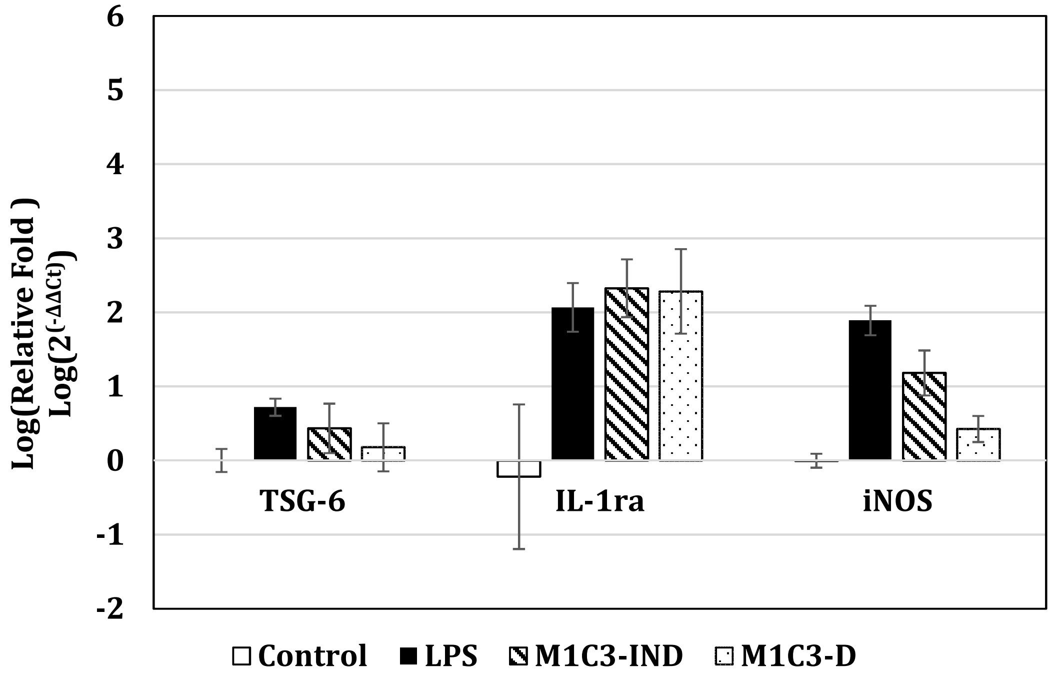

ECM Related Genes_24 hr

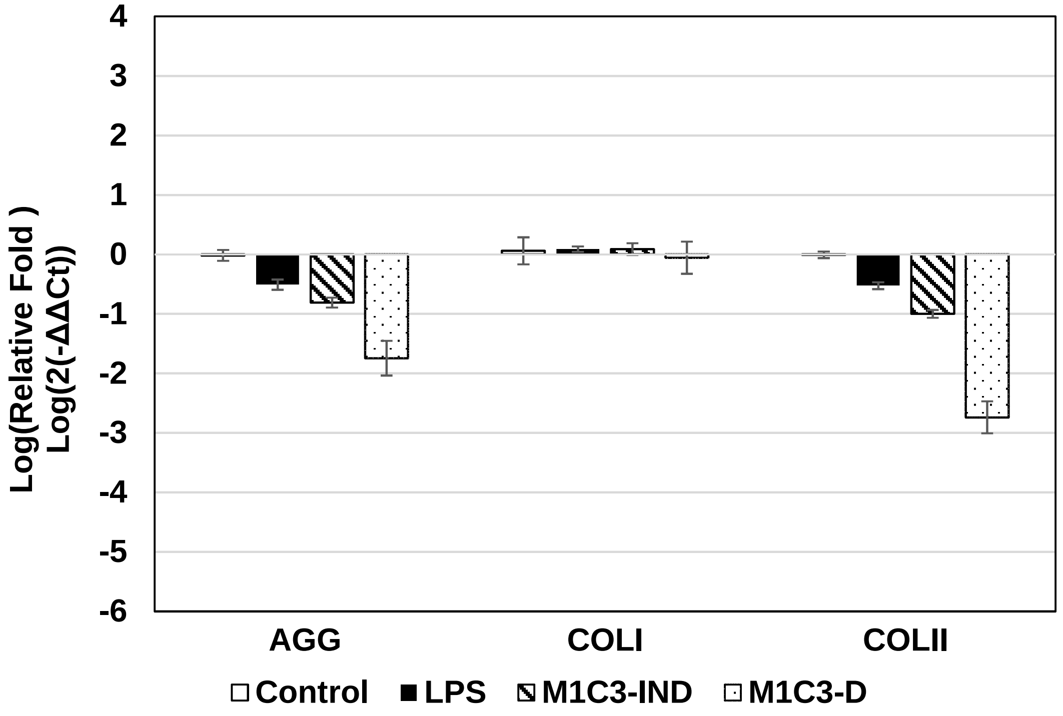

Supplement: S5 Data — (PDF) [file pone.0205563.s005.pdf]
