# Supplementary material for: Can mesenchymal stem cells and their conditioned medium assist inflammatory chondrocytes recovery?
Source: PLoS One. 2018 Nov 21;13(11):e0205563. doi: 10.1371/journal.pone.0205563 (PMC6248915; doi:10.1371/journal.pone.0205563)

Figure 5. Gene expression in MSC-chondrocyte indirect and direct system in chondrocytes with LPS-induced inflammation.
Evaluation Time Point: 72 hr.
Direct System (compared with Indirect System)

Ct value

	TNF-α	IL-1β	IL-6	iNOS	AGG	COLII	GAPDH
Control	28.35	37.25	25.58	30.57	19.23	24.59	32.53
Control	27.95	31.6	25.62	30.17	18.82	24.08	32.57
Control	29.03	35.26	25.56	30.37	18.94	24.14	32.53
Control	29.65	32.72	25.35	29.88	18.85	23.96	32.23
Control	28.11	36.76	25.51	30.9	19.05	24.34	32.65
LPS	28.12	33.41	20.32	28.75	21.03	26.37	32.57
LPS	24.15	31.56	20.82	29.06	20.86	26.46	32.54
LPS	24.39	30.65	19.53	28.55	20.77	26.13	32.25
LPS	25.5	31.09	20.27	28.35	20.93	26.24	32.27
LPS	28	31.86	20.56	28.91	20.87	26.64	32.97
M1C3-INI	28.14	31.6	21.03	29.94	20.88	26.54	33.35
M1C3-INI	28.31	32.05	21	30.14	20.82	26.3	33.59
M1C3-INI	30.02		21.07	29.86	21.3	26.71	33.56
M1C3-INI	28.12	31.75	21.03	29.98	20.88	26.46	33.54
M1C3-INI	28.2	33.09	20.46	29.61	20.73	26.05	33.15
M1C3-D	24.68	32.84	18.13	31.02	26.68	31.35	33.93
M1C3-D	26.88	33.01	19.07	31.58	27.93	32.65	34.48
M1C3-D	23.96	32.93	18.14	30.83	26.64	31.74	32.02
M1C3-D	25.65	30.33	18.05	30.76	27.64	32.23	34.17
M1C3-D	24.05	30.85	17.94	30.5	26.46	32.17	33.63
M1C3-D	25.87	31.09	18.13	29.51	27.76	32.21	31.69

	TSG-6	IL-1ra	Col I	GAPDH
Control	33.7	39.18	12.34	32.29
Control	33.18	43.5	11.81	32.76
Control	34.11	47.84	11.49	32.79
Control			11.74	33.26
Control	33.62	45.54	11.59	32.71
LPS				32.83
LPS	31.3	36.55	11.44	32.8
LPS	31.93	34.81	11.53	32.71
LPS	31.65	35.36	11.58	32.7
LPS	31.8	37.19	11.67	32.84
M1C3-INI	32.43	47.03	11.28	32.91
M1C3-INI	31.56	33.95	11.6	33.23
M1C3-IND		33.94		32.74
M1C3-INI	33.75		11.98	32.98
M1C3-INI	33.69	35.97	11.74	32.77
M1C3-D	30.57	34.59	12.64	33.93
M1C3-D	31.89	43.37	13.87	34.48
M1C3-D	31.1	36.34	13.78	32.02
M1C3-D	30.72	40.57	13.77	34.17
M1C3-D	29.78	42.36	13.6	33.63
M1C3-D	30.18	36.29	13.96	31.69

Step.1

ΔCt number (=Target gene Ct- GAPDH Ct)

	TNF-α	IL-1β	IL-6	TSG-6	IL-1ra	iNOS	AGG	COLI	COLII
Control	-4.18	4.72	-6.95	1.27	5.09	-1.96	-13.3	-21.91	-7.94
Control	-4.62	-0.97	-6.95	1	4.5	-2.4	-13.75	-21.72	-8.49
Control	-3.5	2.73	-6.97	2.44	8.81	-2.16	-13.59	-21.33	-8.39
Control	-2.58	0.49	-6.88			-2.35	-13.38	-21.76	-8.27
Control	-4.54	4.11	-7.14	1.58	2.72	-1.75	-13.6	-22.17	-8.31
LPS	-4.45	0.84	-12.25			-3.82	-11.54		-6.2
LPS	-8.39	-0.98	-11.72	0.7	4.03	-3.48	-11.68	-20.86	-6.08
LPS	-7.86	-1.6	-12.72	1.62	9.9	-3.7	-11.48	-21.57	-6.12
LPS	-6.77	-1.18	-12	-0.25	3.11	-3.92	-11.34	-21.64	-6.03
LPS	-4.97	-1.11	-12.41	-0.6	5.51	-4.06	-12.1	-22.16	-6.33
M1C3-INI	-5.21	-1.75	-12.32	-0.62	7.9	-3.41	-12.47	-21.75	-6.81
M1C3-INI	-5.28	-1.54	-12.59	1.37	7.07	-3.45	-12.77	-20.21	-7.29
M1C3-INI	-3.54		-12.49		9.39	-3.7	-12.26		-6.85
M1C3-INI	-5.42	-1.79	-12.51	0.26		-3.56	-12.66	-22.27	-7.08
M1C3-INI	-4.95	-0.06	-12.69	1.3	7.16	-3.54	-12.42	-21.67	-7.1
M1C3-D	-9.25	-1.09	-15.8	-3.36	0.66	-2.91	-7.25	-21.29	-2.58
M1C3-D	-7.6	-1.47	-15.41	-2.59	8.89	-2.9	-6.55	-20.61	-1.83
M1C3-D	-8.06	0.91	-13.88	-0.92	4.32	-1.19	-5.38	-18.24	-0.28

Control ΔCt

	TNF-α	IL-1β	IL-6	TSG-6	IL-1ra	iNOS	AGG	COLI	COLII
	-4.18	4.72	-6.95	1.27	5.09	-1.96	-13.3	-21.91	-7.94
	-4.62	-0.97	-6.95	1	4.5	-2.4	-13.75	-21.72	-8.49
	-3.5	2.73	-6.97	2.44	8.81	-2.16	-13.59	-21.33	-8.39
	-2.58	0.49	-6.88			-2.35	-13.38	-21.76	-8.27
	-4.54	4.11	-7.14	1.58	2.72	-1.75	-13.6	-22.17	-8.31
Ave.	-3.884	2.216	-6.978	1.5725	5.28	-2.124	-13.524	-21.778	-8.28
std.	0.76256	2.15644	0.08658	0.54127	2.21692	0.24303	0.16255	0.27404	0.1859

Step. 4

Log(Relative Fold (= 2^(-ΔΔCt)))

	TNF-α	IL-1β	IL-6	TSG-6	IL-1ra	iNOS	AGG	COLI	COLII
Control	0.0891	-0.7538	-0.0084	0.09106	0.0572	-0.0494	-0.0674	0.03974	-0.1024
Control	0.22156	0.95908	-0.0084	0.17234	0.2348	0.08308	0.06803	-0.0175	0.06322
Control	-0.1156	-0.1547	-0.0024	-0.2611	-1.0626	0.01084	0.01987	-0.1349	0.03311
Control	-0.3925	0.51958	-0.0295			0.06803	-0.0433	-0.0054	-0.003
Control	0.19748	-0.5702	0.04877	-0.0023	0.77064	-0.1126	0.02288	0.118	0.00903
LPS	0.17038	0.41422	1.58703			0.51055	-0.5972		-0.6261
LPS	1.35644	0.96209	1.42748	0.26265	0.37629	0.4082	-0.5551	-0.2763	-0.6623

M1C3-D	-8.52	-3.84	-16.12	-3.45	6.4	-3.41	-6.53	-20.4	-1.94
M1C3-D	-9.58	-2.78	-15.69	-3.85	8.73	-3.13	-7.17	-20.03	-1.46
M1C3-D	-5.82	-0.6	-13.56	-1.51	4.6	-2.18	-3.93	-17.73	0.52

Step. 2

ΔΔCt (=Experimal ΔCt- Control ΔCt)

	TNF-α	IL-1β	IL-6	TSG-6	IL-1ra	iNOS	AGG	COLI	COLII
Control	-0.296	2.504	0.028	-0.3025	-0.19	0.164	0.224	-0.132	0.34
Control	-0.736	-3.186	0.028	-0.5725	-0.78	-0.276	-0.226	0.058	-0.21
Control	0.384	0.514	0.008	0.8675	3.53	-0.036	-0.066	0.448	-0.11
Control	1.304	-1.726	0.098			-0.226	0.144	0.018	0.01
Control	-0.656	1.894	-0.162	0.0075	-2.56	0.374	-0.076	-0.392	-0.03
LPS	-0.566	-1.376	-5.272			-1.696	1.984		2.08
LPS	-4.506	-3.196	-4.742	-0.8725	-1.25	-1.356	1.844	0.918	2.2
LPS	-3.976	-3.816	-5.742	0.0475	4.62	-1.576	2.044	0.208	2.16
LPS	-2.886	-3.396	-5.022	-1.8225	-2.17	-1.796	2.184	0.138	2.25
LPS	-1.086	-3.326	-5.432	-2.1725	0.23	-1.936	1.424	-0.382	1.95
M1C3-INI	-1.326	-3.966	-5.342	-2.1925	2.62	-1.286	1.054	0.028	1.47
M1C3-INI	-1.396	-3.756	-5.612	-0.2025	1.79	-1.326	0.754	1.568	0.99
M1C3-INI	0.344		-5.512		4.11	-1.576	1.264		1.43
M1C3-INI	-1.536	-4.006	-5.532	-1.3125		-1.436	0.864	-0.492	1.2
M1C3-INI	-1.066	-2.276	-5.712	-0.2725	1.88	-1.416	1.104	0.108	1.18
M1C3-D	-5.366	-3.306	-8.822	-4.9325	-4.62	-0.786	6.274	0.488	5.7
M1C3-D	-3.716	-3.686	-8.432	-4.1625	3.61	-0.776	6.974	1.168	6.45
M1C3-D	-4.176	-1.306	-6.902	-2.4925	-0.96	0.934	8.144	3.538	8
M1C3-D	-4.636	-6.056	-9.142	-5.0225	1.12	-1.286	6.994	1.378	6.34
M1C3-D	-10.343	-4.996	-8.712	-5.4225	3.45	-1.006	6.354	1.748	6.82
M1C3-D	-5.82	-2.816	-6.582	-3.0825	-0.68	-0.056	-4.0926	4.048	8.8

Step. 3

Relative Fold (= 2^(-ΔΔCt))

	TNF-α	IL-1β	IL-6	TSG-6	IL-1ra	iNOS	AGG	COLI	COLII
Control	1.22774	0.17629	0.98078	1.23328	1.14076	0.89255	0.85619	1.09581	0.79004
Control	1.66555	9.10084	0.98078	1.4871	1.71713	1.21083	1.16959	0.96059	1.15669
Control	0.76631	0.70028	0.99447	0.5481	0.08657	1.02527	1.04681	0.73306	1.07923
Control	0.405	3.30809	0.93433			1.16959	0.90501	0.9876	0.99309
Control	1.57571	0.26906	1.11884	0.99481	5.89708	0.77164	1.05409	1.31221	1.02101
LPS	1.48041	2.59548	38.6394			3.24001	0.25279		0.23651
LPS	22.7217	9.16414	26.7599	1.83083	2.37841	2.55974	0.27855	0.52924	0.21764
LPS	15.736	14.0841	53.5198	0.96761	0.04067	2.98142	0.24249	0.86574	0.22376
LPS	7.39218	10.5268	32.4917	3.53694	4.50023	3.47256	0.22006	0.90878	0.21022
LPS	2.12285	10.0283	43.1713	4.50804	0.85263	3.82643	0.37268	1.30315	0.25882
M1C3-INI	2.50707	15.6273	40.5604	4.57097	0.16267	2.43851	0.48163	0.98078	0.36098
M1C3-INI	2.63171	13.5104	48.908	1.15069	0.28917	2.50707	0.59296	0.33728	0.50348
M1C3-INI	0.78785		45.6328		0.05791	2.98142	0.41639		0.37113
M1C3-INI	2.89989	16.0667	46.2698	2.48372		2.7057	0.54943	1.40639	0.43528
M1C3-INI	2.09362	4.84333	52.4183	1.2079	0.27168	2.66845	0.46522	0.92787	0.44135
M1C3-D	41.2408	9.8902	452.571	30.5373	24.59	1.72429	0.01292	0.71301	0.01924
M1C3-D	13.141	12.8705	345.37	17.9076	0.0819	1.71238	0.00795	0.44504	0.01144

LPS	1.1969	1.14873	1.72851	-0.0143	-1.3908	0.47442	-0.6153	-0.0626	-0.6502
LPS	0.86877	1.0223	1.51177	0.54863	0.65324	0.54065	-0.6574	-0.0415	-0.6773
LPS	0.32692	1.00123	1.63519	0.65399	-0.0692	0.58279	-0.4287	0.11499	-0.587
M1C3-INI	0.39917	1.19388	1.6081	0.66001	-0.7887	0.38712	-0.3173	-0.0084	-0.4425
M1C3-INI	0.42024	1.13067	1.68938	0.06096	-0.5388	0.39917	-0.227	-0.472	-0.298
M1C3-INI	-0.1036		1.65928		-1.2372	0.47442	-0.3805		-0.4305
M1C3-INI	0.46238	1.20593	1.6653	0.3951		0.43228	-0.2601	0.14811	-0.3612
M1C3-INI	0.3209	0.68514	1.71948	0.08203	-0.5659	0.42626	-0.3323	-0.0325	-0.3552
M1C3-D	1.61533	0.99521	2.65569	1.48483	1.39076	0.23661	-1.8887	-0.1469	-1.7159
M1C3-D	1.11863	1.1096	2.53828	1.25304	-1.0867	0.2336	-2.0994	-0.3516	-1.9416
M1C3-D	1.2571	0.39315	2.07771	0.75032	0.28899	-0.2812	-2.4516	-1.065	-2.4082
M1C3-D	1.39558	1.82304	2.75202	1.51192	-0.3372	0.38712	-2.1054	-0.4148	-1.9085
M1C3-D	3.11342	1.50395	2.62257	1.63234	-1.0386	0.30284	-1.9127	-0.5262	-2.053
M1C3-D	1.75199	0.8477	1.98138	0.92792	0.2047	0.01686	1.23198	-1.2186	-2.6491

Step. 5

Ave.

	TNF-α	IL-1β	IL-6	TSG-6	IL-1ra	iNOS	AGG	COLI	COLII
Control	8.9E-17	0	-1E-16	0	0	-4E-17	4.5E-16	4.3E-16	-4E-16
LPS	0.78388	0.90971	1.578	0.36274	-0.1076	0.50332	-0.5708	-0.0664	-0.6406
M1C3-INI	0.29983	1.05391	1.66831	0.29952	-0.7827	0.42385	-0.3034	-0.0912	-0.3775
M1C3-D	1.70867	1.11211	2.43794	1.26006	-0.0963	0.14931	-1.5376	-0.6205	-2.1127

std.

	TNF-α	IL-1β	IL-6	TSG-6	IL-1ra	iNOS	AGG	COLI	COLII
Control	0.25665	0.72577	0.02914	0.18814	0.7706	0.08179	0.05471	0.09223	0.06257
LPS	0.52221	0.28567	0.11516	0.30086	0.90572	0.06642	0.08752	0.16084	0.03532
M1C1	0.23127	0.24805	0.04117	0.28478	0.32305	0.03388	0.0606	0.2662	0.0594
M1C3	0.72581	0.50189	0.32509	0.35292	0.93565	0.24401	1.37171	0.42499	0.34843

M1C3-D	18.076	2.47255	119.594	5.62752	1.94531	0.52341	0.00354	0.08609	0.00391
M1C3-D	24.8642	66.5331	564.958	32.503	0.46009	2.43851	0.00785	0.38475	0.01234
M1C3-D	1298.44	31.9114	419.347	42.8879	0.09151	2.00834	0.01223	0.29771	0.00885
M1C3-D	56.493	7.04207	95.8031	8.47081	1.60214	1.03958	17.0601	0.06045	0.00224

Inflammation Related Genes_72 hr

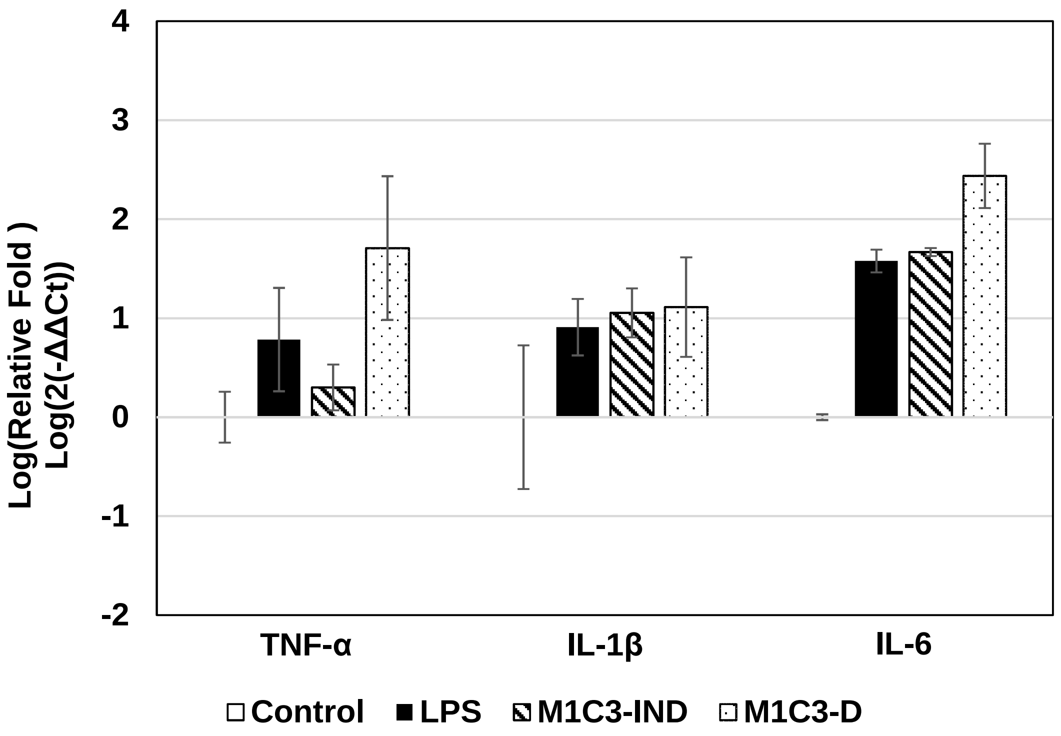

Anti-Inflammation Related Genes_72 hr

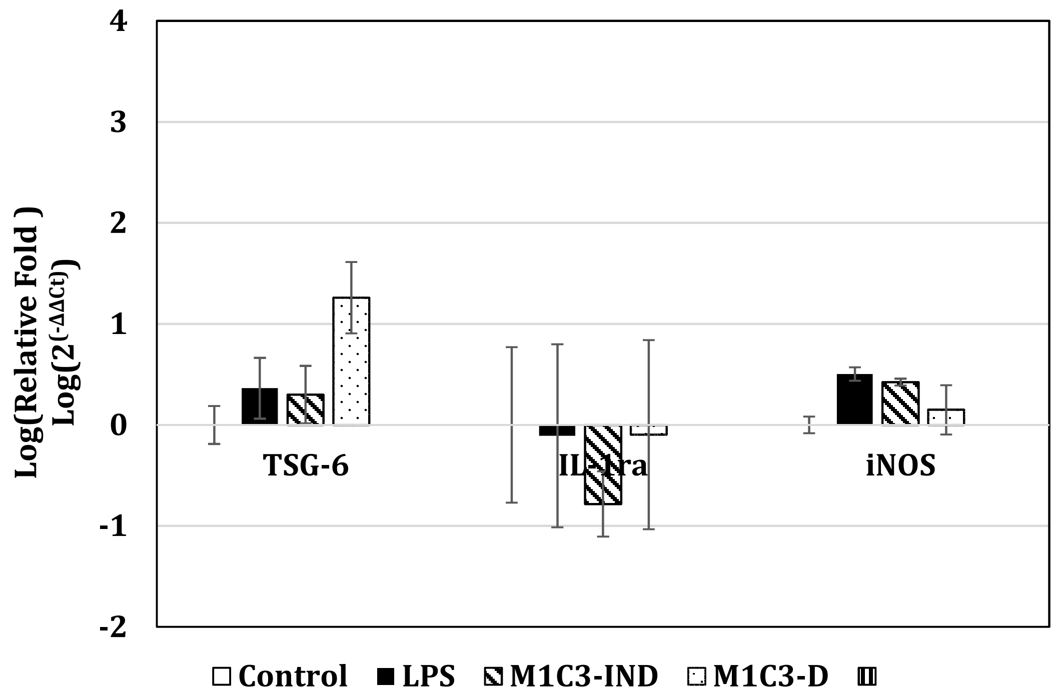

ECM Related Genes_72 hr

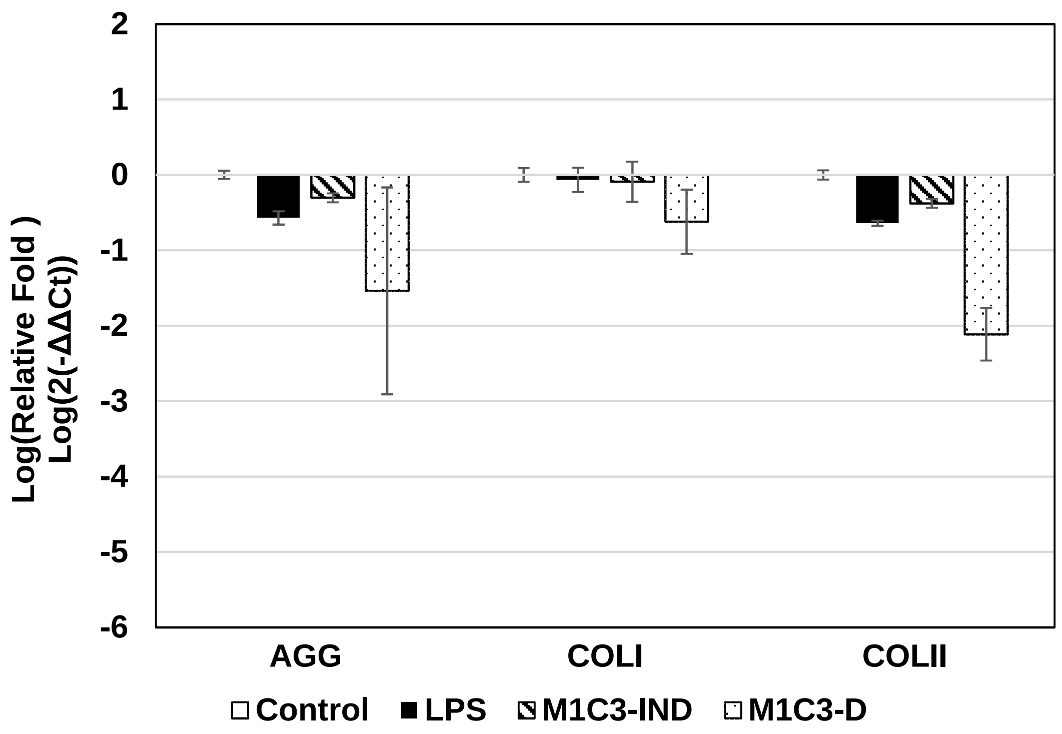

Supplement: S6 Data — (PDF) [file pone.0205563.s006.pdf]
