# Supplementary material for: Can mesenchymal stem cells and their conditioned medium assist inflammatory chondrocytes recovery?
Source: PLoS One. 2018 Nov 21;13(11):e0205563. doi: 10.1371/journal.pone.0205563 (PMC6248915; doi:10.1371/journal.pone.0205563)

Figure 6. Gene expression MSC-conditioned medium in chondrocytes with LPS-induced inflammation
Evaluation Time Point: 24 hr.

Ct number

	TNF-α	IL-1β	IL-6	iNOS	AGG	COLII	GAPDH		TSG-6	IL-1ra	Col I	GAPDH
Control	30.45	34.66	30.05	32.1	28.28	36.16	34.83	Control	39.97	47.7	14.78	34.82
Control	31.93	49.07	28.24	32.11	26.88	34.9	34.04	Control	36.14	42.14	13.27	34.15
Control	30.8	50	31.61	32.75	29.53	38.16	35.13	Control	41.74	50	16.15	35.24
Control	31.6	33.9	28.62	32.74	26.25	34.82	33.74	Control	37.84	50	12.8	33.76
LPS	20.73	24.03	16.81	21.66	30.22	36.34	34.22	LPS	26.77	27.75	14.24	33.9
LPS	20.16	23.63	15.71	21.23	30.23	35.93	33.8	LPS	26.69	27.31	14.05	33.49
LPS	21.04	24.76	16.72	22.07	30.75	36.69	34.12	LPS	27.63	28.17	14.5	34.9
LPS	21.14	25.01	16.92	22.22	30.35	36.53	32.67	LPS	27.24	27.83	14.05	34.3
PT5X	22.57	26.34	18.51	23.08	32.31	38.58	35.52	PT5X	29.93	29.56	16.19	35.79
PT5X	24.76	29.36	21.07	25.29	35.16	41.31	35.49	PT5X	34.72	31.76	18.62	37.63
PT5X	22.32	27.48	18.62	23.71	32.22	38.29	35.03	PT5X	28.97	29.27	15.91	34.12
PT5X	24.98	30.22	21.92	25.38	37.24	43.63	36.76	PT5X	35.92	31.81	19.08	32.5
CM1X	20.82	25.29	17.6	22.13	30.95	37.51	34.22	CM1X	27.53	27.88	14.31	34.66
CM1X	20.53	26.19	17.25	22.52	31.04	38.02	34.32	CM1X	27.36	27.28	14.05	33.95
CM1X	20.96	25.9	17.51	22.35	30.83	37.53	34.17	CM1X	26.92	28.2	14.5	34.53
CM1X	22.12	27.79	18.59	23.7	32.32	39.33	35.37	CM1X	29.04	29.12	15.86	35.18
CM5X	24.47	29.7	20.54	25.71	29.6	37.26	32.93	CM5X	27.76	31.46	13.52	33.16
CM5X	24.68	30.89	20.37	25.78	29.84	37.31	32.93	CM5X	27.77	30.92	13.76	33.13
CM5X	24.57	30.82	20.8	25.95	30.33	37.88	32.89	CM5X	29.06	31.1	14	33.7
CM5X	25.31	31.17	21.52	26.18	31.73	39.68	34.19	CM5X	31.65	32.16	14.67	34.25
CM10X	25.65	32.35	20.25	27.45	29.25	36.62	32.27	CM10X	27.16	34.67	13.47	32.31
CM10X	27.24	33.61	21.04	29.27	29.62	37.52	32.22	CM10X	28.78	34.95	13.81	32.85
CM10X	27.06	31.5	20.47	27.17	29.82	37.57	32.79	CM10X	28.4	35.03	14.18	33.61
CM10X	27.27	34.55	21.06	29.9	30.03	36.87	32.52	CM10X	28.75	42.27	13.8	32.99

Step.1
ΔCt number (=Target gene Ct- GAPDH Ct)

	TNF-α	IL-1β	IL-6	TSG-6	IL-1ra	iNOS	AGG	COLI	COLII
Control	-4.38	-0.17	-4.78	5.15	12.88	-2.73	-6.55	-20.04	1.33
Control	-2.11	15.03	-5.8	1.99	7.99	-1.93	-7.16	-20.88	0.86
Control	-4.33	14.87	-3.52	6.5	14.76	-2.38	-5.6	-19.09	3.03
Control	-2.14	0.16	-5.12	4.08	16.24	-1	-7.49	-20.96	1.08
LPS	-13.49	-10.19	-17.41	-7.13	-6.15	-12.56	-4	-19.66	2.12
LPS	-13.64	-10.17	-18.09	-6.8	-6.18	-12.57	-3.57	-19.44	2.13
LPS	-13.08	-9.36	-17.4	-7.27	-6.73	-12.05	-3.37	-20.4	2.57
LPS	-11.53	-7.66	-15.75	-7.06	-6.47	-10.45	-2.32	-20.25	3.86
PT5X	-12.95	-9.18	-17.01	-5.86	-6.23	-12.44	-3.21	-19.6	3.06
PT5X	-10.73	-6.13	-14.42	-2.91	-5.87	-10.2	-0.33	-19.01	5.82
PT5X	-12.71	-7.55	-16.41	-5.15	-4.85	-11.32	-2.81	-18.21	3.26
PT5X	-11.78	-6.54	-14.84	3.42	-0.69	-11.38	0.48	-13.42	6.87
CM1X	-13.4	-8.93	-16.62	-7.13	-6.78	-12.09	-3.27	-20.35	3.29
CM1X	-13.79	-8.13	-17.07	-6.59	-6.67	-11.8	-3.28	-19.9	3.7

Control ΔCt

	TNF-α	IL-1β	IL-6	TSG-6	IL-1ra	iNOS	AGG	COLI	COLII
	-4.38	-0.17	-4.78	5.15	12.88	-2.73	-6.55	-20.04	1.33
	-2.11	15.03	-5.8	1.99	7.99	-1.93	-7.16	-20.88	0.86
	-4.33	14.87	-3.52	6.5	14.76	-2.38	-5.6	-19.09	3.03
	-2.14	0.16	-5.12	4.08	16.24	-1	-7.49	-20.96	1.08
Ave.	-3.24	7.4725	-4.805	4.43	12.9675	-2.01	-6.7	-20.243	1.575
std.	1.11519	7.47862	0.82781	1.6492	3.11069	0.64842	0.71906	0.75672	0.85634

Step. 4
Log(Relative Fold (= 2^(-ΔΔCt)))

	TNF-α	IL-1β	IL-6	TSG-6	IL-1ra	iNOS	AGG	COLI	COLII
Control	0.34317	2.30062	-0.0075	-0.2167	0.02634	0.21674	-0.0452	-0.061	0.07375
Control	-0.3402	-2.275	0.29952	0.73451	1.49838	-0.0241	0.13847	0.19191	0.21524
Control	0.32812	-2.2269	-0.3868	-0.6231	-0.5396	0.11138	-0.3311	-0.3469	-0.438
Control	-0.3311	2.20128	0.09482	0.10536	-0.9851	-0.304	0.23781	0.21599	0.14901

CM1X	-13.21	-8.27	-16.66	-7.61	-6.33	-11.82	-3.34	-20.03	3.36
CM1X	-13.25	-7.58	-16.78	-6.14	-6.06	-11.67	-3.05	-19.32	3.96
CM5X	-8.46	-3.23	-12.39	-5.4	-1.7	-7.22	-3.33	-19.64	4.33
CM5X	-8.25	-2.04	-12.56	-5.36	-2.21	-7.15	-3.09	-19.37	4.38
CM5X	-8.32	-2.07	-12.09	-4.64	-2.6	-6.94	-2.56	-19.7	4.99
CM5X	-8.88	-3.02	-12.67	-2.6	-2.09	-8.01	-2.46	-19.58	5.49
CM10X	-6.62	0.08	-12.02	-5.15	2.36	-4.82	-3.02	-18.84	4.35
CM10X	-4.98	1.39	-11.18	-4.07	2.1	-2.95	-2.6	-19.04	5.3
CM10X	-5.73	-1.29	-12.32	-5.21	1.42	-5.62	-2.97	-19.43	4.78
CM10X	-5.25	2.03	-11.46	-4.24	9.28	-2.62	-2.49	-19.19	4.35

Step. 2

ΔΔCt (=Experimal ΔCt- Control ΔCt)

	TNF-α	IL-1β	IL-6	TSG-6	IL-1ra	iNOS	AGG	COLI	COLII
Control	-1.14	-7.6425	0.025	0.72	-0.0875	-0.72	0.15	0.2025	-0.245
Control	1.13	7.5575	-0.995	-2.44	-4.9775	0.08	-0.46	-0.6375	-0.715
Control	-1.09	7.3975	1.285	2.07	1.7925	-0.37	1.1	1.1525	1.455
Control	1.1	-7.3125	-0.315	-0.35	3.2725	1.01	-0.79	-0.7175	-0.495
LPS	-10.25	-17.663	-12.605	-11.56	-19.118	-10.55	2.7	0.5825	0.545
LPS	-10.4	-17.643	-13.285	-11.23	-19.148	-10.56	3.13	0.8025	0.555
LPS	-9.84	-16.833	-12.595	-11.7	-19.698	-10.04	3.33	-0.1575	0.995
LPS	-8.29	-15.133	-10.945	-11.49	-19.438	-8.44	4.38	-0.0075	2.285
PT5X	-9.71	-16.653	-12.205	-10.29	-19.198	-10.43	3.49	0.6425	1.485
PT5X	-7.49	-13.603	-9.615	-7.34	-18.838	-8.19	6.37	1.2325	4.245
PT5X	-9.47	-15.023	-11.605	-9.58	-17.818	-9.31	3.89	2.0325	1.685
PT5X	-8.54	-14.013	-10.035	-1.01	-13.658	-9.37	7.18	6.8225	5.295
CM1X	-10.16	-16.403	-11.815	-11.56	-19.748	-10.08	3.43	-0.1075	1.715
CM1X	-10.55	-15.603	-12.265	-11.02	-19.638	-9.79	3.42	0.3425	2.125
CM1X	-9.97	-15.743	-11.855	-12.04	-19.298	-9.81	3.36	0.2125	1.785
CM1X	-10.01	-15.053	-11.975	-10.57	-19.028	-9.66	3.65	0.9225	2.385
CM5X	-5.22	-10.703	-7.585	-9.83	-14.668	-5.21	3.37	0.6025	2.755
CM5X	-5.01	-9.5125	-7.755	-9.79	-15.178	-5.14	3.61	0.8725	2.805
CM5X	-5.08	-9.5425	-7.285	-9.07	-15.568	-4.93	4.14	0.5425	3.415
CM5X	-5.64	-10.493	-7.865	-7.03	-15.058	-6	4.24	0.6625	3.915
CM10X	-3.38	-7.3925	-7.215	-9.58	-10.608	-2.81	3.68	1.4025	2.775
CM10X	-1.74	-6.0825	-6.375	-8.5	-10.868	-0.94	4.1	1.2025	3.725
CM10X	-2.49	-8.7625	-7.515	-9.64	-11.548	-3.61	3.73	0.8125	3.205
CM10X	-2.01	-5.4425	-6.655	-8.67	-3.6875	-0.61	4.21	1.0525	2.775

Step. 3

Relative Fold (= 2^(-ΔΔCt))

	TNF-α	IL-1β	IL-6	TSG-6	IL-1ra	iNOS	AGG	COLI	COLII
Control	2.20381	199.812	0.98282	0.6071	1.06253	1.64718	0.90125	0.86904	1.18509
Control	0.45692	0.00531	1.99308	5.42642	31.5048	0.94606	1.37554	1.55563	1.64148
Control	2.12874	0.00593	0.41037	0.23816	0.28867	1.29235	0.46652	0.44985	0.36476
Control	0.46652	158.958	1.24401	1.27456	0.10349	0.49655	1.72907	1.64433	1.40932
LPS	1217.75	207464	6229.93	3019.3	568776	1499.22	0.15389	0.66781	0.68539
LPS	1351.18	204608	9981.22	2401.97	580727	1509.65	0.11423	0.57335	0.68066
LPS	916.506	116704	6186.9	3326.99	850234	1052.79	0.09944	1.11535	0.50174

LPS	3.08556	5.31694	3.79448	3.47991	5.75494	3.17587	-0.8128	-0.1753	-0.1641
LPS	3.13071	5.31092	3.99918	3.38057	5.76397	3.17888	-0.9422	-0.2416	-0.1671
LPS	2.96214	5.06709	3.79147	3.52205	5.92954	3.02234	-1.0024	0.04741	-0.2995
LPS	2.49554	4.55534	3.29477	3.45883	5.85127	2.54069	-1.3185	0.00226	-0.6879
PT5X	2.923	5.0129	3.67407	3.0976	5.77902	3.13974	-1.0506	-0.1934	-0.447
PT5X	2.25471	4.09476	2.8944	2.20956	5.67065	2.46544	-1.9176	-0.371	-1.2779
PT5X	2.85075	4.52222	3.49345	2.88387	5.3636	2.80259	-1.171	-0.6118	-0.5072
PT5X	2.5708	4.21818	3.02084	0.30404	4.11132	2.82065	-2.1614	-2.0538	-1.594
CM1X	3.05846	4.93764	3.55667	3.47991	5.94459	3.03438	-1.0325	0.03236	-0.5163
CM1X	3.17587	4.69682	3.69213	3.31735	5.91148	2.94708	-1.0295	-0.1031	-0.6397
CM1X	3.00127	4.73896	3.56871	3.6244	5.80913	2.9531	-1.0115	-0.064	-0.5373
CM1X	3.01331	4.53125	3.60483	3.18189	5.72785	2.90795	-1.0988	-0.2777	-0.718
CM5X	1.57138	3.22177	2.28331	2.95912	4.41536	1.56837	-1.0145	-0.1814	-0.8293
CM5X	1.50816	2.86355	2.33449	2.94708	4.56888	1.54729	-1.0867	-0.2626	-0.8444
CM5X	1.52923	2.87258	2.193	2.73034	4.68628	1.48408	-1.2463	-0.1633	-1.028
CM5X	1.69781	3.15856	2.3676	2.11624	4.53276	1.80618	-1.2764	-0.1994	-1.1785
CM10X	1.01748	2.22536	2.17193	2.88387	3.19318	0.84589	-1.1078	-0.4222	-0.8354
CM10X	0.52379	1.83101	1.91907	2.55875	3.27144	0.28297	-1.2342	-0.362	-1.1213
CM10X	0.74956	2.63778	2.26224	2.90193	3.47614	1.08672	-1.1228	-0.2446	-0.9648
CM10X	0.60507	1.63836	2.00335	2.60993	1.11005	0.18363	-1.2673	-0.3168	-0.8354

Step. 5

Ave.

	TNF-α	IL-1β	IL-6	TSG-6	IL-1ra	iNOS	AGG	COLI	COLII
Control	0	0	0	-3E-17	0	0	0	0	0
LPS	2.91849	5.06257	3.71998	3.46034	5.82493	2.97944	-1.019	-0.0918	-0.3296
PT5X	2.64982	4.46202	3.27069	2.12377	5.23115	2.8071	-1.5751	-0.8075	-0.9565
CM1X	3.06223	4.72617	3.60559	3.40089	5.84826	2.96063	-1.0431	-0.1031	-0.6028
CM5X	1.57664	3.02911	2.2946	2.6882	4.55082	1.60148	-1.156	-0.2017	-0.9701
CM10X	0.72398	2.08313	2.08915	2.73862	2.7627	0.5998	-1.183	-0.3364	-0.9392

std.

	TNF-α	IL-1β	IL-6	TSG-6	IL-1ra	iNOS	AGG	COLI	COLII
Control	0.38764	2.59957	0.28775	0.57326	1.08127	0.22539	0.24995	0.26303	0.29766
LPS	0.29083	0.35763	0.29968	0.05932	0.08216	0.30149	0.21479	0.13861	0.24703
PT5X	0.30406	0.40883	0.37254	1.27081	0.76701	0.27543	0.54755	0.84835	0.56892
CM1X	0.07966	0.16707	0.06122	0.19248	0.09884	0.05309	0.03828	0.12957	0.0938
CM5X	0.08494	0.18778	0.07609	0.39553	0.11158	0.14109	0.12579	0.04323	0.16575
CM10X	0.2168	0.44319	0.1561	0.17952	1.10821	0.43636	0.07961	0.0749	0.13589

LPS	312.996	35920	1971.39	2876.3	710020	347.291	0.04803	1.00521	0.20519
PT5X	837.532	103015	4721.4	1251.98	601206	1379.57	0.089	0.6406	0.35725
PT5X	179.769	12438.3	784.158	162.017	468438	292.036	0.01209	0.42558	0.05274
PT5X	709.176	33283	3114.96	765.363	230995	634.73	0.06745	0.24443	0.311
PT5X	372.217	16526.6	1049.15	2.01391	12921.6	661.685	0.0069	0.00884	0.02547
CM1X	1144.1	86625.3	3603.04	3019.3	880217	1082.39	0.09278	1.07736	0.3046
CM1X	1499.22	49753.1	4921.9	2076.59	815599	885.286	0.09343	0.78867	0.22925
CM1X	1002.93	54823.2	3704.34	4211.15	644357	897.644	0.0974	0.86304	0.29018
CM1X	1031.12	33982.4	4025.63	1520.15	534378	809.002	0.07966	0.52759	0.19144
CM5X	37.2715	1666.38	192.005	910.175	26023	37.014	0.09672	0.65861	0.14814
CM5X	32.2226	730.378	216.017	885.286	37058.1	35.261	0.0819	0.5462	0.14309
CM5X	33.8246	745.725	155.957	537.455	48560.6	30.4844	0.05672	0.68658	0.09375
CM5X	49.8665	1440.65	233.131	130.69	34100.4	64	0.05292	0.63178	0.06629
CM10X	10.4107	168.021	148.57	765.363	1560.18	7.01285	0.07802	0.37827	0.1461
CM10X	3.34035	67.7665	82.9977	362.039	1868.29	1.91853	0.05831	0.43452	0.07562
CM10X	5.61778	434.286	182.911	797.865	2993.26	12.2101	0.07536	0.56939	0.10844
CM10X	4.02782	43.4866	100.775	407.315	12.8839	1.52626	0.05403	0.48213	0.1461

Inflammation Related Genes_24 hr

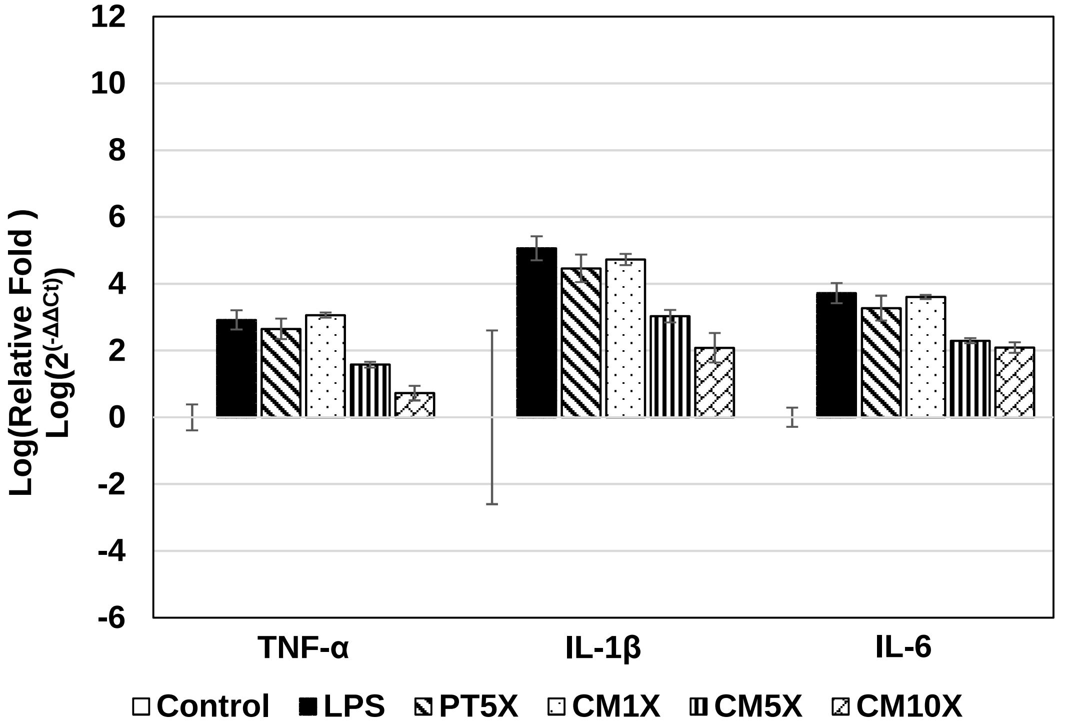

Anti-Inflammation Related Genes_24 hr

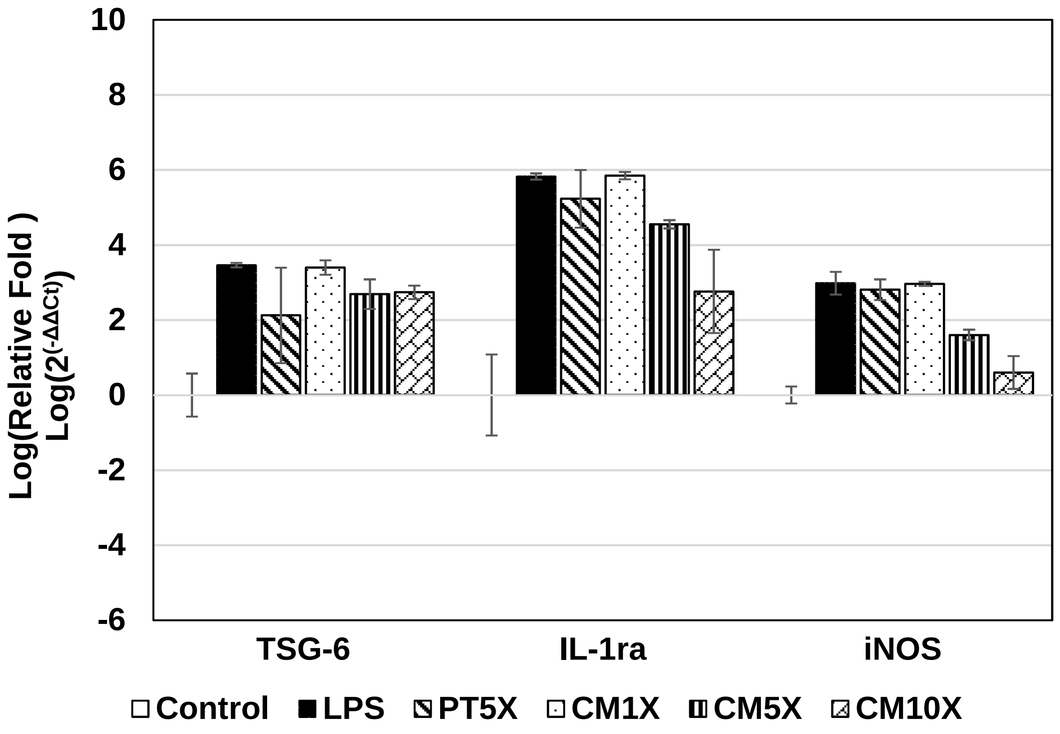

ECM Related Genes_24 hr

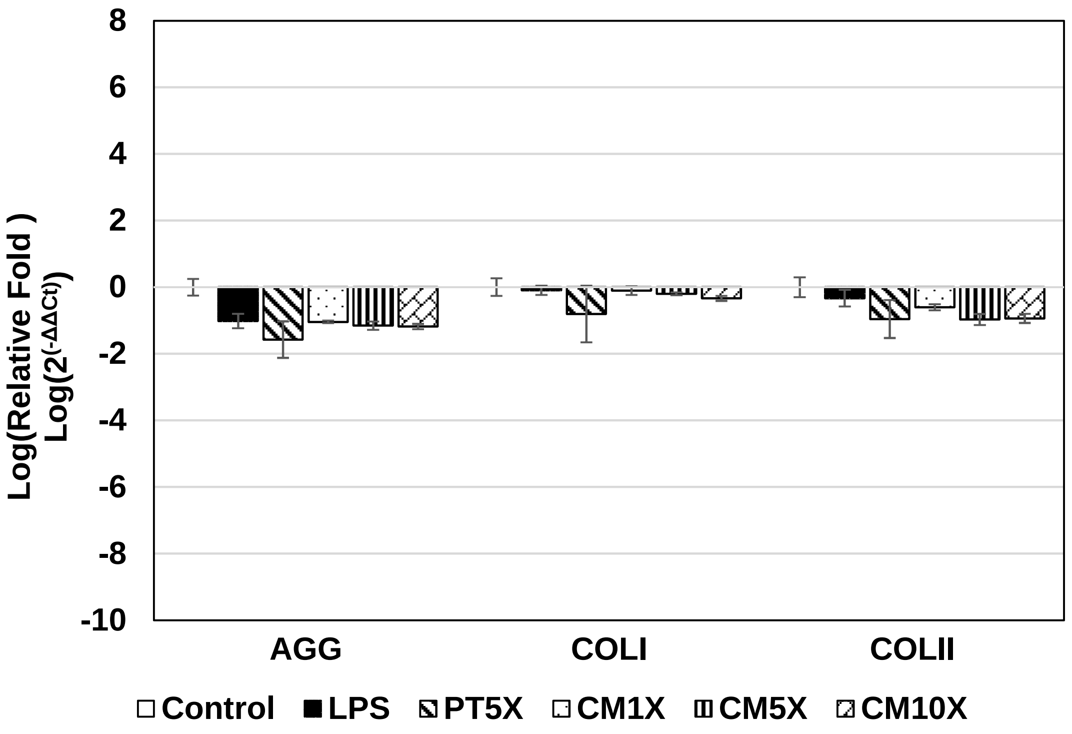

Supplement: S7 Data — (PDF) [file pone.0205563.s007.pdf]
