# Supplementary material for: Can mesenchymal stem cells and their conditioned medium assist inflammatory chondrocytes recovery?
Source: PLoS One. 2018 Nov 21;13(11):e0205563. doi: 10.1371/journal.pone.0205563 (PMC6248915; doi:10.1371/journal.pone.0205563)

Figure 6. Gene expression MSC-conditioned medium in chondrocytes with LPS-induced inflammation
Evaluation Time Point: 72 hr.

Ct number

	TNF-α	IL-1β	IL-6	iNOS	AGG	COLII	GAPDH
Control	32.5	34.76	28.23	31.06	21.96	28.06	34.92
Control	31.95	34.89	27.75	32.18	22.12	27.51	34.85
Control	31.43	33.66	27.76	32.1	21.78	27.2	35.08
Control	32.95	35.27	27.69	31.92	21.61	27.2	34.37
LPS	25.66	19.81	16.77	23.23	29.77	33.07	32.78
LPS	24.61	19.11	15.79	22.62	28.87	31.69	32.85
LPS	25.13	19.93	16.12	22.88	29.01	32.17	32.63
LPS	25.08	19.76	15.81	22.91	29.24	31.62	32.92
PT5X	25.06	20.13	15.8	21.75	27.59	31.89	32.55
PT5X	25.01	20.06	15.8	21.92	27.69	31.53	33.04
PT5X	24.84	20.45	15.93	21.37	26.66	31.28	32.89
PT5X	24.8	19.67	15.46	21.32	27.22	31.16	32.64
CM1X	25.53	20.14	16.13	22.62	29.22	31.9	32.93
CM1X	25.56	20.36	16.33	22.85	29.66	32.74	33.27
CM1X	25.36	19.51	16.1	22.96	29.14	32.82	33.21
CM1X	24.81	19.34	15.54	22.08	28.47	31.46	32.57
CM5X	23.76	20.49	15.2	22.99	27.35	31.62	32.57
CM5X	23.78	21.11	15.22	23.45	27.3	31.49	32
CM5X	24.61	21.53	15.6	23.71	27.32	31.69	32.25
CM5X	24.63	22.02	15.45	24.44	27.17	31.59	32.21
CM10X	27.44	27.83	18.67	27.36	26.89	32.52	32.5
CM10X	27.18	26.91	18.31	26.89	26.75	32.22	32.38
CM10X	28.64	29.36	19.22	27.18	26.67	32.22	32.31
CM10X	27.09	28.86	19.14	27.96	26.71	32.76	32.74

	TSG-6	IL-1ra	Col I	GAPDH
Control	36.51	34.9	13.31	34.92
Control	36.53	34.88	13.19	34.85
Control	35.73	35.92	12.71	35.08
Control	35.5		13.21	34.37
LPS	25.77	34.91	17.91	32.78
LPS	24.97	35.94	17.14	32.85
LPS	25.52	35.85	17.71	32.63
LPS	25.85	42.25	17.69	32.92
PT5X	25.74	34.36	17.78	32.55
PT5X	25.74	34.96	17.72	33.04
PT5X	25.67	34.51	17.53	32.89
PT5X	25.35	34.93	16.82	32.64
CM1X	26.47	35.89	17.84	32.93
CM1X	26.7	36.09	18.71	33.27
CM1X	26.71	34.86	17.99	33.21
CM1X	26.16	34.92	17.48	32.57
CM5X	25.35	37.44	15.73	32.57
CM5X	25.68	34.8	15.28	32
CM5X	26.07	35.07	15.5	32.25
CM5X	26.27	35.01	15.36	32.21
CM10X	27.07	35.84	14.64	32.5
CM10X	26.34	39.3	14.25	32.38
CM10X	27.18	47.6	14.22	32.31
CM10X	27.82	50	14.11	32.74

Step.1

ΔCt number (=Target gene Ct- GAPDH Ct)

	TNF-α	IL-1β	IL-6	TSG-6	IL-1ra	iNOS	AGG	COLI	COLII
Control	-2.42	-0.16	-6.69	1.59	-0.02	-3.86	-12.96	-21.61	-6.86
Control	-2.9	0.04	-7.1	1.68	0.03	-2.67	-12.73	-21.66	-7.34
Control	-3.65	-1.42	-7.32	0.65	0.84	-2.98	-13.3	-22.37	-7.88
Control	-1.42	0.9	-6.68	1.13		-2.45	-12.76	-21.16	-7.17
LPS	-7.12	-12.97	-16.01	-7.01	2.13	-9.55	-3.01	-14.87	0.29
LPS	-8.24	-13.74	-17.06	-7.88	3.09	-10.23	-3.98	-15.71	-1.16
LPS	-7.5	-12.7	-16.51	-7.11	3.22	-9.75	-3.62	-14.92	-0.46
LPS	-7.84	-13.16	-17.11	-7.07	9.33	-10.01	-3.68	-15.23	-1.3
PT5X	-7.49	-12.42	-16.75	-6.81	1.81	-10.8	-4.96	-14.77	-0.66
PT5X	-8.03	-12.98	-17.24	-7.3	1.92	-11.12	-5.35	-15.32	-1.51
PT5X	-8.05	-12.44	-16.96	-7.22	1.62	-11.52	-6.23	-15.36	-1.61
PT5X	-7.84	-12.97	-17.18	-7.29	2.29	-11.32	-5.42	-15.82	-1.48
CM1X	-7.4	-12.79	-16.8	-6.46	2.96	-10.31	-3.71	-15.09	-1.03
CM1X	-7.71	-12.91	-16.94	-6.57	2.82	-10.42	-3.61	-14.56	-0.53

Control ΔCt

	TNF-α	IL-1β	IL-6	TSG-6	IL-1ra	iNOS	AGG	COLI	COLII
	-2.42	-0.16	-6.69	1.59	-0.02	-3.86	-12.96	-21.61	-6.86
	-2.9	0.04	-7.1	1.68	0.03	-2.67	-12.73	-21.66	-7.34
	-3.65	-1.42	-7.32	0.65	0.84	-2.98	-13.3	-22.37	-7.88
	-1.42	0.9	-6.68	1.13		-2.45	-12.76	-21.16	-7.17
Ave.	-2.5975	-0.16	-6.9475	1.2625	0.28333	-2.99	-12.938	-21.7	-7.3125
std.	0.8089	0.82934	0.2738	0.41057	0.39415	0.53642	0.2272	0.43307	0.37009

Step. 4

Log(Relative Fold (= 2^(-ΔΔCt))

	TNF-α	IL-1β	IL-6	TSG-6	IL-1ra	iNOS	AGG	COLI	COLII
Control	-0.0534	1.1E-15	-0.0775	-0.0986	0.09131	0.2619	0.00677	-0.0271	-0.1362
Control	0.09106	-0.0602	0.04591	-0.1257	0.07626	-0.0963	-0.0625	-0.012	0.00828
Control	0.31683	0.3793	0.11213	0.18438	-0.1676	-0.003	0.10912	0.20169	0.17083
Control	-0.3545	-0.3191	-0.0805	0.03989		-0.1626	-0.0534	-0.1626	-0.0429

CM1X	-7.85	-13.7	-17.11	-6.5	1.65	-10.25	-4.07	-15.22	-0.39
CM1X	-7.76	-13.23	-17.03	-6.41	2.35	-10.49	-4.1	-15.09	-1.11
CM5X	-8.81	-12.08	-17.37	-7.22	4.87	-9.58	-5.22	-16.84	-0.95
CM5X	-8.22	-10.89	-16.78	-6.32	2.8	-8.55	-4.7	-16.72	-0.51
CM5X	-7.64	-10.72	-16.65	-6.18	2.82	-8.54	-4.93	-16.75	-0.56
CM5X	-7.58	-10.19	-16.76	-5.94	2.8	-7.77	-5.04	-16.85	-0.62
CM10X	-5.06	-4.67	-13.83	-5.43	3.34	-5.14	-5.61	-17.86	0.02
CM10X	-5.2	-5.47	-14.07	-6.04	6.92	-5.49	-5.63	-18.13	-0.16
CM10X	-3.67	-2.95	-13.09	-5.13	15.29	-5.13	-5.64	-18.09	-0.09
CM10X	-5.65	-3.88	-13.6	-4.92	17.26	-4.78	-6.03	-18.63	0.02

Step. 2

ΔΔCt (=Experimal ΔCt- Control ΔCt)

	TNF-α	IL-1β	IL-6	TSG-6	IL-1ra	iNOS	AGG	COLI	COLII
Control	0.1775	-4E-15	0.2575	0.3275	-0.3033	-0.87	-0.0225	0.09	0.4525
Control	-0.3025	0.2	-0.1525	0.4175	-0.2533	0.32	0.2075	0.04	-0.0275
Control	-1.0525	-1.26	-0.3725	-0.6125	0.55667	0.01	-0.3625	-0.67	-0.5675
Control	1.1775	1.06	0.2675	-0.1325		0.54	0.1775	0.54	0.1425
LPS	-4.5225	-12.81	-9.0625	-8.2725	1.84667	-6.56	9.9275	6.83	7.6025
LPS	-5.6425	-13.58	-10.113	-9.1425	2.80667	-7.24	8.9575	5.99	6.1525
LPS	-4.9025	-12.54	-9.5625	-8.3725	2.93667	-6.76	9.3175	6.78	6.8525
LPS	-5.2425	-13	-10.163	-8.3325	9.04667	-7.02	9.2575	6.47	6.0125
PT5X	-4.8925	-12.26	-9.8025	-8.0725	1.52667	-7.81	7.9775	6.93	6.6525
PT5X	-5.4325	-12.82	-10.293	-8.5625	1.63667	-8.13	7.5875	6.38	5.8025
PT5X	-5.4525	-12.28	-10.013	-8.4825	1.33667	-8.53	6.7075	6.34	5.7025
PT5X	-5.2425	-12.81	-10.233	-8.5525	2.00667	-8.33	7.5175	5.88	5.8325
CM1X	-4.8025	-12.63	-9.8525	-7.7225	2.67667	-7.32	9.2275	6.61	6.2825
CM1X	-5.1125	-12.75	-9.9925	-7.8325	2.53667	-7.43	9.3275	7.14	6.7825
CM1X	-5.2525	-13.54	-10.163	-7.7625	1.36667	-7.26	8.8675	6.48	6.9225
CM1X	-5.1625	-13.07	-10.083	-7.6725	2.06667	-7.5	8.8375	6.61	6.2025
CM5X	-6.2125	-11.92	-10.423	-8.4825	4.58667	-6.59	7.7175	4.86	6.3625
CM5X	-5.6225	-10.73	-9.8325	-7.5825	2.51667	-5.56	8.2375	4.98	6.8025
CM5X	-5.0425	-10.56	-9.7025	-7.4425	2.53667	-5.55	8.0075	4.95	6.7525
CM5X	-4.9825	-10.03	-9.8125	-7.2025	2.51667	-4.78	7.8975	4.85	6.6925
CM10X	-2.4625	-4.51	-6.8825	-6.6925	3.05667	-2.15	7.3275	3.84	7.3325
CM10X	-2.6025	-5.31	-7.1225	-7.3025	6.63667	-2.5	7.3075	3.57	7.1525
CM10X	-1.0725	-2.79	-6.1425	-6.3925	15.0067	-2.14	7.2975	3.61	7.2225
CM10X	-3.0525	-3.72	-6.6525	-6.1825	16.9767	-1.79	6.9075	3.07	7.3325

Step. 3

Relative Fold (= 2^(-ΔΔCt))

	TNF-α	IL-1β	IL-6	TSG-6	IL-1ra	iNOS	AGG	COLI	COLII
Control	0.88423	1	0.83654	0.79692	1.23399	1.82766	1.01572	0.93952	0.73078
Control	1.23328	0.87055	1.11149	0.74872	1.19196	0.80107	0.86604	0.97265	1.01924
Control	2.07412	2.39496	1.29459	1.52891	0.67987	0.99309	1.28565	1.59107	1.48195
Control	0.44212	0.47963	0.83076	1.09619		0.68777	0.88423	0.68777	0.90595
LPS	22.9831	7181.15	534.668	309.222	0.27803	94.3532	0.00103	0.00879	0.00515
LPS	49.953	12245.8	1107.05	565.154	0.14293	151.167	0.00201	0.01573	0.01406
LPS	29.9088	5955.47	756.135	331.416	0.13061	108.383	0.00157	0.0091	0.00865

LPS	1.36141	3.85619	2.72808	2.49027	-0.5559	1.97476	-2.9885	-2.056	-2.2886
LPS	1.69856	4.08799	3.04417	2.75217	-0.8449	2.17946	-2.6965	-1.8032	-1.8521
LPS	1.4758	3.77492	2.8786	2.52037	-0.884	2.03496	-2.8048	-2.041	-2.0628
LPS	1.57815	3.91339	3.05922	2.50833	-2.7233	2.11323	-2.7868	-1.9477	-1.8099
PT5X	1.47279	3.69063	2.95085	2.43006	-0.4596	2.35104	-2.4015	-2.0861	-2.0026
PT5X	1.63535	3.8592	3.09835	2.57757	-0.4927	2.44737	-2.2841	-1.9206	-1.7467
PT5X	1.64137	3.69665	3.01406	2.55349	-0.4024	2.56779	-2.0192	-1.9085	-1.7166
PT5X	1.57815	3.85619	3.08029	2.57456	-0.6041	2.50758	-2.263	-1.7701	-1.7558
CM1X	1.4457	3.80201	2.9659	2.3247	-0.8058	2.20354	-2.7778	-1.9898	-1.8912
CM1X	1.53902	3.83813	3.00804	2.35782	-0.7636	2.23665	-2.8079	-2.1494	-2.0417
CM1X	1.58116	4.07595	3.05922	2.33675	-0.4114	2.18548	-2.6694	-1.9507	-2.0839
CM1X	1.55407	3.93446	3.03513	2.30965	-0.6221	2.25772	-2.6604	-1.9898	-1.8671
CM5X	1.87015	3.58828	3.13749	2.55349	-1.3807	1.98379	-2.3232	-1.463	-1.9153
CM5X	1.69254	3.23005	2.95988	2.28256	-0.7576	1.67373	-2.4797	-1.4991	-2.0478
CM5X	1.51794	3.17888	2.92074	2.24042	-0.7636	1.67072	-2.4105	-1.4901	-2.0327
CM5X	1.49988	3.01933	2.95386	2.16817	-0.7576	1.43892	-2.3774	-1.46	-2.0146
CM10X	0.74129	1.35765	2.07184	2.01464	-0.9201	0.64721	-2.2058	-1.156	-2.2073
CM10X	0.78343	1.59847	2.14409	2.19827	-1.9978	0.75257	-2.1998	-1.0747	-2.1531
CM10X	0.32285	0.83987	1.84908	1.92433	-4.5175	0.6442	-2.1968	-1.0867	-2.1742
CM10X	0.91889	1.11983	2.0026	1.86112	-5.1105	0.53884	-2.0794	-0.9242	-2.2073

Step. 5

Ave.

	TNF-α	IL-1β	IL-6	TSG-6	IL-1ra	iNOS	AGG	COLI	COLII
Control	0	0	0	2.4E-17	0	0	2.7E-16	0	0
LPS	1.52848	3.90812	2.92752	2.56779	-1.252	2.0756	-2.8191	-1.962	-2.0034
PT5X	1.58191	3.77567	3.03589	2.53392	-0.4897	2.46845	-2.2419	-1.9213	-1.8054
CM1X	1.52998	3.91264	3.01707	2.33223	-0.6507	2.22085	-2.7288	-2.0199	-1.971
CM5X	1.64513	3.25413	2.99299	2.31116	-0.9149	1.69179	-2.3977	-1.4781	-2.0026
CM10X	0.69162	1.22895	2.0169	1.99959	-3.1365	0.64571	-2.1704	-1.0604	-2.1855

std.

	TNF-α	IL-1β	IL-6	TSG-6	IL-1ra	iNOS	AGG	COLI	COLII
Control	0.28117	0.28828	0.09517	0.14271	0.14532	0.18646	0.07897	0.15054	0.12864
LPS	0.14386	0.13269	0.15612	0.12354	0.99171	0.08949	0.12244	0.11621	0.21998
PT5X	0.07813	0.09476	0.06728	0.07006	0.0849	0.09242	0.16052	0.12937	0.13251
CM1X	0.05884	0.12239	0.04001	0.02034	0.17783	0.03246	0.07497	0.08825	0.10785
CM5X	0.17335	0.24016	0.09785	0.16832	0.31058	0.22359	0.06546	0.01951	0.05975
CM10X	0.25725	0.32475	0.12591	0.14667	2.00104	0.08726	0.06082	0.09762	0.02663

LPS	37.8573	8192	1146.09	322.354	0.00189	129.787	0.00163	0.01128	0.01549
PT5X	29.7022	4904.87	892.99	269.194	0.34708	224.411	0.00397	0.0082	0.00994
PT5X	43.1862	7231.1	1254.16	378.067	0.3216	280.139	0.0052	0.01201	0.01792
PT5X	43.7891	4973.34	1032.91	357.674	0.39593	369.646	0.00957	0.01234	0.0192
PT5X	37.8573	7181.15	1203.07	375.456	0.24885	321.795	0.00546	0.01698	0.01755
CM1X	27.9059	6338.83	924.481	211.205	0.1564	159.786	0.00167	0.01024	0.01285
CM1X	34.5952	6888.62	1018.69	227.938	0.17234	172.446	0.00156	0.00709	0.00908
CM1X	38.1206	11910.9	1146.09	217.143	0.38779	153.277	0.00214	0.0112	0.00824
CM1X	35.8152	8599.28	1084.26	204.011	0.23871	181.019	0.00219	0.01024	0.01358
CM5X	74.1564	3875.05	1372.41	357.674	0.04162	96.3358	0.00475	0.03443	0.01215
CM5X	49.2653	1698.45	911.753	191.673	0.17475	47.1766	0.00331	0.03169	0.00896
CM5X	32.9567	1509.65	833.189	173.947	0.17234	46.8507	0.00389	0.03235	0.00927
CM5X	31.6142	1045.52	899.201	147.288	0.17475	27.4741	0.00419	0.03467	0.00967
CM10X	5.51171	22.7848	117.988	103.429	0.12019	4.43828	0.00623	0.06983	0.0062
CM10X	6.07338	39.6706	139.343	157.86	0.01005	5.65685	0.00631	0.0842	0.00703
CM10X	2.10307	6.9163	70.6442	84.0106	3E-05	4.40762	0.00636	0.0819	0.0067
CM10X	8.29648	13.1775	100.601	72.6303	7.8E-06	3.45815	0.00833	0.11908	0.0062

Inflammation Related Genes_72 hr

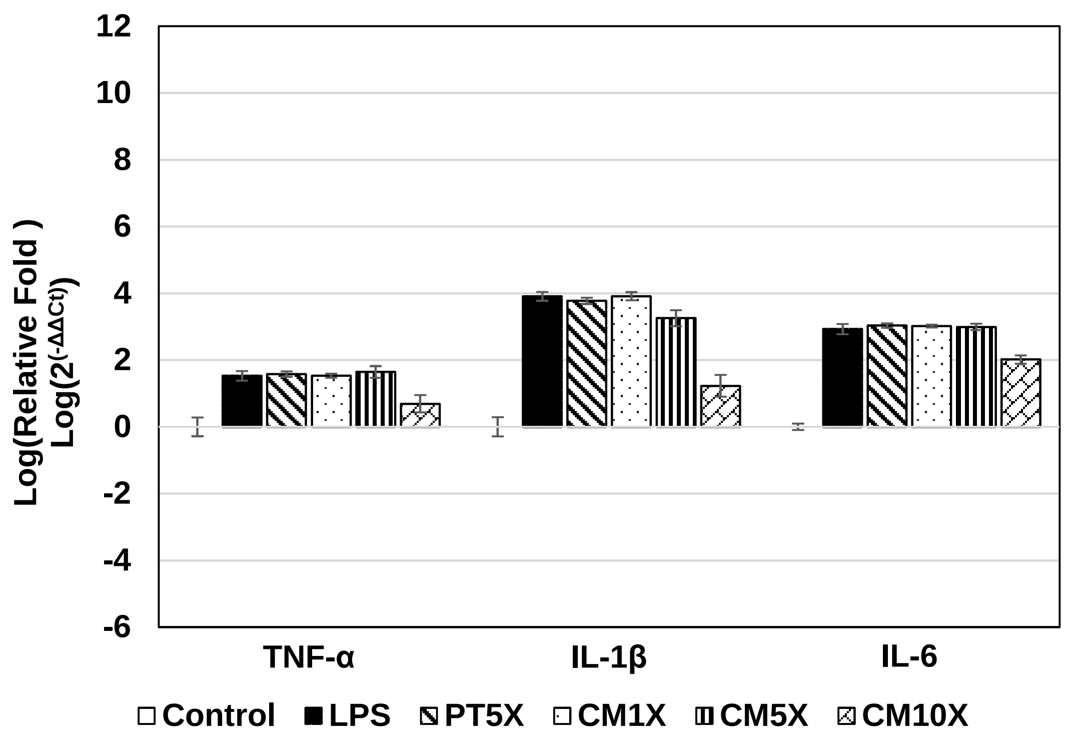

Anti-Inflammation Related Genes_72 hr

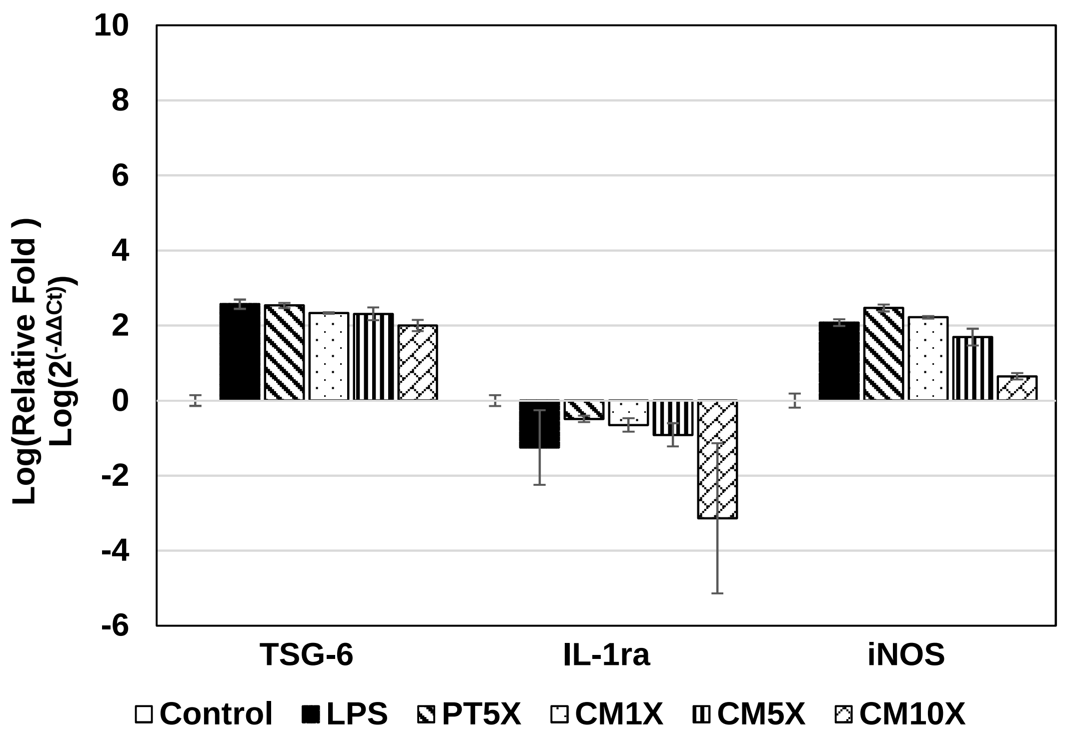

ECM Related Genes_ 72 hr

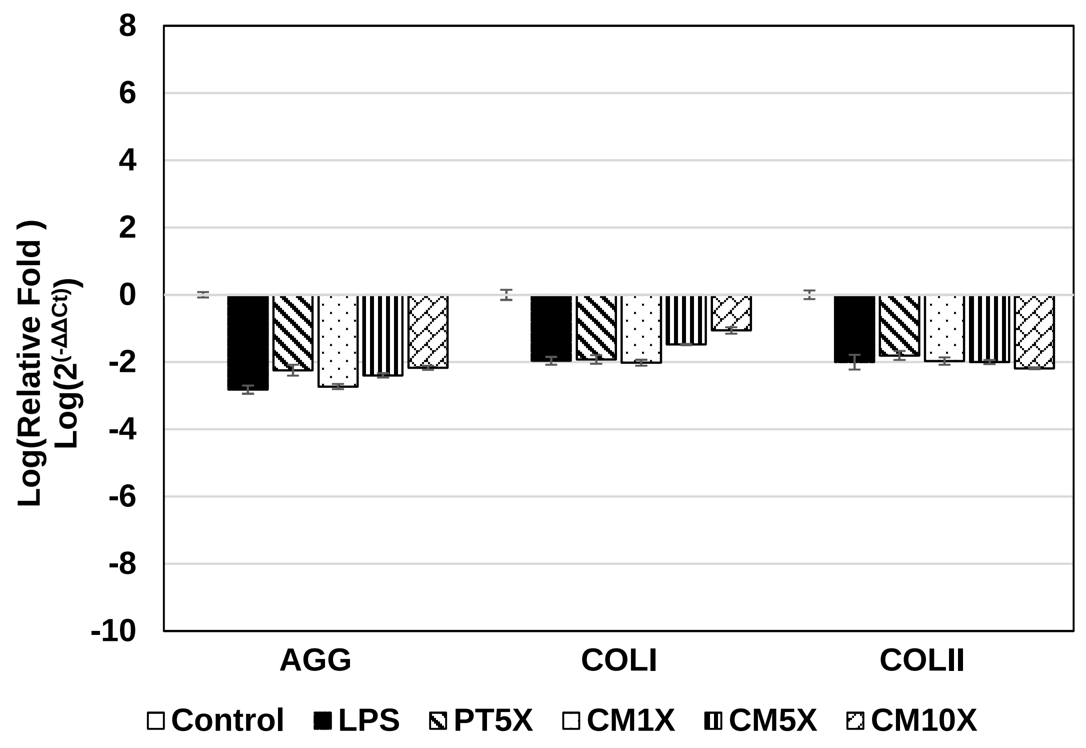

Supplement: S8 Data — (PDF) [file pone.0205563.s008.pdf]
