# Supplementary material for: Can mesenchymal stem cells and their conditioned medium assist inflammatory chondrocytes recovery?
Source: PLoS One. 2018 Nov 21;13(11):e0205563. doi: 10.1371/journal.pone.0205563 (PMC6248915; doi:10.1371/journal.pone.0205563)

Figure 7. Cell viability for chondrocytes incubated with medium or LPS with or without subsequent treatment with different MSC-CM.

24 hr	OD450nm				minues Blank			Ave.	Std.
Control	xLPS, xFBS	0.763	0.767	0.761	0.202	0.206	0.2	0.20267	0.00306
LPS	xFBS	0.717	0.718	0.722	0.156	0.157	0.161	0.158	0.00265
CM1X		0.671	0.686	0.628	0.11	0.125	0.067	0.10067	0.03011
CM5X		0.665	0.703	0.784	0.104	0.142	0.223	0.15633	0.06078
PT5X		0.63	0.649	0.692	0.069	0.088	0.131	0.096	0.03176
CM10X		0.843	0.829	0.78	0.282	0.268	0.219	0.25633	0.03308
Blank		0.573	0.533	0.577					
	Ave.	0.561							

72 hr	OD450nm				minues Blank			Ave.	Std.
Control	xLPS, xFBS	0.627	0.614	0.642	0.066	0.053	0.081	0.06667	0.01401
LPS	xFBS	0.657	0.62	0.626	0.096	0.059	0.065	0.07333	0.01986
CM1X		0.677	0.704	0.702	0.116	0.143	0.141	0.13333	0.01504
CM5X		0.713	0.74	0.763	0.152	0.179	0.202	0.17767	0.02503
PT5X		0.624	0.628	0.638	0.063	0.067	0.077	0.069	0.00721
CM10X		0.793	0.813	0.86	0.232	0.252	0.299	0.261	0.03439
Blank		0.573	0.533	0.577					
	Ave.	0.561							

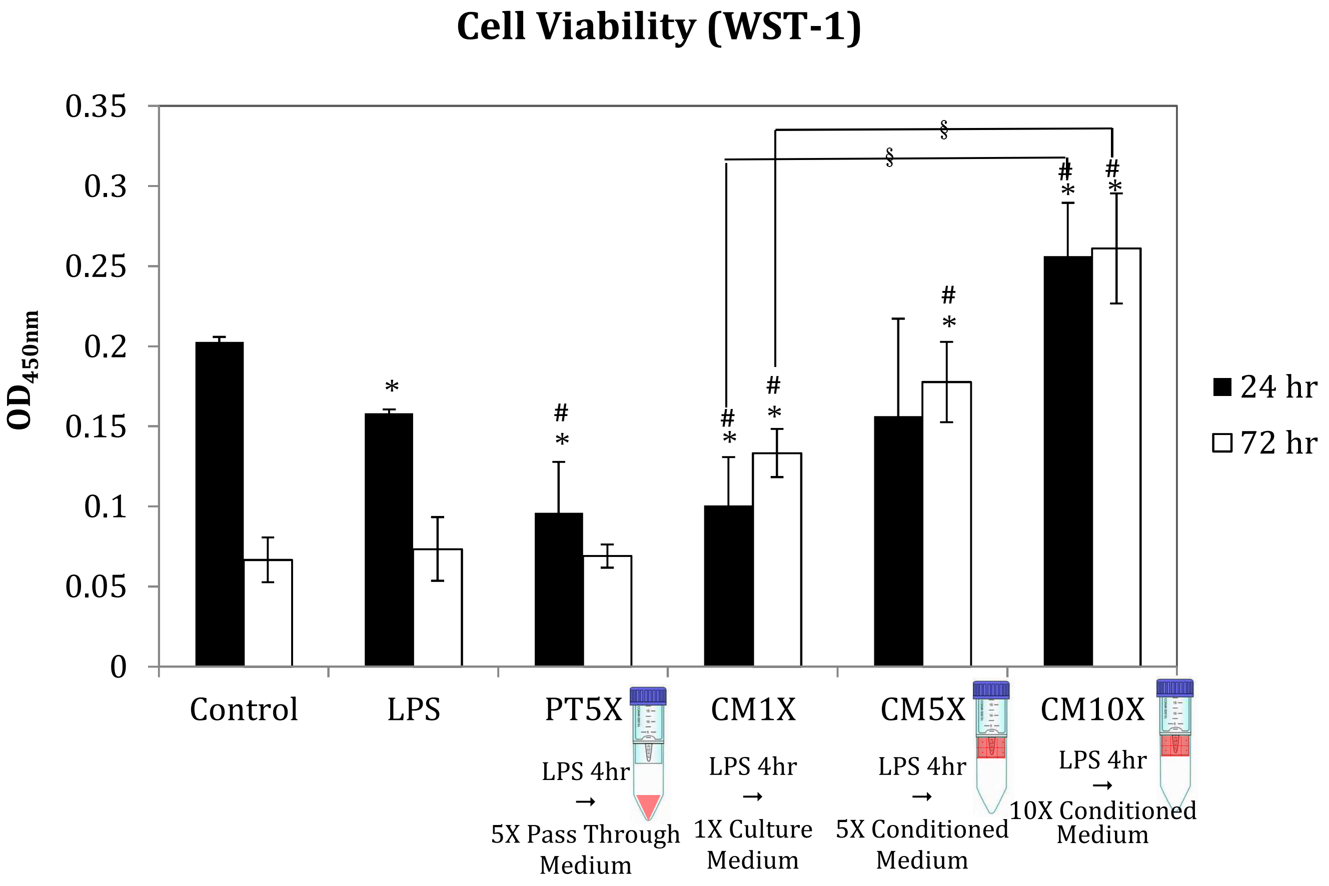

Supplement: S9 Data — (PDF) [file pone.0205563.s009.pdf]
